# Species distribution modelling of benthic amphipod crustaceans in the deep North Atlantic under climate change

**DOI:** 10.1038/s41598-025-26442-x

**Published:** 2025-11-12

**Authors:** Karlotta Kürzel, Craig P. Hammock, Vanessa Pitusi, Saskia Brix, Anne-Nina Lörz

**Affiliations:** 1 Institute of Marine Ecosystem and Fishery Science (IMF), Center for Earth System Research and Sustainability (CEN), University of Hamburg, Große Elbstraße 133, 22767 Hamburg, Germany; 2 School of Computer Science and Mathematics, Liverpool John Moores University, Liverpool, UK; 3 UpGrad Education Pvt. Ltd., Mumbai, India; 4 The Arctic University Museum of Norway, UiT The Arctic University of Norway, Tromsø, Norway; 5 Senckenberg Am Meer, German Centre for Marine Biodiversity Research (DZMB), c/o Biozentrum Grindel, Universität Hamburg, Martin-Luther-King-Platz 3, 20146 Hamburg, Germany; 6 Department Marine Research, Senckenberg Am Meer (SaM), Südstrand 40, 26382 Wilhelmshaven, Germany

**Keywords:** Amphipoda, Species distribution modelling, North Atlantic, Deep sea, Crustacea, Climate change, Biodiversity, Climate-change ecology, Ecological modelling

## Abstract

Climate-driven changes in environmental factors influence vulnerable North Atlantic deep-sea (> 200 m depth) benthic ecosystems, leading to species range shifts, habitat loss, or extinctions. Amphipod Crustaceans play a crucial role in deep-sea ecosystems, contributing to food web stability and nutrient cycling. However, their large-scale distributions on species level remain poorly understood. In this study, we created species distribution models (SDMs) of 55 North Atlantic deep-sea amphipods in the present day, medium-term (2050–2060) and long-term (2090–2100) future, utilising best, likely, and worst shared socioeconomic pathways (SSPs) scenarios. The results show species-specific responses to climate change. Over half of the amphipod species expand their habitat in some scenarios, while others face habitat loss. Contrasting habitat likeliness is represented by species of the same genera. Additionally, some species experience habitat shifts, particularly northward and towards the Greenlandic coast. Glacial meltwater influx and increased nutrient availability could enhance habitat suitability in certain regions. Poleward shifts are theorised to be temperature-driven. These changes influence biodiversity, food web dynamics, and ecosystem stability. This study provides a baseline for assessing future changes in North Atlantic amphipod distributions. The findings emphasise the need for conservation strategies and taxonomy in predicting ecosystem responses to climate change.

**Supplementary Information:**

The online version contains supplementary material available at 10.1038/s41598-025-26442-x.

## Introduction

Commonly found throughout the benthic realm^1–5^, amphipods provide critical functions that underpin marine food webs, in addition to performing functions integral to deep-sea ecosystems. Amphipods are essential to deep-sea nutrient cycling as they help facilitate the transfer of organic matter between benthic and pelagic food webs^6^. They feed on sinking particulate organic matter, of which the nutrients are subsequently reintroduced into the water column and higher trophic levels when amphipods are consumed by predators^7,8^. This way, amphipods facilitate the transfer of energy and nutrients across trophic levels, contributing substantially to the functioning and stability of deep-sea ecosystems^6,9^. Many deep-sea amphipod species are also bioturbators and thus key ecosystem engineers, where tube-building and burrow construction create small-scale habitat heterogeneity and enhance nutrient availability^6^. Despite their ecological importance, many deep-sea amphipod species are suited to specific environmental niches, making them vulnerable to changing conditions^10^.

However, deep-sea environments, defined as any marine water mass deeper than 200 m^11^ are vulnerable to a changing climate or anthropogenic pressures^12^. As a sink for carbon and excess heat, the increasing quantities of greenhouse gases in the atmosphere, including carbon-dioxide (CO_2_) and methane (CH_4_) impact the marine environment directly, and indirectly. This causes multiple changes, rapid changes in temperature^13^, decreasing oxygen levels^14^, ocean acidification^15^, and alterations in ocean circulation^16^. The North Atlantic provides a particularly vulnerable deep-sea environment, given its integral role in ocean circulation. The Atlantic Meridional Overturning Circulation (AMOC), where deep, colder water is transported southwards and shallow, warmer water moves northwards^17^, influences temperature and salinity among other climate patterns globally^18^. Disruption or slowdown of the AMOC risks widespread consequences in deep-sea environmental conditions, with substantial impacts on marine ecosystems^19^.

Environmental pressures caused by climate change and anthropogenic pressures can have wide ranging effects that impact upon species distribution, community structures^20–22^ and ecosystem function at varying spatial scales^23,24^. Consequences may include altered geographical and bathymetric distribution ranges^25,26^, health declines^27^, and even species extinctions^28^. Since species are interconnected through the food web, the health of one species has the potential to influence the entire ecosystem. The effects could cascade across trophic, whereby the removal of one species with an integral ecosystem function causes a decline in others that relied upon it for nutrients^39^. Amphipods may be particularly prone to the effects of climate change, as they belong to the group Pericarida, which do not have larvae. This crustacean suborder has a brooding lifestyle, limiting their dispersal and thus may affecting their response to changing environmental conditions as seen in other marine taxa^29,30^. This response at the lower trophic levels can thus indirectly affect the overall species composition, species abundance, and community structure^31^.

To understand how marine fauna are adjusting to the changing climate, towards informing conservation and mitigation efforts, a present-day knowledge of their distribution and diversity is fundamental. However, comparatively little is known about the deep-sea biome, given the inaccessibility of the region and high economic costs of exploratory studies^12,32,33^. For example, it is estimated that 91% of all marine species are currently undescribed^34^. Given the infrequency and paucity of deep-sea sampling efforts, modelling is commonly used to fill this deep-sea knowledge gap. A common approach is to produce species distribution models (SDMs), in which statistical and machine learning techniques are used to predict species distributions based on occurrence data and environmental variables^35^. In the North Atlantic, this approach has been used to model the distribution of a limited number of benthic taxa and species. For example, Burgos et al.^36^ used SDMs to predict the distribution of vulnerable marine ecosystem indicator taxa such as corals, sponges, and sea pens, while Reiss et al.^37^ applied this approach to model 14 different benthic species from multiple taxa, including brittle stars, polychaetes, and bivalves. Other studies have clustered amphipod distributions around Iceland by their environmental preference, to assess their regional-scale diversity^38^. Despite these efforts, modelling of deep-sea species is piecemeal, and there is scope for further efforts given the scattered and disparate nature of current distribution knowledge for species in the deep-sea. To the authors knowledge, this study is the first to analyse large scale distribution under different climate change scenarios of amphipod on a species level.

Despite their critical role and ecosystem function, little is known about the current distribution of various amphipod species in the deep sea, and how this might change in the future. Therefore, the aim of the study is to identify the current and future distribution of benthic amphipod species in the deep North Atlantic using species distribution modelling (SDM) and open-source occurrence data. Models will be generated for present, medium- (2050–2060) and long-term predictions (2090–2100) based on three climate change scenarios (Shared Socioeconomic Pathway (SSP) 1–1.9, SSP 2–4.5, and SSP 5–8.5). As such, this study will help address the knowledge gap of deep-sea amphipod distributions, provide a baseline for evaluating future changes, support targeted conservation efforts and deepen the understanding of the cascading effects of environmental change on benthic food webs and ecosystem functions in the deep North Atlantic.

## Materials & methods

### Dataset

**Fig. 1:**
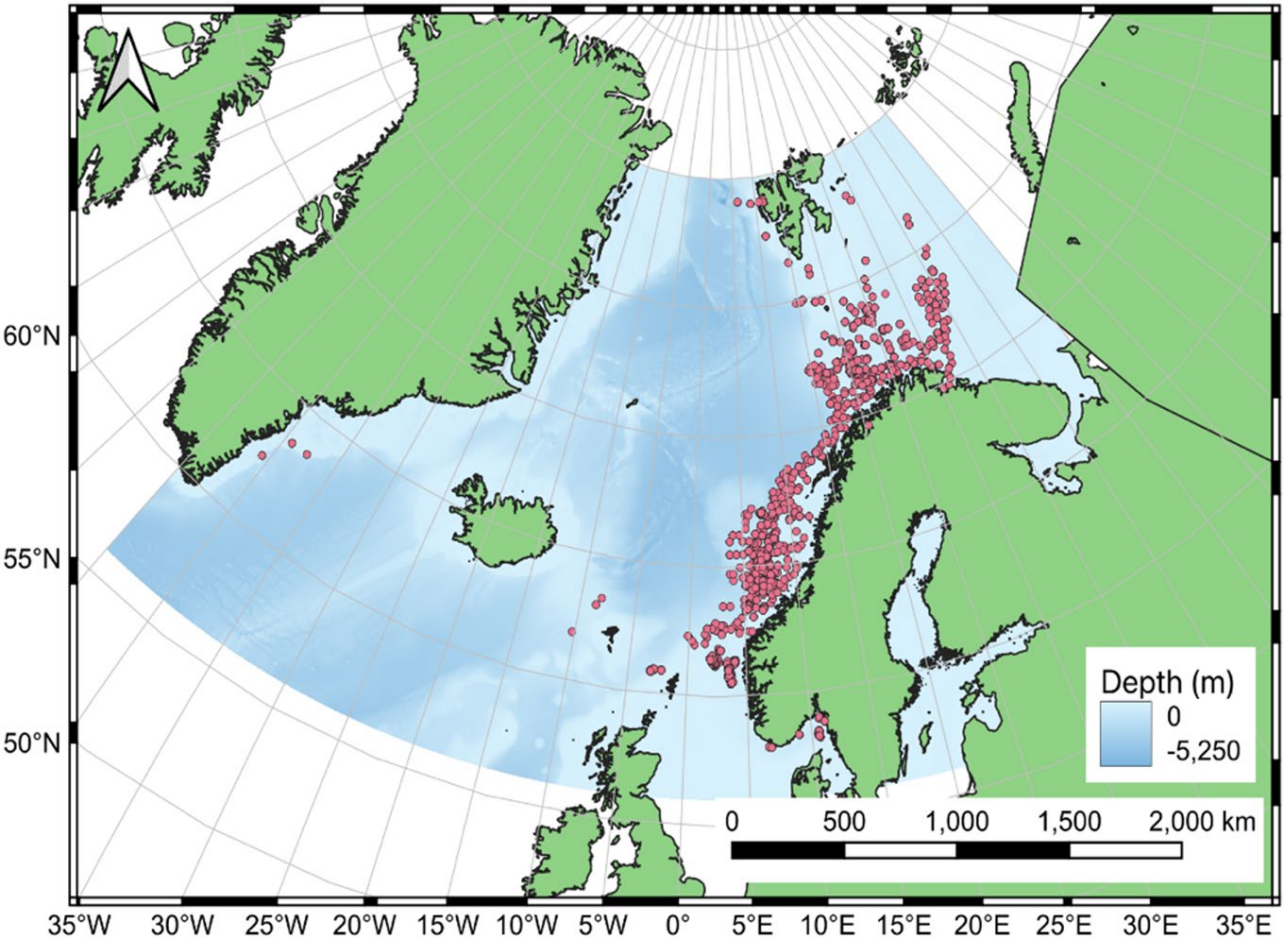
Map of species occurrence points after performing all cleaning and filtration steps^40^ (Mapping: Created with QGIS, version 3.40.5-Bratislava, https://www.qgis.org).

For this study, amphipod occurrence data were sourced from the Ocean Biogeographic Information System (OBIS) using the mapper tool provided on the website^39^. The obtained dataset was filtered in Python to only include records between the latitudes 56°N and 80°N and longitudes between 45°E and 45°W. This region has been previously predicted by Burgos et al. 2020 as one of vulnerable marine ecosystems and high biodiversity. To focus on the deep sea, only records of depths of more than 200 m were included, and purely pelagic amphipod species were excluded to ensure the best suitability for modelling with environmental layers. This was further addressed by cross-referencing the sampled depth of the records with bathymetry data from the General Bathymetric Chart of the Oceans (GEBCO) for the seafloor^40^. If the difference between the two depths exceeded 5%, the record was assumed to be non-benthic and it was excluded. Additionally, given that previous studies have demonstrated a decline in model robustness and evaluation metrics, such as AUC (Area Under the Curve), with insufficient sample sizes (e.g.^41–43^), species with less than 200 occurrence records were excluded from the dataset. Furthermore, any records collected outside of the years 2000–2020 was excluded as only this period aligned with the temporal range of the Bio-ORACLE environmental data layers. The final occurrence data set contained 55,941 records from multiple sampling expeditions and studies (Fig. 1)^44–58^.

Environmental data were sourced from Bio-ORACLE^59,60^ which provides raster layers widely used in species distribution modelling. Benthic data were extracted for average depths. Current environmental data covered the periods 2000–2010 and 2010–2020, while future projections utilized the periods 2050–2060 and 2090–2100, enabling both medium- and long-term analysis. Future environmental data from Bio-ORACLE included modelled variables based on SSPs, representing greenhouse gas emission scenarios under different policy decisions. This study used three scenarios: SSP 1–1.9 (low emissions, sustainable future), SSP 2–4.5 (moderate emissions, likely scenario), and SSP 5–8.5 (high emissions, worst case)^61^.

Variance Inflation Factor (VIF) analysis was performed to address multicollinearity by excluding highly correlated variables. These variables included depth, selected benthic variables (dissolved oxygen, nitrate, phosphate, salinity, temperature), and selected surface variables (iron, phosphate, primary productivity, salinity). The final environmental dataset for current and future scenarios retained benthic and surface silicate (mmol/m^3^) and current velocity (m/s), as well as surface chlorophyll (mmol/m^3^), dissolved oxygen (mmol/m^3^), and nitrate (mmol/m^3^). Additionally, benthic primary productivity (mmol/m^3^) and iron (mmol/m^3^) were included. These variables were selected based on their known or potential influence on peracarid and amphipod distribution. Although the specific environmental drivers of amphipod distribution remain poorly understood, previous studies have employed similar predictors to identify factors shaping species distribution and abundance^4,38,62^. Other studies conducting niche breadth analyses have also highlighted their relevance in defining amphipod niche width^10^. Additionally, as the deep-sea is closely connected to surface processes through the vertical flux of organic matter^63,64^, surface variables were included to account for their potential influence on benthic amphipods.

### Resolving sampling bias 

As samples are often collected in more accessible areas where the logistical costs of sampling expeditions are lower, marine datasets are often inherently biased which requires consideration for the production of accurate and robust SDMs. In this study, a target-group sampling approach was deployed, following the works of Phillips et al.^65^ and Fourcade et al.^66^. This approach uses environmental background points that are sampled with the same bias as the presence-only dataset, to be used as a model environmental input. To do this, an estimate of overall sampling effort is calculated using a Kernel Density Estimate (KDE) of presence point distribution using QGIS. A kernel bandwidth of 100 km, a grid cell size of 800 m and a quartic kernel function was used to balance spatial resolution with computing power. This KDE was then normalised in Python, such that cells that had zero values, because they persisted in regions which were not previously sampled, were given a negligible value such that there was still a small chance of these being selected by background point sampling. The probability map, produced using the KDE approach, was consequently sampled to produce background points. In this study, 50,000 background points were produced, balancing the risk of model overfitting and processing power whilst ensuring the number is large enough to represent the environmental range in the study area accurately^67^. 

Following this, individual datasets were produced for each species, with each record representing a geographical point where presence for that species had been determined by sampling. Background points were merged onto these individual datasets and labelled within each of them. The environmental data was sampled using these datasets, such that each record contained a column for each environmental variable at the time of collection.

### Species distribution modelling 

The species distribution modelling was conducted using the Maximum Entropy algorithm (MaxEnt). This approach is suitable for presence-only data as it combines samples with background/pseudo-absence points and has shown above-average performance, thus representing a standardised approach in marine species distribution modelling^68,69^. In this study, the elapid Python package^70^ was used.

Three-fold cross-validation was applied to all models, and the average Area Under the Curve (AUC) score across folds were used for model evaluation. Various combinations of feature classes and regularisation multipliers were tested. The hinge feature class, which yielded the highest mean, minimum, and maximum AUC scores, was selected for further modelling. Additionally, a regularisation multiplier of two was chosen for downstream modelling with the MaxEnt algorithm. This selection was informed by previous research indicating that regularisation multipliers two to four times higher than the default (1.0) can mitigate overfitting while maintaining strong predictive performance in habitat suitability and species distribution modelling^71^.

Models were first created for each species under present-day conditions, using the individual presence points alongside background points derived utilising the target-group sampling approach. Afterwards, future SDMs for each species for the medium-term and long-term under all three SSPs were created utilising the predicted environmental data from the Bio-ORACLE layers. These models used the same setup as the optimised present-day models. The SDMs were visualised using Python rasterio v1.3.10, matplotlib v3.9.2, and QGIS 3.40.5-Bratislava. Zonal statistics depicting suitable habitat area size were calculated using rasterio numpy v1.26.4, while descriptive statistics were visualised using seaborn v0.13.2. The nomenclature used in this study follows the World Register of Marine Species^72^.

## Results

In total, present and future models for 55 amphipod species (Table 1) were created with an average AUC score of 0.85 (Table S1).

**Table 1:** List of all 55 amphipod species included for modelling, sorted alphabetically.

Species	Authority	Family	Sample size
*Aceroides (Aceroides) latipes*	(G.O. Sars, 1883)	Oedicerotidae	386
*Ampelisca aequicornis*	Bruzelius, 1859	Ampeliscidae	646
*Ampelisca eschrichtii*	Krøyer, 1842	Ampeliscidae	317
*Ampelisca gibba*	G.O. Sars, 1883	Ampeliscidae	472
*Ampelisca macrocephala*	Liljeborg, 1853	Ampeliscidae	696
*Ampelisca odontoplax*	G. O. Sars, 1879	Ampeliscidae	619
*Ampelisca pusilla*	G.O. Sars, 1891	Ampeliscidae	1085
*Amphilochus manudens*	Spence Bate & Westwood, 1862	Amphilochidae	853
*Arrhis phyllonyx*	(M. Sars, 1858)	Oedicerotidae	800
*Autonoe longipes*	(Liljeborg, 1852)	Aoridae	658
*Autonoe megacheir*	G. O. Sars, 1879	Aoridae	1045
*Bathymedon longimanus*	(Boeck, 1871)	Oedicerotidae	749
*Bathymedon saussurei*	(Boeck, 1871)	Oedicerotidae	370
*Bruzelia typica*	Boeck, 1871	Synopiidae	303
*Byblis crassicornis*	Metzger, 1875	Ampeliscidae	1568
*Byblis gaimardii*	(Krøyer, 1846)	Ampeliscidae	371
*Eriopisa elongata*	(Bruzelius, 1859)	Eriopisidae	7427
*Halice abyssi*	Boeck, 1871	Pardaliscidae	570
*Haploops setosa*	Boeck, 1871	Ampeliscidae	3202
*Haploops tubicola*	Liljeborg, 1856	Ampeliscidae	1571
*Harpinia abyssi*	G.O. Sars, 1879	Phoxocephalidae	743
*Harpinia antennaria*	Meinert, 1890	Phoxocephalidae	2769
*Harpinia crenulata*	(Boeck, 1871)	Phoxocephalidae	1115
*Harpinia laevis*	G.O. Sars, 1891	Phoxocephalidae	274
*Harpinia mucronata*	G. O. Sars, 1879	Phoxocephalidae	2087
*Harpinia pectinata*	G.O. Sars, 1891	Phoxocephalidae	5612
*Harpinia plumosa*	(Krøyer, 1842)	Phoxocephalidae	427
*Harpinia propinqva*	G.O.Sars, 1891	Phoxocephalidae	235
*Hippomedon denticulatus*	(Spence Bate, 1857)	Tryphosidae	795
*Hippomedon propinqvus*	G.O. Sars, 1890	Tryphosidae	499
*Idunella aeqvicornis*	(G.O. Sars, 1877)	Liljeborgiidae	633
*Ischyrocerus megacheir*	(Boeck, 1871)	Ischyroceridae	270
*Laetmatophilus tuberculatus*	Bruzelius, 1859	Podoceridae	717
*Leptophoxus falcatus*	(G.O. Sars, 1883)	Phoxocephalidae	1092
*Liljeborgia fissicornis*	(M. Sars, 1858)	Liljeborgiidae	922
*Liljeborgia pallida*	(Spence Bate, 1857)	Liljeborgiidae	441
*Lysianassa plumosa*	Boeck, 1871	Lysianassidae	413
*Medicorophium affine*	(Bruzelius, 1859)	Corophiidae	211
*Melphidippa borealis*	Boeck, 1871	Melphidippidae	266
*Neohela monstrosa*	(Boeck, 1861)	Unciolidae	832
*Nicippe tumida*	Bruzelius, 1859	Pardaliscidae	1811
*Nototropis nordlandicus*	(Boeck, 1871)	Atylidae	796
*Oediceropsis brevicornis*	Lilljeborg, 1865	Oedicerotidae	495
*Paraphoxus oculatus*	(G. O. Sars, 1879)	Phoxocephalidae	394
*Syrrhoe crenulata*	Goës, 1866	Synopiidae	758
*Themisto abyssorum*	(Boeck, 1871)	Hyperiidae	216
*Tmetonyx cicada*	(Fabricius, 1780)	Uristidae	2521
*Tmetonyx similis*	(G.O. Sars, 1891)	Uristidae	233
*Tryphosella horingi*	(Boeck, 1871)	Tryphosidae	206
*Tryphosites longipes*	(Spence Bate & Westwood, 1861)	Tryphosidae	684
*Unciola leucopis*	(Krøyer, 1845)	Unciolidae	422
*Unciola planipes*	Norman, 1867	Unciolidae	657
*Urothoe elegans*	Spence Bate, 1857	Urothoidae	1745
*Westwoodilla caecula*	(Spence Bate, 1857)	Oedicerotidae	516
*Xenodice frauenfeldti*	Boeck, 1871	Podoceridae	426

**Fig. 2:**
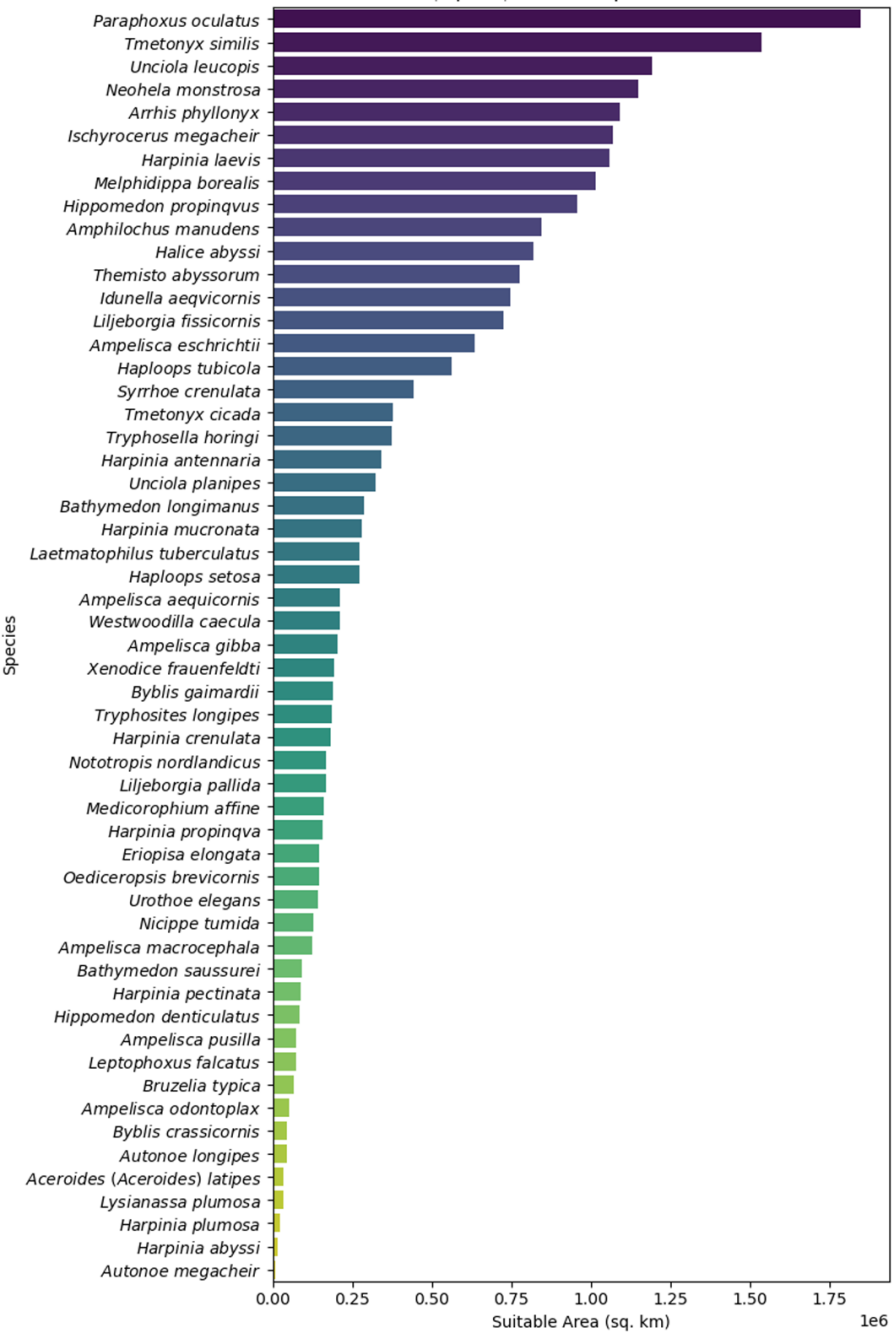
Suitable Area Size per species under current environmental conditions (2000–2020).

**Fig. 3:**
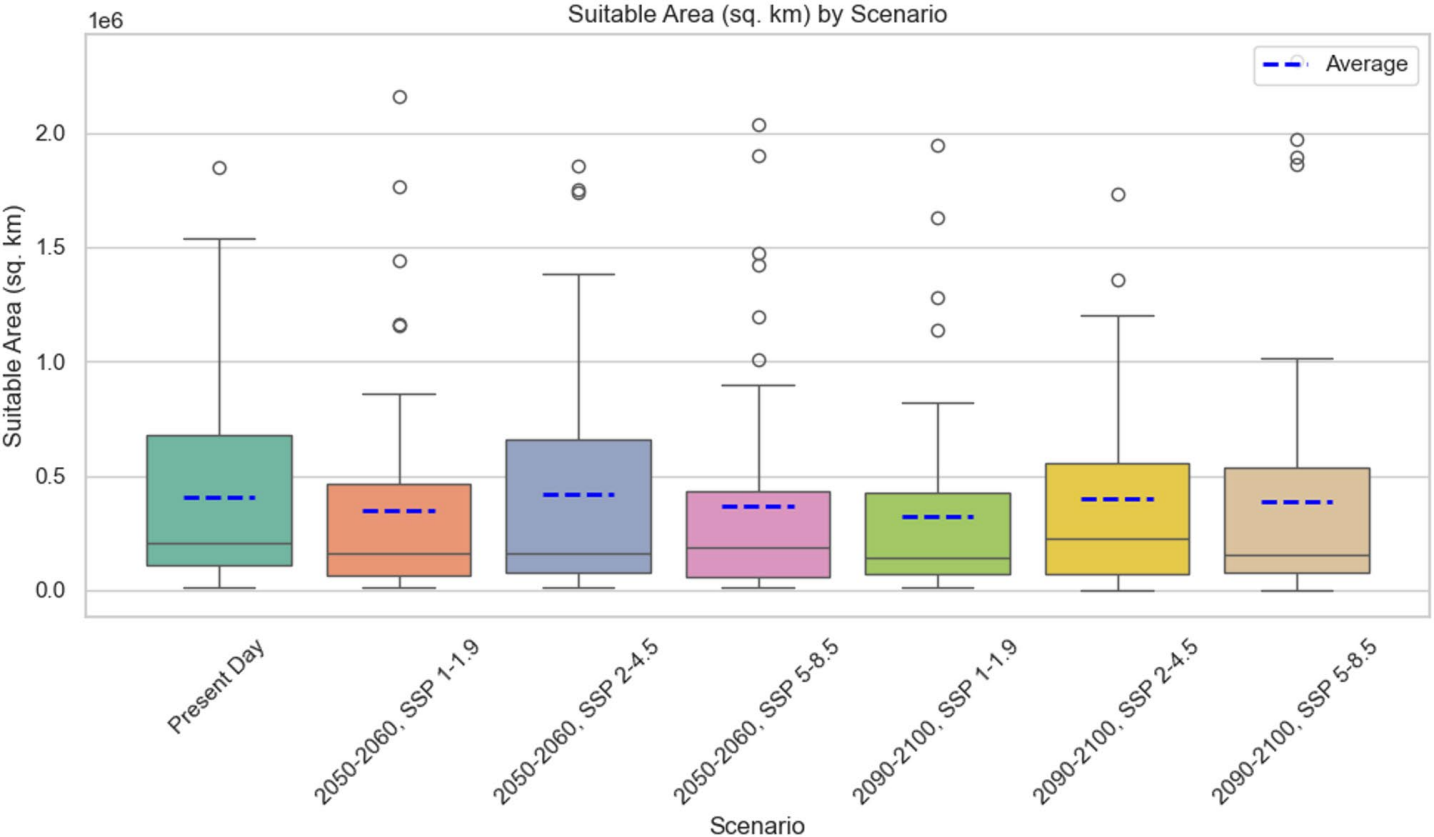
Suitable habitat area for all species for the present day, SSPs and future scenarios for all species for the present day, SSPs, and future scenarios.

For the current scenario, *Paraphoxus oculatus* had the largest suitable habitat area with 1,845,852 km^2^ (for probability threshold 0.70) while *Autonone magacheir* has the smallest suitable area with 10,471 km^2^ (Fig. 2). On average, the suitable habitat area was 408,296 km^2^ (Fig. 3). In comparison, for the medium-term 2050–2060 timeframe under SSP 1–1.9, the average suitable habitat area decreased to 350,432 km^2^, with lower quartile at 63,527 km^2^ and upper quartile at 464,116 km^2^ (min: 10,475 km^2^, max: 2,157,231 km^2^). For the same period under the SSP 2–4.5 scenario, the average habitat area increased to 420,912 km^2^, with lower quartile at 79,770 km^2^ and upper quartile at 656,716 km^2^ (min: 9440 km^2^, max: 1,853,475 km^2^). In contrast, under the SSP 5–8.5 scenario for 2050–2060, the average habitat area decreased to 365,381 km^2^, with lower quartile at 60,141 km^2^ and upper quartile at 432,233 km^2^ (min: 14,876 km^2^, max: 2,033,417 km^2^; Fig. 3).

For the long-term 2090–2100 timeframe, comparing SSP 1–1.9 to the short-term scenario, the average suitable habitat area decreased to 320,006 km^2^, with lower quartile at 70,537 km^2^ and upper quartile at 427,583 km^2^ (min: 12,631 km^2^, max: 1,945,080 km^2^). Comparing SSP 2–4.5 from 2090–2100 to the 2050–2060 period, the average habitat area decreased to 397,261 km^2^, with lower quartile at 70,353 km^2^ and upper quartile at 553,617 km^2^ (min: 868 km^2^, max: 1,731,375 km^2^). Finally, for the SSP 5–8.5 scenario, the average area increased to 385,685 km^2^ in 2090–2100, with lower quartile at 76,937 km^2^ and upper quartile at 537,744 km^2^ (min: 2668 km^2^, max: 2,311,682 km^2^; Fig. 3).

Out of the 55 species examined, 14 species (with a probability threshold of 0.7) were projected to lose suitable habitat across all future scenarios. However, this number doubled to 30 species when considering five out of six scenarios, highlighting a broader trend of habitat loss across a range of future conditions.

Detailed modelling for each species is shown in the Supplementary Information (Figs. S1–S55). The broader trends are visualised in Figs. 4 and 5, where the number of species in each area and the change between SDMs for different SSP scenarios and the present day are depicted. These summarise a broad range of geographic trends that were observed.

**Fig. 4:**
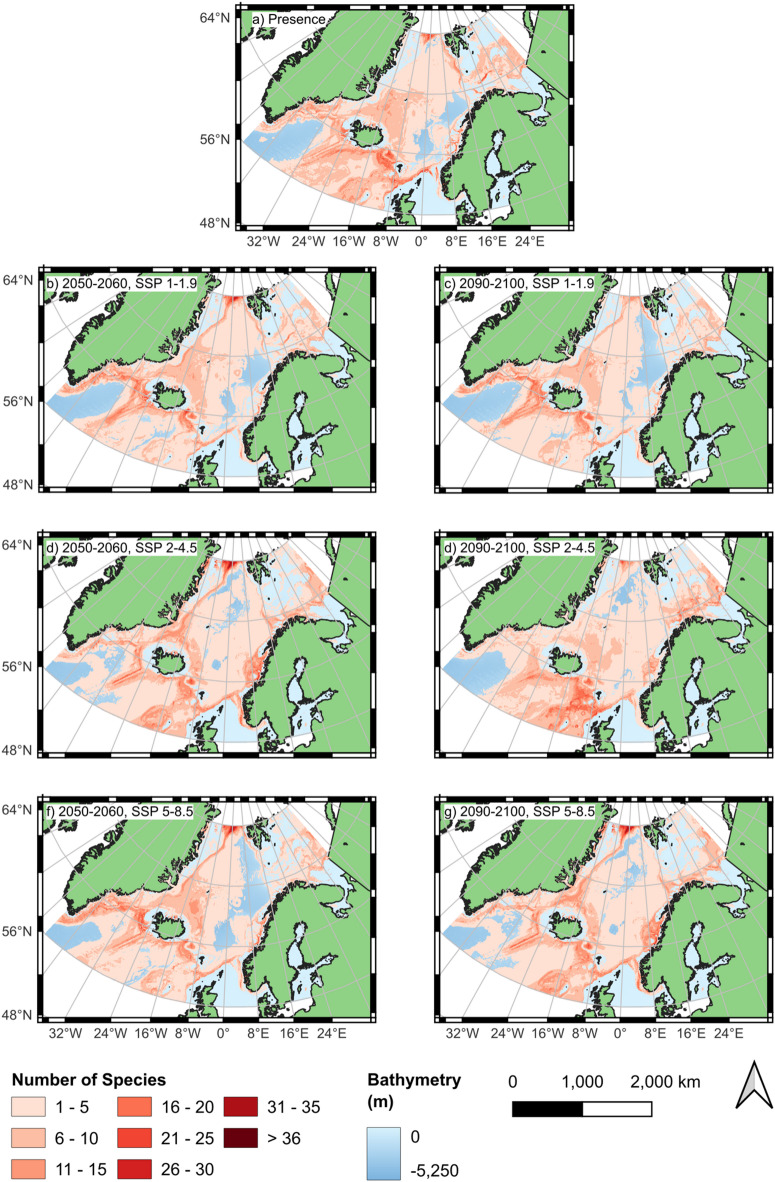
Number of species modelled present in each area based on all 55 individual species distribution models (SDMs). (Mapping: Created with QGIS, version 3.40.5-Bratislava, https://www.qgis.org).

**Fig. 5:**
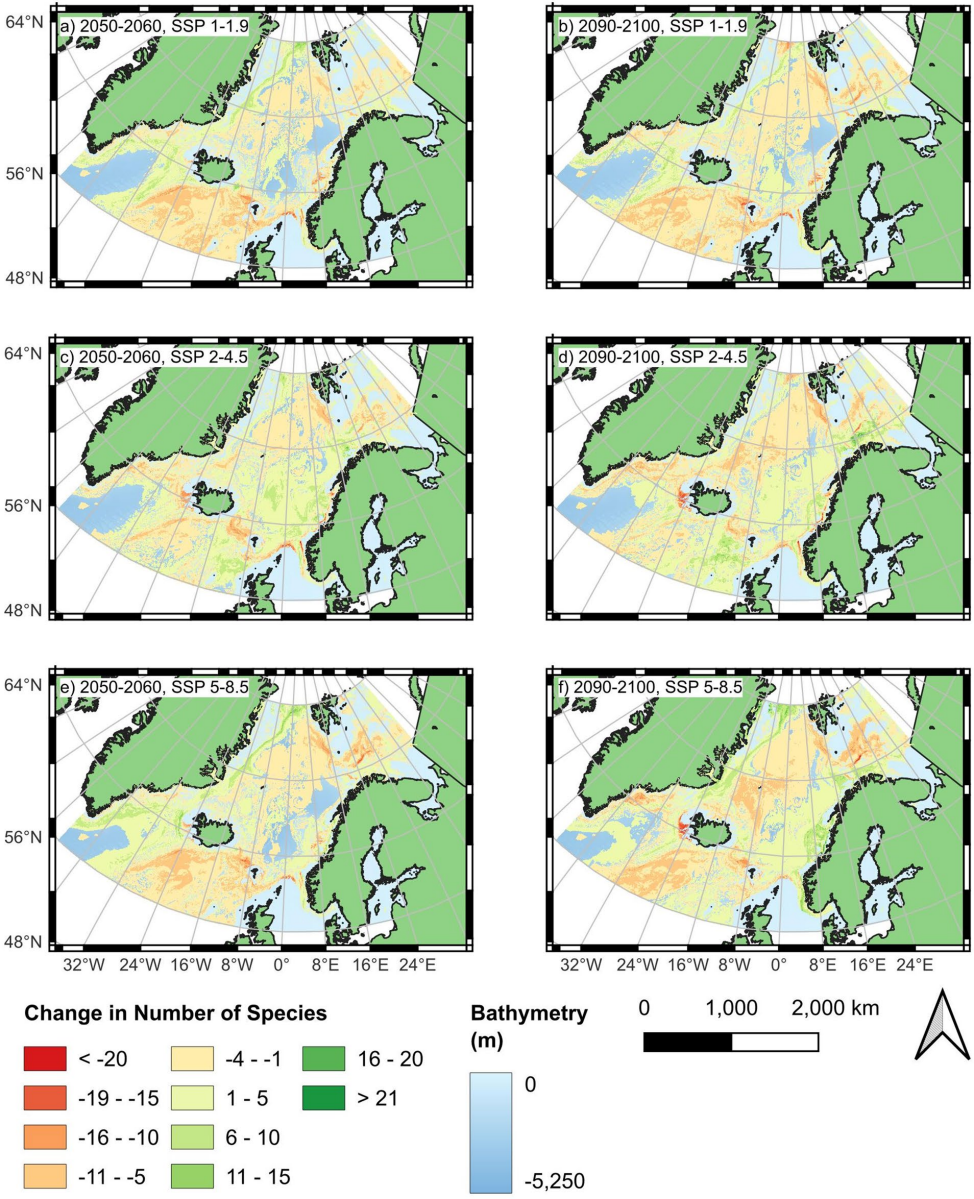
Number of species modelled present in each area based on all 55 individual species distribution models (SDMs). (Mapping: Created with QGIS, version 3.40.5-Bratislava, https://www.qgis.org).

The northeastern coast of Greenland shows an increase in habitat suitability for a number of amphipods, across all SSP scenarios and timeframes. This trend is particularly pronounced under the extreme scenario SSP5–8.5 for the period 2090–2100 (Figs. 4 and 5). Examples of species exhibiting increased habitat suitability in this region in at least some scenarios include *Idunella aequicornis* and *Aceroides* (*Aceroides*) *latipes* (S20 and S36, Supplement).

The southwestern part of the study area exhibits a complex response to future climate change scenarios, with increases in species numbers observed along the western side of the Reykjanes Ridge but notable decreases in some coastal regions. A high number of species (15–20) are projected to lose habitat suitability in the coastal waters of southeastern Greenland under both the SSP2–4.5 and SSP5–8.5 scenarios. Similarly, areas adjacent to the western coast of Iceland show pronounced reductions in species numbers, particularly under these higher-emission scenarios (Figs. 4 and 5). *Laetmatophilus tuberculatus* (S8, Supplement) is an example of this declining trend, exhibiting a loss of suitable habitat across both regions.

However, slight increases in species numbers are projected in parts of the Irminger Sea, with the most pronounced changes occurring under SSP5–8.5 (Figs. 4 and 5). *Tmetonyx cicada* (S13, Supplement) is an example of this pattern. While for habitat suitability along the Reykjanes Ridge is projected to decline especially in 2090–2100 under SSP5–8.5, this species shows increasing suitability within the Irminger Sea, particularly along the southern coast of Greenland (Figs. 4 and 5).

In the southeastern part of the study area, several regions exhibit a decrease in species richness. Along the eastern side of the Reykjanes Ridge, a notable decline of approximately 10–15 species is projected across all SSP scenarios and time periods (Figs. 4 and 5). This pattern is exemplified by *Ampelisca gibba* (S1, Supplement) and *Harpinia crenulata* (S7, Supplement), whose suitable habitat within the Reykjanes Ridge region diminishes substantially and, in some scenarios, disappears entirely.

Similarly, along the Iceland–Faroe Ridge, a decrease of approximately 15–20 species is projected across all scenarios and time periods (Figs. 4 and 5). Species exhibiting this pattern include *Tmetonyx cicada* (S13, Supplement) and *Tryphosites longipes* (S14, Supplement), both of which consistently show a decline in habitat suitability under all SSPs and timeframes. For *Tmetonyx cicada*, only a small area near the centre of the ridge remains suitable, whereas the habitat suitability of *Tryphosites longipes* disappears entirely from this region under both the SSP2–4.5 and SSP5–8.5 scenarios.

The area between northern Iceland and the southwest of Svalbard shows a marked decrease in habitat suitability for numerous species across all scenarios and time periods. This decline is particularly pronounced, with reductions exceeding 15 species in all scenarios except SSP1–1.9 for the period 2050–2060 (Figs. 4 and 5). Examples of species exhibiting this pattern include *Unciola leucopis* (S39, Supplement) and *Hippomedon propinquus* (S30, Supplement), both of which consistently show a decline in habitat suitability across all scenarios. Notably, both species lose all suitable habitat in this region under SSP2–4.5 by 2090–2100.

## Discussion

### Species response to climate change

Predicted models of species distribution under future scenarios show a complex and varied response of amphipods to climate forcing, echoing the findings of other studies modelling the distribution of benthic species. For example, Moraitis et al.^73^ investigated the distribution of benthic indicator species under climate change scenarios and found reduction in habitat area for three of these species, while the remaining species showed a habitat expansion. Similarly, Xu et al.^74^ investigated five benthic species and determined that two of them would experience an increase in suitable habitat under future climate conditions. However, these studies and our findings contrast with the hypotheses outlined by Sandulli et al.^75^ and Weinert et al.^76^, which suggest that benthic species will generally undergo declines under future climate scenarios.

In our study, relatively few amphipod species project a decline in suitable habitat area under all climate scenarios, and just over half showed a decline under most scenarios. Examples of species that show a decline, such as *Ampelisca gibba* or *Nicippe tumida*, indicate the role of bathymetric features in providing biogeographical barriers that inhibit migration within the North Atlantic. These ridges separate distinct ocean basins with unique physicochemical conditions, providing temperature and salinity gradients, and serve as areas with particularly strong interactions in water masses that lead to oceanographic conditions that can shape marine organism distribution. In the North Atlantic, these include the Reykjanes Ridge and the Greenland-Iceland-Faroe (GIF) Ridge, the latter of which is known particularly for unique environmental conditions hosting specialised benthic fauna^77–82^.

In contrast, many species are shown to increase their suitable habitat area across at least some future climate scenarios, echoing the findings in other benthic fauna such as Gastropoda (e.g.^83^), other Crustacea (e.g.^84^) and corals (e.g.^85^). One common pattern of habitat expansion is a latitudinal shift toward the continental margins of Greenland, where climate impacts on marine biogeochemistry and nutrient cycling are apparent^86^. Temperature increases result in increased glacial melt, leading to more glacial meltwater entering the ocean through run-off. This freshwater is rich in nutrients such as nitrogen^87^, phosphate^88^, and iron^86,89^, and thus impacts upon local nutrient availability, increases primary productivity, and enhances habitat suitability. The movement and increase in suitable habitat area for many amphipod species likely reflects the predicted increase in nutrient availability and primary productivity proximal to the Greenlandic coast.

While a number of species show an increase or decrease in total suitable habitat area across the study region, the pattern often reflects a migration of suitable habitat in response to changing environmental conditions. One common pattern of a migration in suitable habitat area is a northward shift towards the pole in many species (e.g*., Westwoodilla caecula*). As hypothesised with other benthic species (e.g.^90^), poleward shifts are primarily driven by temperature changes, as species have thermal tolerances that physiologically restrict their distribution^91^. As temperatures of waters closer towards the poles are cooler, species migrate to the waters which fit their temperature preference (e.g.^90^). Temperature, alongside other climate forced oceanographic processes, will also lead to Atlantification, whereby Arctic and subpolar waters become more similar to the Atlantic waters in environmental characteristics such as salinity^92^. The wider impact of this includes the loss of stratification in the deep-sea North Atlantic, whereby the colder and less saline Arctic Ocean that mixes across depth with the warmer and saltier Atlantic Ocean become more alike. Critically, this impacts upon the food web, with changes in nutrient availability and primary productivity across the region^93^, that the SDMs reflect.

The heterogeneity of species-level responses to climate scenarios outlines the importance of analysing distributional patterns at the species level, in amphipods and in other benthic species. The variation in SDMs of the same family is consistent with Lörz et al.^10^, who reported that the environmental niche of many examined amphipod species was not directly transferable to other species within the same family. While acknowledging that some families were underrepresented in terms of species numbers in this study, it remains essential to not only increase sampling efforts but also ensure accurate taxonomic identification at a species level. Many museums and natural history collections hold thousands of amphipod specimens that currently cannot be included in ecological or modelling analyses due to identification being limited to the order level. Applying an integrative taxonomic approach will deepen our understanding of species distributions and potential implications on the entire ecosystem, particularly in the context of ongoing environmental changes.

### Ecological and conservation implications

Changes in amphipod distribution could have cascading effects on other organisms within the deep-sea, disrupting nutrient cycling and energy flows throughout the water column. A decline in amphipods in certain regions could contribute to the destabilization of the food web. Conversely, a significant influx of amphipod species into new areas may lead to population increases in their predators, which could subsequently increase competition pressure on other species. This could alter community structures and contribute to localized biodiversity loss by favouring certain species that thrive on the new abundance of prey^94^. As different taxa contribute to ecosystem stability and function, a reduction in amphipod populations may therefore diminish critical processes of organic matter transport, oxygenation, nutrient cycling, and secondary production, with cascading effects across trophic levels.

The impact on the deep-sea ecosystem emphasises the need for conservation and management efforts in regions where habitat suitability is projected to decline. The GIF ridge is an area with particular cause for concern, given the unique environmental conditions and faunal diversity in the region^79^. Changes in sediment composition and water chemistry south of the GIF Ridge have already been linked to biodiversity shifts in other benthic communities^95,96^, whilst SDMs in this study show the habitat as becoming unsuitable for species such as *Ampelisca gibba*. The loss of biodiversity in the GIF Ridge could lead to the restructuring of a unique marine community, potentially altering nutrient cycling, energy flow, and trophic interactions. In terms of economic impacts, the damage to feeding grounds for fish could impact regional fish stocks. Whilst fishing is restricted in some areas of the GIF Ridge hosting vulnerable marine ecosystems, including the Londsjúp trough^97^ and the Steinahóll vent field^98^, it is uncertain what impact this has to fishing operations in the wider region.

Owing to the migration and expansion of suitable habitat area, management and conservation efforts may have to consider some amphipod species as invasive, with consequent effects on regional community structures. Examples of species that might become invasive is *Oediceropsis brevicornis*, with its substantial growth in suitable habitat area and its substantial migration across a broad range of scenarios. These invasive species of amphipods could lead to a loss of biodiversity, as they may outcompete native species and alter ecosystem dynamics^99,100^.This may result in the decline of other species of amphipods in this study, or in other benthic fauna with similar ecosystem roles. Invasive species management frameworks therefore need to consider the SDMs presented in this study, especially given the important roles that amphipods play in ecosystem function.

### Study evaluation and future considerations

As with all modelling studies, this research relies on a number of assumptions and limitations that should be acknowledged when interpreting the results. One key consideration is the use of projected environmental layers from Bio-ORACLE, which, while widely used in the literature (e.g.^74,83^), introduces some uncertainty due to its spatial resolution of 0.05 degrees and decadal temporal aggregation. This resolution is appropriate for examining broader regional patterns but may limit the detection of fine-scale habitat variations and short-term environmental changes, such as marine heatwaves^101^. Nevertheless, in the absence of high-resolution in situ data across the study area, these modelled layers represent the most comprehensive option available and have been validated against quality-controlled data where possible^59,102^.

This study also presumes that the present sampling data is sufficient for a precise prediction of amphipod environmental preference, and in predicting future distributions, that this environmental preference is steady-state and unable to adapt. Whilst this study uses the best information presently available to our knowledge, future research should consider how further in situ observations can refine present-day species distributions further, and evaluate the models presented in this study for their validity. Combining these in situ observations with laboratory investigations could also help enable deeper insights into the adaptive capabilities of amphipods, leading to a greater understanding of their environmental preference.

In using open-source data in a region where information was previously limited, this study has produced new SDMs that provide a valuable baseline for understanding potential shifts in response to climate change. The SDMs show a complex and varied response of amphipods to climate forcings, with some species indicating an expansion of suitable habitat area under warming scenarios, whilst others are projected to decline in distribution. Many species show a migration of suitable habitat area toward more favourable thermohaline and biogeochemical conditions, with common patterns including a latitudinal shift towards the continental margins of Greenland or a temperature driven northward shift. The critical ecosystem roles of amphipods therefore require consideration of modelling in management and conservation efforts, with particular focus on areas where biodiversity may be irreversibly lost (e.g. GIF ridge), or where amphipods form invasive species that change ecosystem dynamics. This study highlights how species distribution modelling within the deep-sea forms an important tool for forming a baseline of present-day knowledge, and projecting future environmentally forced changes.

## Supplementary Information

Below is the link to the electronic supplementary material.

Supplementary Material 1

## Data Availability

All the data utilised in this study is open-access and can be found here https://zenodo.org/records/15396200. Amphipod occurrences are available through the database OBIS (https://obis.org/), the environmental dataset can be accessed via Bio-ORACLE (https://bio-oracle.org/) and the bathymetry data can be downloaded from GEBCO (https://www.gebco.net/data-products/gridded-bathymetry-data).
