# Supplementary material for: Species distribution modelling of benthic amphipod crustaceans in the deep North Atlantic under climate change
Source: Sci Rep. 2025 Nov 12;15:39581. doi: 10.1038/s41598-025-26442-x (PMC12612197; doi:10.1038/s41598-025-26442-x)
Supplement: Supplementary file 1 — Supplementary Material 1 [file 41598_2025_26442_MOESM1_ESM.docx]

**Species Distribution Modelling of Benthic Amphipod Crustaceans in the Deep North Atlantic Under Climate Change**

Supplementary information

**Table of contents**

Table S1: Table of Average AUC Scores per Species

Figure S1: Maps show the habitat suitability of *Ampelisca gibba* for a) present day, b) 2050–2060 and 1–1.9 SSP scenario, c) 2090–2100 and 1–1.9 SSP scenario, d) 2050–2060 and 2–4.5 SSP scenario, e) 2090–2100 and 2–4.5 SSP scenario, f) 2050–2060 and 5–8.5 SSP scenario, and g) 2090–2100 and 5–8.5 SSP. Purple indicating unsuitable habitat, yellow indicating highly suitable habitat.

Figure S2: Maps show the habitat suitability of *Harpinia laevis* for a) present day, b) 2050–2060 and 1–1.9 SSP scenario, c) 2090–2100 and 1–1.9 SSP scenario, d) 2050–2060 and 2–4.5 SSP scenario, e) 2090–2100 and 2–4.5 SSP scenario, f) 2050–2060 and 5–8.5 SSP scenario, and g) 2090–2100 and 5–8.5 SSP. Purple indicating unsuitable habitat, yellow indicating highly suitable habitat.

Figure S3: Maps show the habitat suitability of *Amphilochus manudens* for a) present day, b) 2050–2060 and 1–1.9 SSP scenario, c) 2090–2100 and 1–1.9 SSP scenario, d) 2050–2060 and 2–4.5 SSP scenario, e) 2090–2100 and 2–4.5 SSP scenario, f) 2050–2060 and 5–8.5 SSP scenario, and g) 2090–2100 and 5–8.5 SSP. Purple indicating unsuitable habitat, yellow indicating highly suitable habitat.

Figure S4: Maps show the habitat suitability of *Eriopisa elongata* for a) present day, b) 2050–2060 and 1–1.9 SSP scenario, c) 2090–2100 and 1–1.9 SSP scenario, d) 2050–2060 and 2–4.5 SSP scenario, e) 2090–2100 and 2–4.5 SSP scenario, f) 2050–2060 and 5–8.5 SSP scenario, and g) 2090–2100 and 5–8.5 SSP. Purple indicating unsuitable habitat, yellow indicating highly suitable habitat.

Figure S5: Maps show the habitat suitability of *Melphidippa borealis* for a) present day, b) 2050–2060 and 1–1.9 SSP scenario, c) 2090–2100 and 1–1.9 SSP scenario, d) 2050–2060 and 2–4.5 SSP scenario, e) 2090–2100 and 2–4.5 SSP scenario, f) 2050–2060 and 5–8.5 SSP scenario, and g) 2090–2100 and 5–8.5 SSP. Purple indicating unsuitable habitat, yellow indicating highly suitable habitat.

Figure S6: Maps show the habitat suitability of *Westwoodilla caecula* for a) present day, b) 2050–2060 and 1–1.9 SSP scenario, c) 2090–2100 and 1–1.9 SSP scenario, d) 2050–2060 and 2–4.5 SSP scenario, e) 2090–2100 and 2–4.5 SSP scenario, f) 2050–2060 and 5–8.5 SSP scenario, and g) 2090–2100 and 5–8.5 SSP. Purple indicating unsuitable habitat, yellow indicating highly suitable habitat.

Figure S7: Maps show the habitat suitability of *Harpinia crenulata* for a) present day, b) 2050–2060 and 1–1.9 SSP scenario, c) 2090–2100 and 1–1.9 SSP scenario, d) 2050–2060 and 2–4.5 SSP scenario, e) 2090–2100 and 2–4.5 SSP scenario, f) 2050–2060 and 5–8.5 SSP scenario, and g) 2090–2100 and 5–8.5 SSP. Purple indicating unsuitable habitat, yellow indicating highly suitable habitat.

Figure S8: Maps show the habitat suitability of *Laetmatophilus tuberculatus* for a) present day, b) 2050–2060 and 1–1.9 SSP scenario, c) 2090–2100 and 1–1.9 SSP scenario, d) 2050–2060 and 2–4.5 SSP scenario, e) 2090–2100 and 2–4.5 SSP scenario, f) 2050–2060 and 5–8.5 SSP scenario, and g) 2090–2100 and 5–8.5 SSP. Purple indicating unsuitable habitat, yellow indicating highly suitable habitat.

Figure S9: Maps show the habitat suitability of *Harpinia plumosa* for a) present day, b) 2050–2060 and 1–1.9 SSP scenario, c) 2090–2100 and 1–1.9 SSP scenario, d) 2050–2060 and 2–4.5 SSP scenario, e) 2090–2100 and 2–4.5 SSP scenario, f) 2050–2060 and 5–8.5 SSP scenario, and g) 2090–2100 and 5–8.5 SSP. Purple indicating unsuitable habitat, yellow indicating highly suitable habitat.

Figure S10: Maps show the habitat suitability of *Autonoe longipes* for a) present day, b) 2050–2060 and 1–1.9 SSP scenario, c) 2090–2100 and 1–1.9 SSP scenario, d) 2050–2060 and 2–4.5 SSP scenario, e) 2090–2100 and 2–4.5 SSP scenario, f) 2050–2060 and 5–8.5 SSP scenario, and g) 2090–2100 and 5–8.5 SSP. Purple indicating unsuitable habitat, yellow indicating highly suitable habitat.

Figure S11: Maps show the habitat suitability of *Byblis crassicornis* for a) present day, b) 2050–2060 and 1–1.9 SSP scenario, c) 2090–2100 and 1–1.9 SSP scenario, d) 2050–2060 and 2–4.5 SSP scenario, e) 2090–2100 and 2–4.5 SSP scenario, f) 2050–2060 and 5–8.5 SSP scenario, and g) 2090–2100 and 5–8.5 SSP. Purple indicating unsuitable habitat, yellow indicating highly suitable habitat.

Figure S12: Maps show the habitat suitability of *Harpinia antennaria* for a) present day, b) 2050–2060 and 1–1.9 SSP scenario, c) 2090–2100 and 1–1.9 SSP scenario, d) 2050–2060 and 2–4.5 SSP scenario, e) 2090–2100 and 2–4.5 SSP scenario, f) 2050–2060 and 5–8.5 SSP scenario, and g) 2090–2100 and 5–8.5 SSP. Purple indicating unsuitable habitat, yellow indicating highly suitable habitat.

Figure S13: Maps show the habitat suitability of *Tmetonyx cicada* for a) present day, b) 2050–2060 and 1–1.9 SSP scenario, c) 2090–2100 and 1–1.9 SSP scenario, d) 2050–2060 and 2–4.5 SSP scenario, e) 2090–2100 and 2–4.5 SSP scenario, f) 2050–2060 and 5–8.5 SSP scenario, and g) 2090–2100 and 5–8.5 SSP. Purple indicating unsuitable habitat, yellow indicating highly suitable habitat.

Figure S14: Maps show the habitat suitability of *Tryphosites longipes* for a) present day, b) 2050–2060 and 1–1.9 SSP scenario, c) 2090–2100 and 1–1.9 SSP scenario, d) 2050–2060 and 2–4.5 SSP scenario, e) 2090–2100 and 2–4.5 SSP scenario, f) 2050–2060 and 5–8.5 SSP scenario, and g) 2090–2100 and 5–8.5 SSP. Purple indicating unsuitable habitat, yellow indicating highly suitable habitat.

Figure S15: Maps show the habitat suitability of *Harpinia pectinata* for a) present day, b) 2050–2060 and 1–1.9 SSP scenario, c) 2090–2100 and 1–1.9 SSP scenario, d) 2050–2060 and 2–4.5 SSP scenario, e) 2090–2100 and 2–4.5 SSP scenario, f) 2050–2060 and 5–8.5 SSP scenario, and g) 2090–2100 and 5–8.5 SSP. Purple indicating unsuitable habitat, yellow indicating highly suitable habitat.

Figure S16: Maps show the habitat suitability of *Leptophoxus falcatus*

for a) present day, b) 2050–2060 and 1–1.9 SSP scenario, c) 2090–2100 and 1–1.9 SSP scenario, d) 2050–2060 and 2–4.5 SSP scenario, e) 2090–2100 and 2–4.5 SSP scenario, f) 2050–2060 and 5–8.5 SSP scenario, and g) 2090–2100 and 5–8.5 SSP. Purple indicating unsuitable habitat, yellow indicating highly suitable habitat.

Figure S17: Maps show the habitat suitability of *Nototropis nordlandicus* for a) present day, b) 2050–2060 and 1–1.9 SSP scenario, c) 2090–2100 and 1–1.9 SSP scenario, d) 2050–2060 and 2–4.5 SSP scenario, e) 2090–2100 and 2–4.5 SSP scenario, f) 2050–2060 and 5–8.5 SSP scenario, and g) 2090–2100 and 5–8.5 SSP. Purple indicating unsuitable habitat, yellow indicating highly suitable habitat.

Figure S18: Maps show the habitat suitability of *Neohela monstrosa* for a) present day, b) 2050–2060 and 1–1.9 SSP scenario, c) 2090–2100 and 1–1.9 SSP scenario, d) 2050–2060 and 2–4.5 SSP scenario, e) 2090–2100 and 2–4.5 SSP scenario, f) 2050–2060 and 5–8.5 SSP scenario, and g) 2090–2100 and 5–8.5 SSP. Purple indicating unsuitable habitat, yellow indicating highly suitable habitat.

Figure S19: Maps show the habitat suitability of *Ampelisca eschrichtii* for a) present day, b) 2050–2060 and 1–1.9 SSP scenario, c) 2090–2100 and 1–1.9 SSP scenario, d) 2050–2060 and 2–4.5 SSP scenario, e) 2090–2100 and 2–4.5 SSP scenario, f) 2050–2060 and 5–8.5 SSP scenario, and g) 2090–2100 and 5–8.5 SSP. Purple indicating unsuitable habitat, yellow indicating highly suitable habitat.

Figure S20: Maps show the habitat suitability of *Idunella aeqvicornis* for a) present day, b) 2050–2060 and 1–1.9 SSP scenario, c) 2090–2100 and 1–1.9 SSP scenario, d) 2050–2060 and 2–4.5 SSP scenario, e) 2090–2100 and 2–4.5 SSP scenario, f) 2050–2060 and 5–8.5 SSP scenario, and g) 2090–2100 and 5–8.5 SSP. Purple indicating unsuitable habitat, yellow indicating highly suitable habitat.

Figure S21: Maps show the habitat suitability of *Harpinia abyssi* for a) present day, b) 2050–2060 and 1–1.9 SSP scenario, c) 2090–2100 and 1–1.9 SSP scenario, d) 2050–2060 and 2–4.5 SSP scenario, e) 2090–2100 and 2–4.5 SSP scenario, f) 2050–2060 and 5–8.5 SSP scenario, and g) 2090–2100 and 5–8.5 SSP. Purple indicating unsuitable habitat, yellow indicating highly suitable habitat.

Figure S22: Maps show the habitat suitability of *Urothoe elegans* for a) present day, b) 2050–2060 and 1–1.9 SSP scenario, c) 2090–2100 and 1–1.9 SSP scenario, d) 2050–2060 and 2–4.5 SSP scenario, e) 2090–2100 and 2–4.5 SSP scenario, f) 2050–2060 and 5–8.5 SSP scenario, and g) 2090–2100 and 5–8.5 SSP. Purple indicating unsuitable habitat, yellow indicating highly suitable habitat.

Figure S23: Maps show the habitat suitability of *Tryphosella horingi* for a) present day, b) 2050–2060 and 1–1.9 SSP scenario, c) 2090–2100 and 1–1.9 SSP scenario, d) 2050–2060 and 2–4.5 SSP scenario, e) 2090–2100 and 2–4.5 SSP scenario, f) 2050–2060 and 5–8.5 SSP scenario, and g) 2090–2100 and 5–8.5 SSP. Purple indicating unsuitable habitat, yellow indicating highly suitable habitat.

Figure S24: Maps show the habitat suitability of *Arrhis phyllonyx* for a) present day, b) 2050–2060 and 1–1.9 SSP scenario, c) 2090–2100 and 1–1.9 SSP scenario, d) 2050–2060 and 2–4.5 SSP scenario, e) 2090–2100 and 2–4.5 SSP scenario, f) 2050–2060 and 5–8.5 SSP scenario, and g) 2090–2100 and 5–8.5 SSP. Purple indicating unsuitable habitat, yellow indicating highly suitable habitat.

Figure S25: Maps show the habitat suitability of *Harpinia propinqva* for a) present day, b) 2050–2060 and 1–1.9 SSP scenario, c) 2090–2100 and 1–1.9 SSP scenario, d) 2050–2060 and 2–4.5 SSP scenario, e) 2090–2100 and 2–4.5 SSP scenario, f) 2050–2060 and 5–8.5 SSP scenario, and g) 2090–2100 and 5–8.5 SSP. Purple indicating unsuitable habitat, yellow indicating highly suitable habitat.

Figure S26: Maps show the habitat suitability of *Unciola planipes* for a) present day, b) 2050–2060 and 1–1.9 SSP scenario, c) 2090–2100 and 1–1.9 SSP scenario, d) 2050–2060 and 2–4.5 SSP scenario, e) 2090–2100 and 2–4.5 SSP scenario, f) 2050–2060 and 5–8.5 SSP scenario, and g) 2090–2100 and 5–8.5 SSP. Purple indicating unsuitable habitat, yellow indicating highly suitable habitat.

Figure S27: Maps show the habitat suitability of *Bathymedon longimanus* for a) present day, b) 2050–2060 and 1–1.9 SSP scenario, c) 2090–2100 and 1–1.9 SSP scenario, d) 2050–2060 and 2–4.5 SSP scenario, e) 2090–2100 and 2–4.5 SSP scenario, f) 2050–2060 and 5–8.5 SSP scenario, and g) 2090–2100 and 5–8.5 SSP. Purple indicating unsuitable habitat, yellow indicating highly suitable habitat.

Figure S298: Maps show the habitat suitability of *Ampelisca macrocephala* for a) present day, b) 2050–2060 and 1–1.9 SSP scenario, c) 2090–2100 and 1–1.9 SSP scenario, d) 2050–2060 and 2–4.5 SSP scenario, e) 2090–2100 and 2–4.5 SSP scenario, f) 2050–2060 and 5–8.5 SSP scenario, and g) 2090–2100 and 5–8.5 SSP. Purple indicating unsuitable habitat, yellow indicating highly suitable habitat.

Figure S29: Maps show the habitat suitability of *Themisto abyssorum* for a) present day, b) 2050–2060 and 1–1.9 SSP scenario, c) 2090–2100 and 1–1.9 SSP scenario, d) 2050–2060 and 2–4.5 SSP scenario, e) 2090–2100 and 2–4.5 SSP scenario, f) 2050–2060 and 5–8.5 SSP scenario, and g) 2090–2100 and 5–8.5 SSP. Purple indicating unsuitable habitat, yellow indicating highly suitable habitat.

Figure S30: Maps show the habitat suitability of *Hippomedon propinqvus* for a) present day, b) 2050–2060 and 1–1.9 SSP scenario, c) 2090–2100 and 1–1.9 SSP scenario, d) 2050–2060 and 2–4.5 SSP scenario, e) 2090–2100 and 2–4.5 SSP scenario, f) 2050–2060 and 5–8.5 SSP scenario, and g) 2090–2100 and 5–8.5 SSP. Purple indicating unsuitable habitat, yellow indicating highly suitable habitat.

Figure S31: Maps show the habitat suitability of *Liljeborgia fissicornis* for a) present day, b) 2050–2060 and 1–1.9 SSP scenario, c) 2090–2100 and 1–1.9 SSP scenario, d) 2050–2060 and 2–4.5 SSP scenario, e) 2090–2100 and 2–4.5 SSP scenario, f) 2050–2060 and 5–8.5 SSP scenario, and g) 2090–2100 and 5–8.5 SSP. Purple indicating unsuitable habitat, yellow indicating highly suitable habitat.

Figure S32: Maps show the habitat suitability of *Syrrhoe crenulata* for a) present day, b) 2050–2060 and 1–1.9 SSP scenario, c) 2090–2100 and 1–1.9 SSP scenario, d) 2050–2060 and 2–4.5 SSP scenario, e) 2090–2100 and 2–4.5 SSP scenario, f) 2050–2060 and 5–8.5 SSP scenario, and g) 2090–2100 and 5–8.5 SSP. Purple indicating unsuitable habitat, yellow indicating highly suitable habitat.

Figure S33: Maps show the habitat suitability of *Hippomedon denticulatus* for a) present day, b) 2050–2060 and 1–1.9 SSP scenario, c) 2090–2100 and 1–1.9 SSP scenario, d) 2050–2060 and 2–4.5 SSP scenario, e) 2090–2100 and 2–4.5 SSP scenario, f) 2050–2060 and 5–8.5 SSP scenario, and g) 2090–2100 and 5–8.5 SSP. Purple indicating unsuitable habitat, yellow indicating highly suitable habitat.

Figure S34: Maps show the habitat suitability of *Ampelisca aequicornis* for a) present day, b) 2050–2060 and 1–1.9 SSP scenario, c) 2090–2100 and 1–1.9 SSP scenario, d) 2050–2060 and 2–4.5 SSP scenario, e) 2090–2100 and 2–4.5 SSP scenario, f) 2050–2060 and 5–8.5 SSP scenario, and g) 2090–2100 and 5–8.5 SSP. Purple indicating unsuitable habitat, yellow indicating highly suitable habitat.

Figure S35: Maps show the habitat suitability of *Tmetonyx similis* for a) present day, b) 2050–2060 and 1–1.9 SSP scenario, c) 2090–2100 and 1–1.9 SSP scenario, d) 2050–2060 and 2–4.5 SSP scenario, e) 2090–2100 and 2–4.5 SSP scenario, f) 2050–2060 and 5–8.5 SSP scenario, and g) 2090–2100 and 5–8.5 SSP. Purple indicating unsuitable habitat, yellow indicating highly suitable habitat.

Figure S36: Maps show the habitat suitability of *Aceroides (Aceroides) latipes* for a) present day, b) 2050–2060 and 1–1.9 SSP scenario, c) 2090–2100 and 1–1.9 SSP scenario, d) 2050–2060 and 2–4.5 SSP scenario, e) 2090–2100 and 2–4.5 SSP scenario, f) 2050–2060 and 5–8.5 SSP scenario, and g) 2090–2100 and 5–8.5 SSP. Purple indicating unsuitable habitat, yellow indicating highly suitable habitat.

Figure S37: Maps show the habitat suitability of *Harpinia mucronata* for a) present day, b) 2050–2060 and 1–1.9 SSP scenario, c) 2090–2100 and 1–1.9 SSP scenario, d) 2050–2060 and 2–4.5 SSP scenario, e) 2090–2100 and 2–4.5 SSP scenario, f) 2050–2060 and 5–8.5 SSP scenario, and g) 2090–2100 and 5–8.5 SSP. Purple indicating unsuitable habitat, yellow indicating highly suitable habitat.

Figure S38: Maps show the habitat suitability of *Haploops tubicola* for a) present day, b) 2050–2060 and 1–1.9 SSP scenario, c) 2090–2100 and 1–1.9 SSP scenario, d) 2050–2060 and 2–4.5 SSP scenario, e) 2090–2100 and 2–4.5 SSP scenario, f) 2050–2060 and 5–8.5 SSP scenario, and g) 2090–2100 and 5–8.5 SSP. Purple indicating unsuitable habitat, yellow indicating highly suitable habitat.

Figure S39: Maps show the habitat suitability of *Unciola leucopis* for a) present day, b) 2050–2060 and 1–1.9 SSP scenario, c) 2090–2100 and 1–1.9 SSP scenario, d) 2050–2060 and 2–4.5 SSP scenario, e) 2090–2100 and 2–4.5 SSP scenario, f) 2050–2060 and 5–8.5 SSP scenario, and g) 2090–2100 and 5–8.5 SSP. Purple indicating unsuitable habitat, yellow indicating highly suitable habitat.

Figure S40: Maps show the habitat suitability of *Haploops setosa* for a) present day, b) 2050–2060 and 1–1.9 SSP scenario, c) 2090–2100 and 1–1.9 SSP scenario, d) 2050–2060 and 2–4.5 SSP scenario, e) 2090–2100 and 2–4.5 SSP scenario, f) 2050–2060 and 5–8.5 SSP scenario, and g) 2090–2100 and 5–8.5 SSP. Purple indicating unsuitable habitat, yellow indicating highly suitable habitat.

Figure S41: Maps show the habitat suitability of *Bruzelia typica* for a) present day, b) 2050–2060 and 1–1.9 SSP scenario, c) 2090–2100 and 1–1.9 SSP scenario, d) 2050–2060 and 2–4.5 SSP scenario, e) 2090–2100 and 2–4.5 SSP scenario, f) 2050–2060 and 5–8.5 SSP scenario, and g) 2090–2100 and 5–8.5 SSP. Purple indicating unsuitable habitat, yellow indicating highly suitable habitat.

Figure S42: Maps show the habitat suitability of *Xenodice frauenfeldti* for a) present day, b) 2050–2060 and 1–1.9 SSP scenario, c) 2090–2100 and 1–1.9 SSP scenario, d) 2050–2060 and 2–4.5 SSP scenario, e) 2090–2100 and 2–4.5 SSP scenario, f) 2050–2060 and 5–8.5 SSP scenario, and g) 2090–2100 and 5–8.5 SSP. Purple indicating unsuitable habitat, yellow indicating highly suitable habitat.

Figure S43: Maps show the habitat suitability of *Ischyrocerus megacheir* for a) present day, b) 2050–2060 and 1–1.9 SSP scenario, c) 2090–2100 and 1–1.9 SSP scenario, d) 2050–2060 and 2–4.5 SSP scenario, e) 2090–2100 and 2–4.5 SSP scenario, f) 2050–2060 and 5–8.5 SSP scenario, and g) 2090–2100 and 5–8.5 SSP. Purple indicating unsuitable habitat, yellow indicating highly suitable habitat.

Figure S44: Maps show the habitat suitability of *Nicippe tumida* for a) present day, b) 2050–2060 and 1–1.9 SSP scenario, c) 2090–2100 and 1–1.9 SSP scenario, d) 2050–2060 and 2–4.5 SSP scenario, e) 2090–2100 and 2–4.5 SSP scenario, f) 2050–2060 and 5–8.5 SSP scenario, and g) 2090–2100 and 5–8.5 SSP. Purple indicating unsuitable habitat, yellow indicating highly suitable habitat.

Figure S45: Maps show the habitat suitability of *Liljeborgia pallida* for a) present day, b) 2050–2060 and 1–1.9 SSP scenario, c) 2090–2100 and 1–1.9 SSP scenario, d) 2050–2060 and 2–4.5 SSP scenario, e) 2090–2100 and 2–4.5 SSP scenario, f) 2050–2060 and 5–8.5 SSP scenario, and g) 2090–2100 and 5–8.5 SSP. Purple indicating unsuitable habitat, yellow indicating highly suitable habitat.

Figure S46: Maps show the habitat suitability of *Bathymedon saussurei* for a) present day, b) 2050–2060 and 1–1.9 SSP scenario, c) 2090–2100 and 1–1.9 SSP scenario, d) 2050–2060 and 2–4.5 SSP scenario, e) 2090–2100 and 2–4.5 SSP scenario, f) 2050–2060 and 5–8.5 SSP scenario, and g) 2090–2100 and 5–8.5 SSP. Purple indicating unsuitable habitat, yellow indicating highly suitable habitat.

Figure S47: Maps show the habitat suitability of *Autonoe megacheir* for a) present day, b) 2050–2060 and 1–1.9 SSP scenario, c) 2090–2100 and 1–1.9 SSP scenario, d) 2050–2060 and 2–4.5 SSP scenario, e) 2090–2100 and 2–4.5 SSP scenario, f) 2050–2060 and 5–8.5 SSP scenario, and g) 2090–2100 and 5–8.5 SSP. Purple indicating unsuitable habitat, yellow indicating highly suitable habitat.

Figure S48: Maps show the habitat suitability of *Halice abyssi* for a) present day, b) 2050–2060 and 1–1.9 SSP scenario, c) 2090–2100 and 1–1.9 SSP scenario, d) 2050–2060 and 2–4.5 SSP scenario, e) 2090–2100 and 2–4.5 SSP scenario, f) 2050–2060 and 5–8.5 SSP scenario, and g) 2090–2100 and 5–8.5 SSP. Purple indicating unsuitable habitat, yellow indicating highly suitable habitat.

Figure S49: Maps show the habitat suitability of *Ampelisca odontoplax* for a) present day, b) 2050–2060 and 1–1.9 SSP scenario, c) 2090–2100 and 1–1.9 SSP scenario, d) 2050–2060 and 2–4.5 SSP scenario, e) 2090–2100 and 2–4.5 SSP scenario, f) 2050–2060 and 5–8.5 SSP scenario, and g) 2090–2100 and 5–8.5 SSP. Purple indicating unsuitable habitat, yellow indicating highly suitable habitat.

Figure S50: Maps show the habitat suitability of *Medicorophium affine* for a) present day, b) 2050–2060 and 1–1.9 SSP scenario, c) 2090–2100 and 1–1.9 SSP scenario, d) 2050–2060 and 2–4.5 SSP scenario, e) 2090–2100 and 2–4.5 SSP scenario, f) 2050–2060 and 5–8.5 SSP scenario, and g) 2090–2100 and 5–8.5 SSP. Purple indicating unsuitable habitat, yellow indicating highly suitable habitat.

Figure S51: Maps show the habitat suitability of *Ampelisca pusilla* for a) present day, b) 2050–2060 and 1–1.9 SSP scenario, c) 2090–2100 and 1–1.9 SSP scenario, d) 2050–2060 and 2–4.5 SSP scenario, e) 2090–2100 and 2–4.5 SSP scenario, f) 2050–2060 and 5–8.5 SSP scenario, and g) 2090–2100 and 5–8.5 SSP. Purple indicating unsuitable habitat, yellow indicating highly suitable habitat.

Figure S52: Maps show the habitat suitability of *Oediceropsis brevicornis* for a) present day, b) 2050–2060 and 1–1.9 SSP scenario, c) 2090–2100 and 1–1.9 SSP scenario, d) 2050–2060 and 2–4.5 SSP scenario, e) 2090–2100 and 2–4.5 SSP scenario, f) 2050–2060 and 5–8.5 SSP scenario, and g) 2090–2100 and 5–8.5 SSP. Purple indicating unsuitable habitat, yellow indicating highly suitable habitat.

Figure S53: Maps show the habitat suitability of *Paraphoxus oculatus* for a) present day, b) 2050–2060 and 1–1.9 SSP scenario, c) 2090–2100 and 1–1.9 SSP scenario, d) 2050–2060 and 2–4.5 SSP scenario, e) 2090–2100 and 2–4.5 SSP scenario, f) 2050–2060 and 5–8.5 SSP scenario, and g) 2090–2100 and 5–8.5 SSP. Purple indicating unsuitable habitat, yellow indicating highly suitable habitat.

Figure S54: Maps show the habitat suitability of *Lysianassa plumosa* for a) present day, b) 2050–2060 and 1–1.9 SSP scenario, c) 2090–2100 and 1–1.9 SSP scenario, d) 2050–2060 and 2–4.5 SSP scenario, e) 2090–2100 and 2–4.5 SSP scenario, f) 2050–2060 and 5–8.5 SSP scenario, and g) 2090–2100 and 5–8.5 SSP. Purple indicating unsuitable habitat, yellow indicating highly suitable habitat.

Figure S55: Maps show the habitat suitability of *Byblis gaimardii* for a) present day, b) 2050–2060 and 1–1.9 SSP scenario, c) 2090–2100 and 1–1.9 SSP scenario, d) 2050–2060 and 2–4.5 SSP scenario, e) 2090–2100 and 2–4.5 SSP scenario, f) 2050–2060 and 5–8.5 SSP scenario, and g) 2090–2100 and 5–8.5 SSP. Purple indicating unsuitable habitat, yellow indicating highly suitable habitat.

References

Table S1. Table of Average AUC Scores per Species

| **Species** | **Avg AUC** |
| --- | --- |
| *Ampelisca gibba* | 0.93 |
| *Harpinia laevis* | 0.88 |
| *Amphilochus manudens* | 0.76 |
| *Eriopisa elongata* | 0.85 |
| *Melphidippa borealis* | 0.79 |
| *Westwoodilla caecula* | 0.86 |
| *Harpinia crenulata* | 0.81 |
| *Laetmatophilus tuberculatus* | 0.82 |
| *Harpinia plumosa* | 0.96 |
| *Autonoe longipes* | 0.88 |
| *Byblis crassicornis* | 0.88 |
| *Harpinia antennaria* | 0.85 |
| *Tmetonyx cicada* | 0.79 |
| *Tryphosites longipes* | 0.9 |
| *Harpinia pectinata* | 0.87 |
| *Leptophoxus falcatus* | 0.83 |
| *Nototropis nordlandicus* | 0.81 |
| *Neohela monstrosa* | 0.8 |
| *Ampelisca eschrichtii* | 0.87 |
| *Idunella aeqvicornis* | 0.89 |
| *Harpinia abyssi* | 0.99 |
| *Urothoe elegans* | 0.9 |
| *Tryphosella horingi* | 0.74 |
| *Arrhis phyllonyx* | 0.88 |
| *Harpinia propinqva* | 0.92 |
| *Unciola planipes* | 0.86 |
| *Bathymedon longimanus* | 0.82 |
| *Ampelisca macrocephala* | 0.88 |
| *Themisto abyssorum* | 0.84 |
| *Hippomedon propinqvus* | 0.78 |
| *Liljeborgia fissicornis* | 0.72 |
| *Syrrhoe crenulata* | 0.82 |
| *Hippomedon denticulatus* | 0.86 |
| *Ampelisca aequicornis* | 0.82 |
| *Tmetonyx similis* | 0.74 |
| *Aceroides (Aceroides) latipes* | 0.97 |
| *Harpinia mucronata* | 0.89 |
| *Haploops tubicola* | 0.88 |
| *Unciola leucopis* | 0.89 |
| *Haploops setosa* | 0.78 |
| *Bruzelia typica* | 0.88 |
| *Xenodice frauenfeldti* | 0.86 |
| *Ischyrocerus megacheir* | 0.78 |
| *Nicippe tumida* | 0.84 |
| *Liljeborgia pallida* | 0.87 |
| *Bathymedon saussurei* | 0.77 |
| *Autonoe megacheir* | 0.9 |
| *Halice abyssi* | 0.76 |
| *Ampelisca odontoplax* | 0.87 |
| *Medicorophium affine* | 0.91 |
| *Ampelisca pusilla* | 0.91 |
| *Oediceropsis brevicornis* | 0.88 |
| *Paraphoxus oculatus* | 0.82 |
| *Lysianassa plumosa* | 0.92 |
| *Byblis gaimardii* | 0.89 |


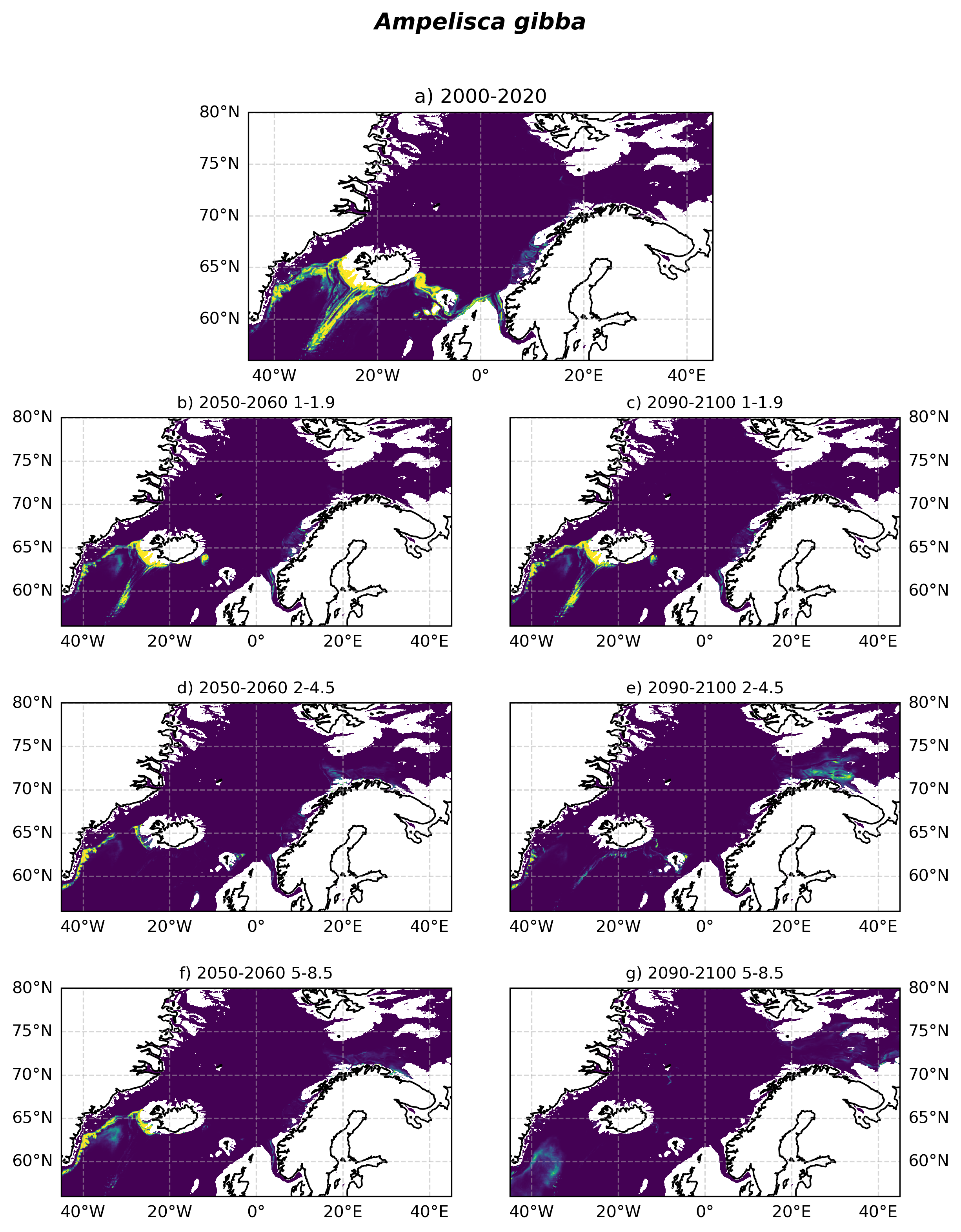


Figure S1: Maps show the habitat suitability of *Ampelisca gibba* for a) present day, b) 2050–2060 and 1–1.9 SSP scenario, c) 2090–2100 and 1–1.9 SSP scenario, d) 2050–2060 and 2–4.5 SSP scenario, e) 2090–2100 and 2–4.5 SSP scenario, f) 2050–2060 and 5–8.5 SSP scenario, and g) 2090–2100 and 5–8.5 SSP. Purple indicating unsuitable habitat, yellow indicating highly suitable habitat.


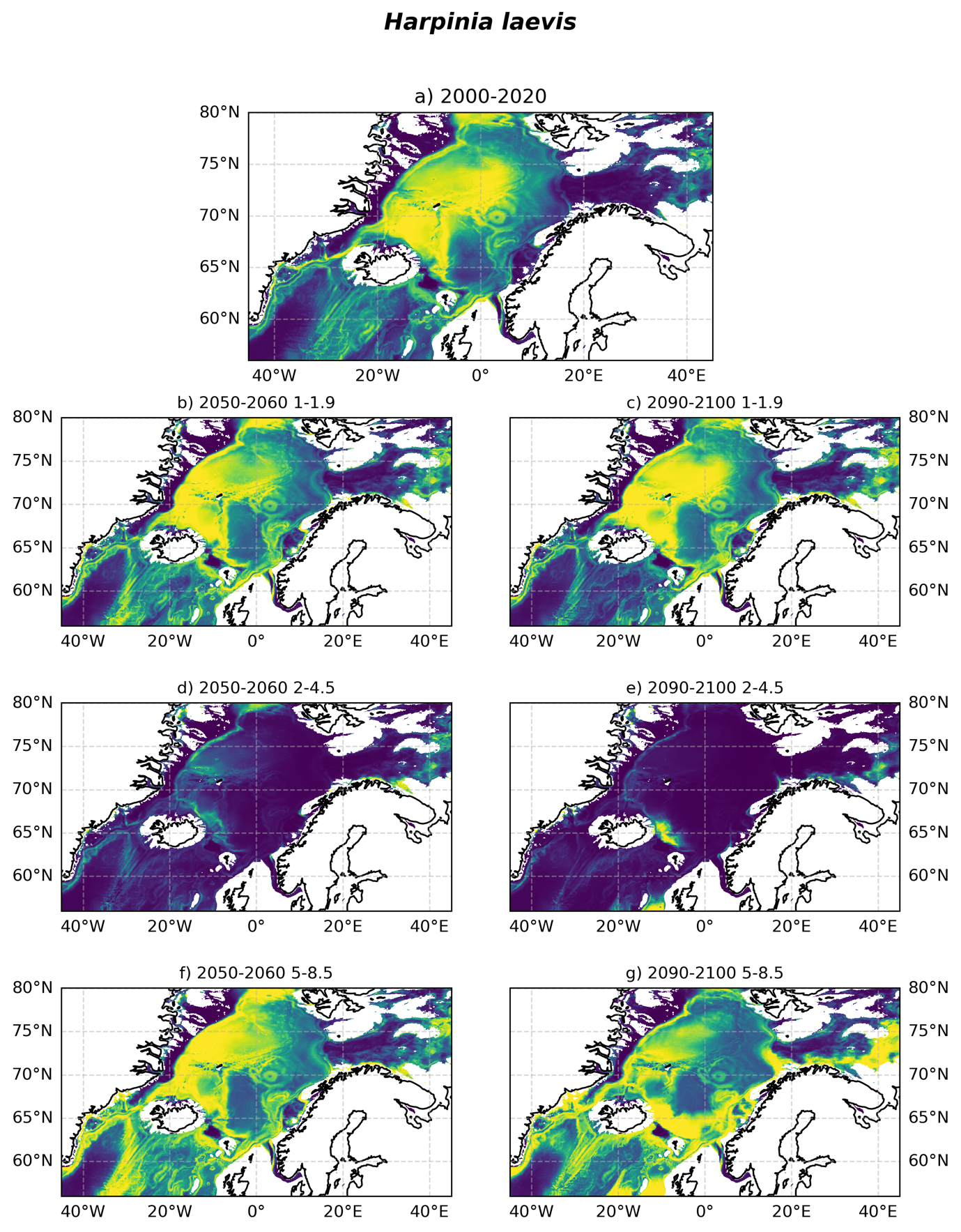


Figure S2: Maps show the habitat suitability of *Harpinia laevis* for a) present day, b) 2050–2060 and 1–1.9 SSP scenario, c) 2090–2100 and 1–1.9 SSP scenario, d) 2050–2060 and 2–4.5 SSP scenario, e) 2090–2100 and 2–4.5 SSP scenario, f) 2050–2060 and 5–8.5 SSP scenario, and g) 2090–2100 and 5–8.5 SSP. Purple indicating unsuitable habitat, yellow indicating highly suitable habitat.


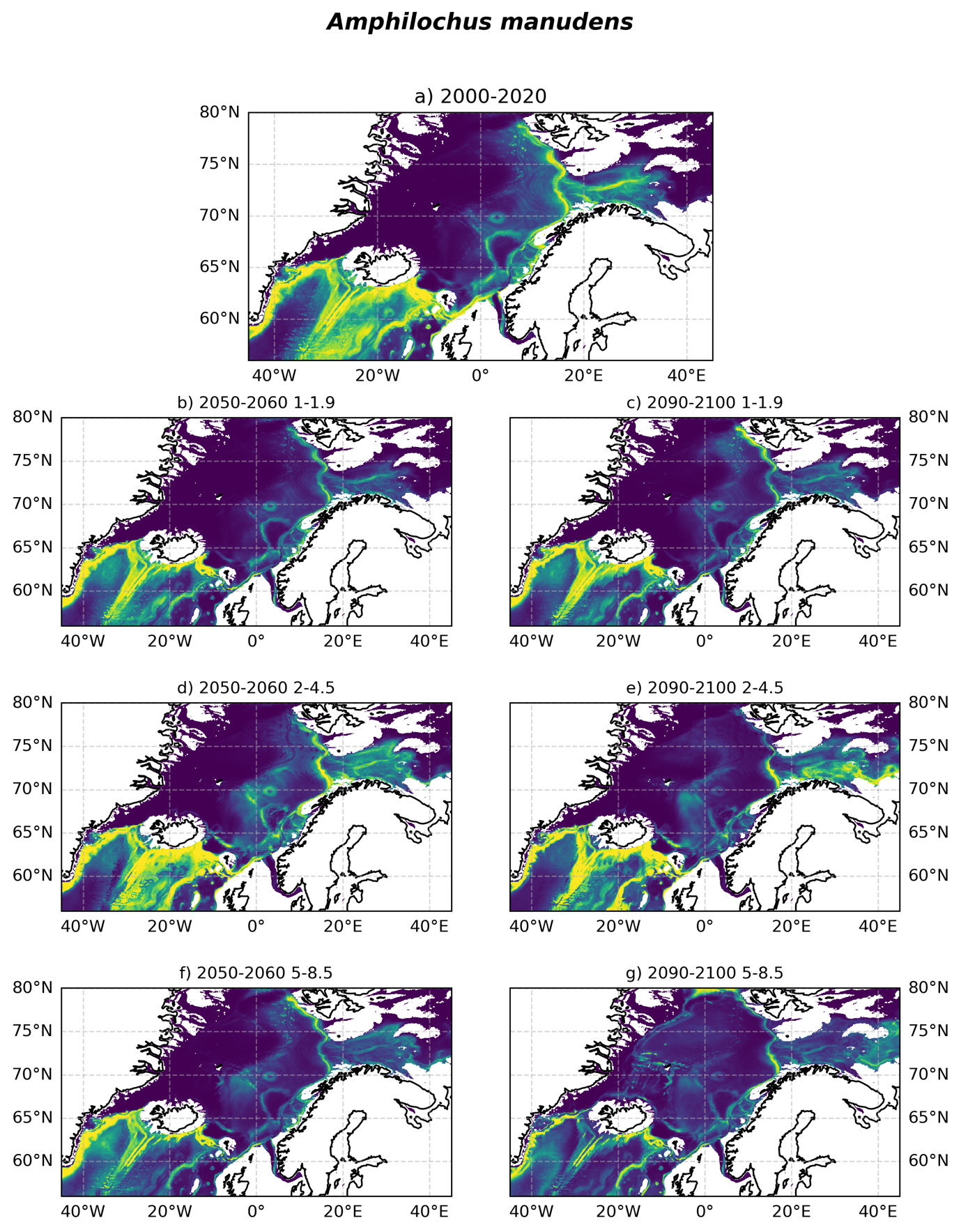


Figure S3: Maps show the habitat suitability of *Amphilochus manudens* for a) present day, b) 2050–2060 and 1–1.9 SSP scenario, c) 2090–2100 and 1–1.9 SSP scenario, d) 2050–2060 and 2–4.5 SSP scenario, e) 2090–2100 and 2–4.5 SSP scenario, f) 2050–2060 and 5–8.5 SSP scenario, and g) 2090–2100 and 5–8.5 SSP. Purple indicating unsuitable habitat, yellow indicating highly suitable habitat.


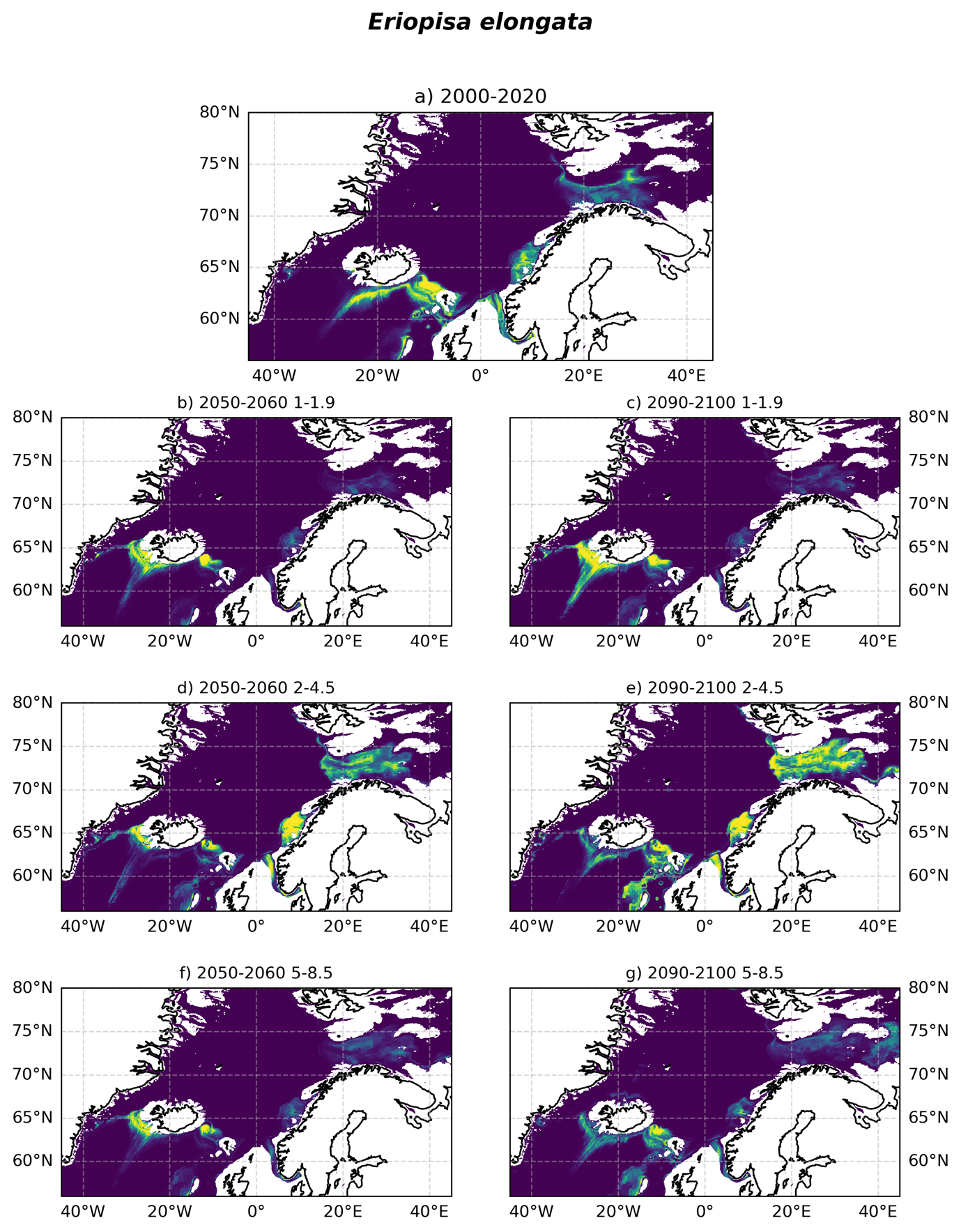


Figure S4: Maps show the habitat suitability of *Eriopisa elongata* for a) present day, b) 2050–2060 and 1–1.9 SSP scenario, c) 2090–2100 and 1–1.9 SSP scenario, d) 2050–2060 and 2–4.5 SSP scenario, e) 2090–2100 and 2–4.5 SSP scenario, f) 2050–2060 and 5–8.5 SSP scenario, and g) 2090–2100 and 5–8.5 SSP. Purple indicating unsuitable habitat, yellow indicating highly suitable habitat.


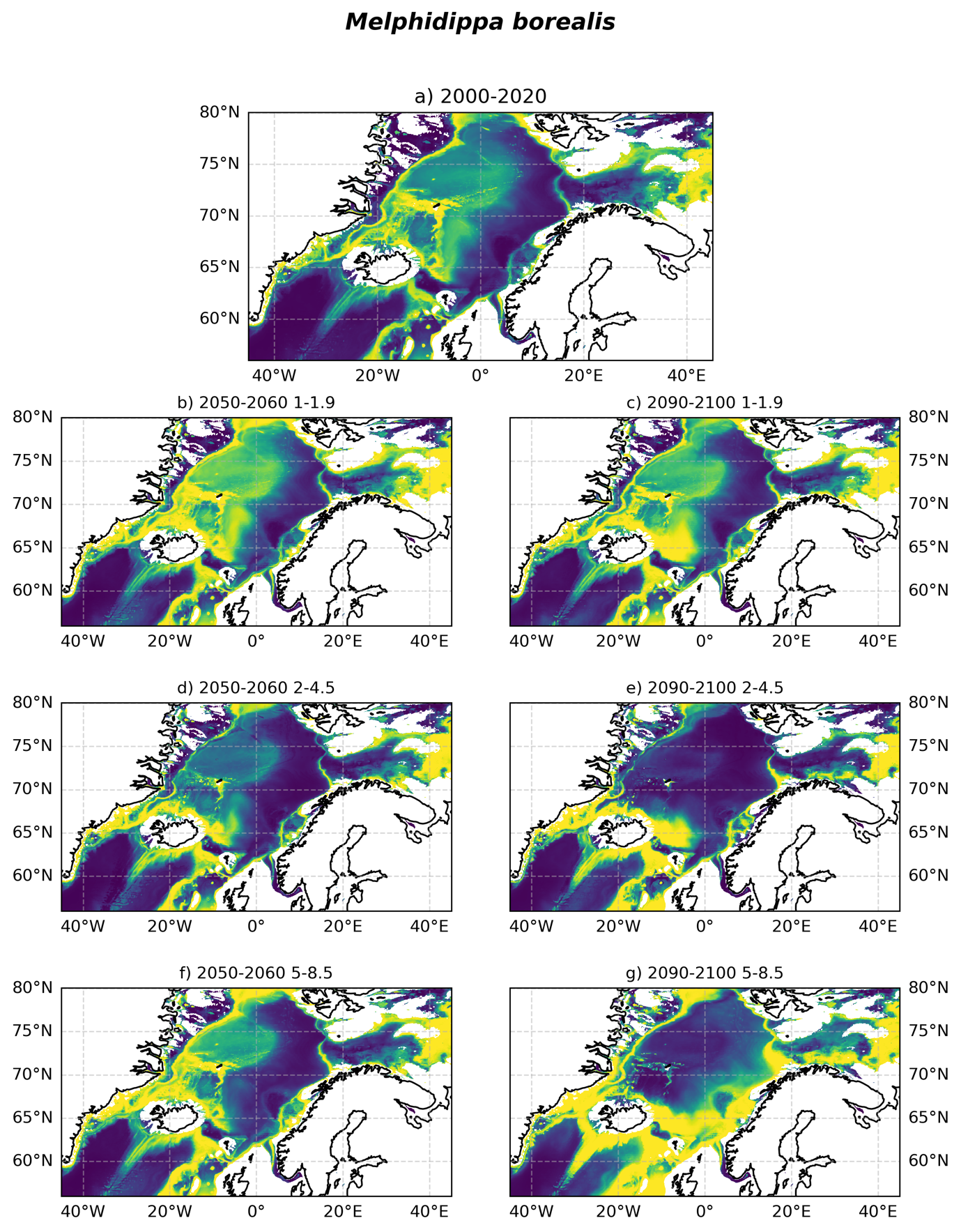


Figure S5: Maps show the habitat suitability of *Melphidippa borealis* for a) present day, b) 2050–2060 and 1–1.9 SSP scenario, c) 2090–2100 and 1–1.9 SSP scenario, d) 2050–2060 and 2–4.5 SSP scenario, e) 2090–2100 and 2–4.5 SSP scenario, f) 2050–2060 and 5–8.5 SSP scenario, and g) 2090–2100 and 5–8.5 SSP. Purple indicating unsuitable habitat, yellow indicating highly suitable habitat.


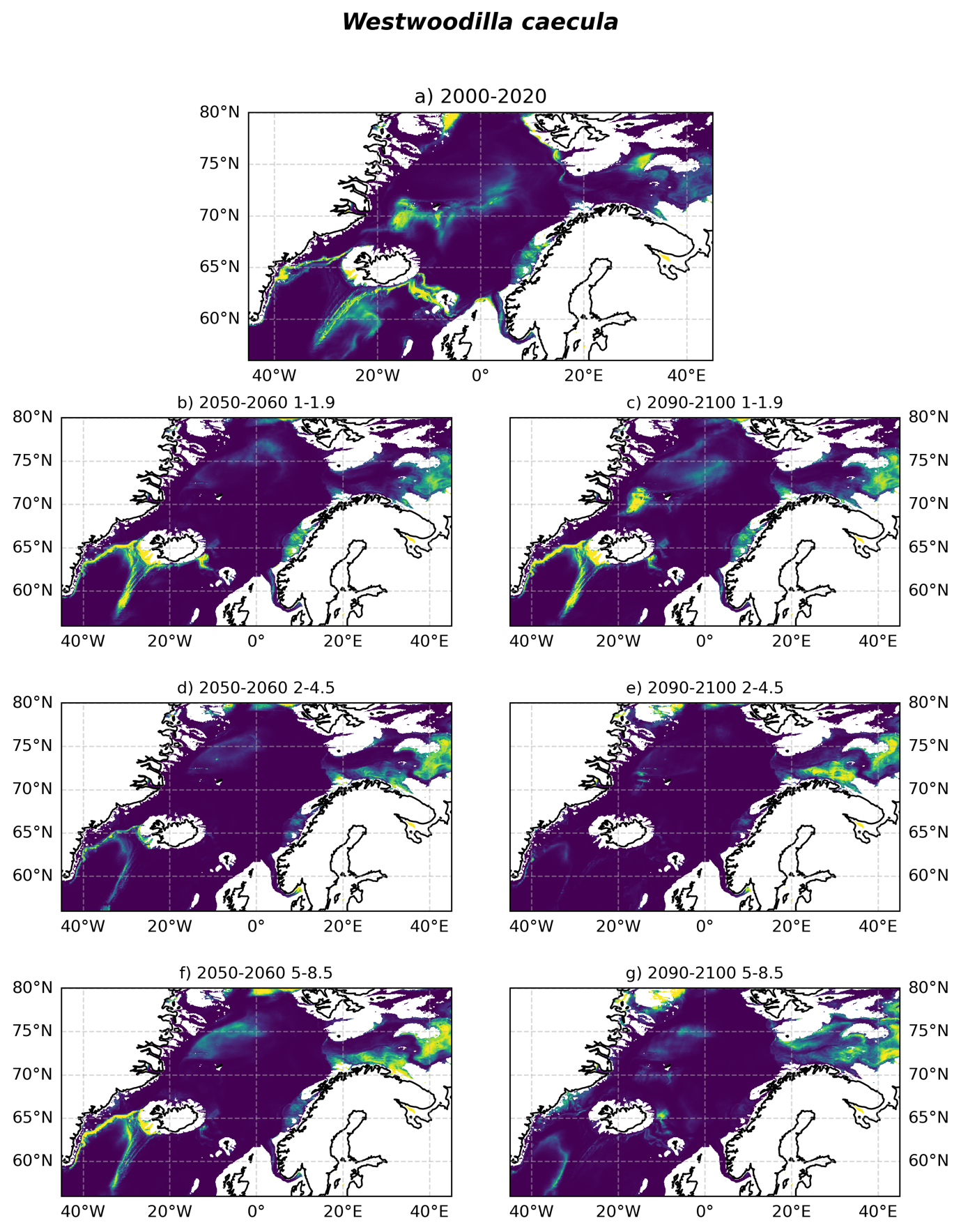


Figure S6: Maps show the habitat suitability of *Westwoodilla caecula* for a) present day, b) 2050–2060 and 1–1.9 SSP scenario, c) 2090–2100 and 1–1.9 SSP scenario, d) 2050–2060 and 2–4.5 SSP scenario, e) 2090–2100 and 2–4.5 SSP scenario, f) 2050–2060 and 5–8.5 SSP scenario, and g) 2090–2100 and 5–8.5 SSP. Purple indicating unsuitable habitat, yellow indicating highly suitable habitat.


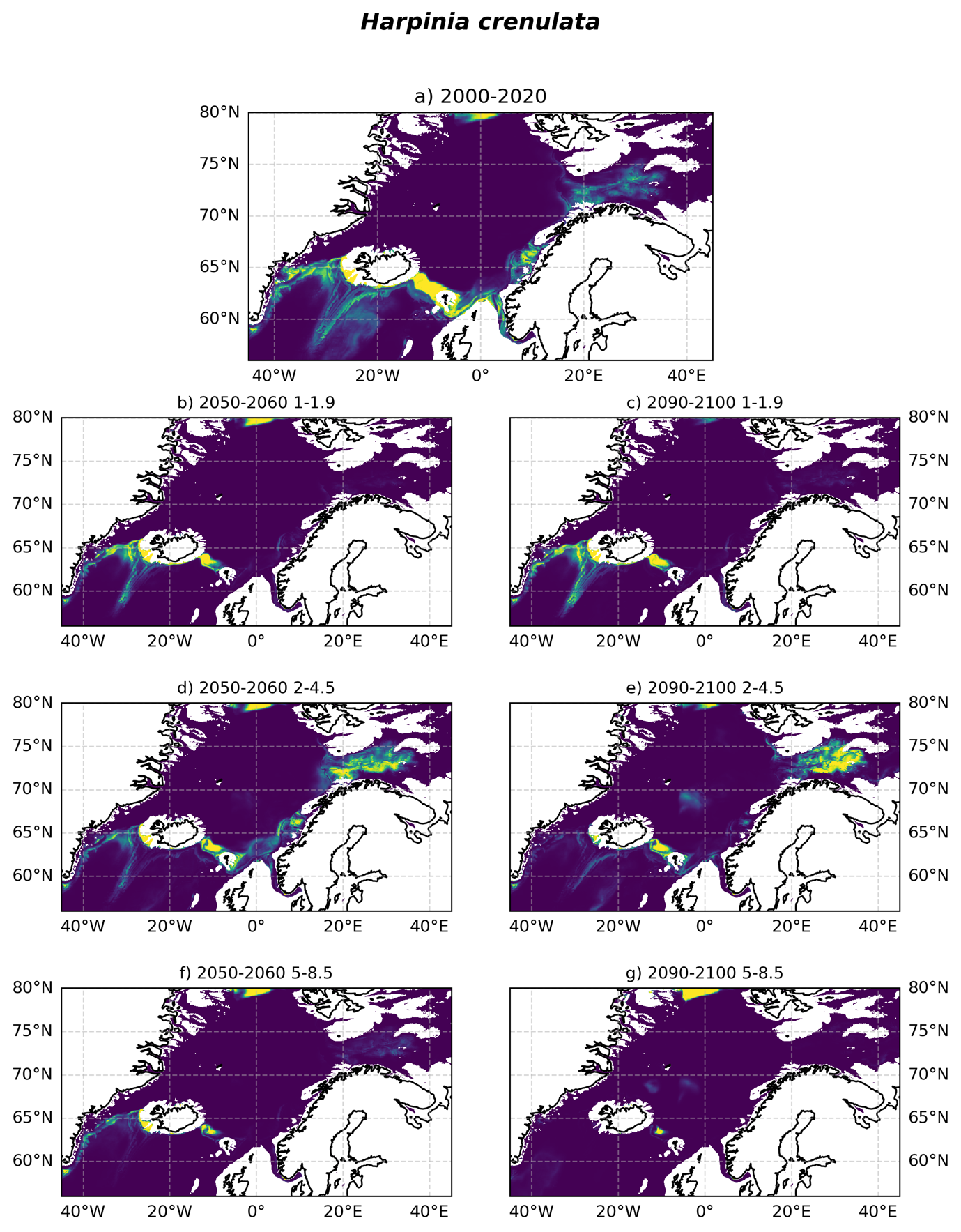


Figure S7: Maps show the habitat suitability of *Harpinia crenulata* for a) present day, b) 2050–2060 and 1–1.9 SSP scenario, c) 2090–2100 and 1–1.9 SSP scenario, d) 2050–2060 and 2–4.5 SSP scenario, e) 2090–2100 and 2–4.5 SSP scenario, f) 2050–2060 and 5–8.5 SSP scenario, and g) 2090–2100 and 5–8.5 SSP. Purple indicating unsuitable habitat, yellow indicating highly suitable habitat.


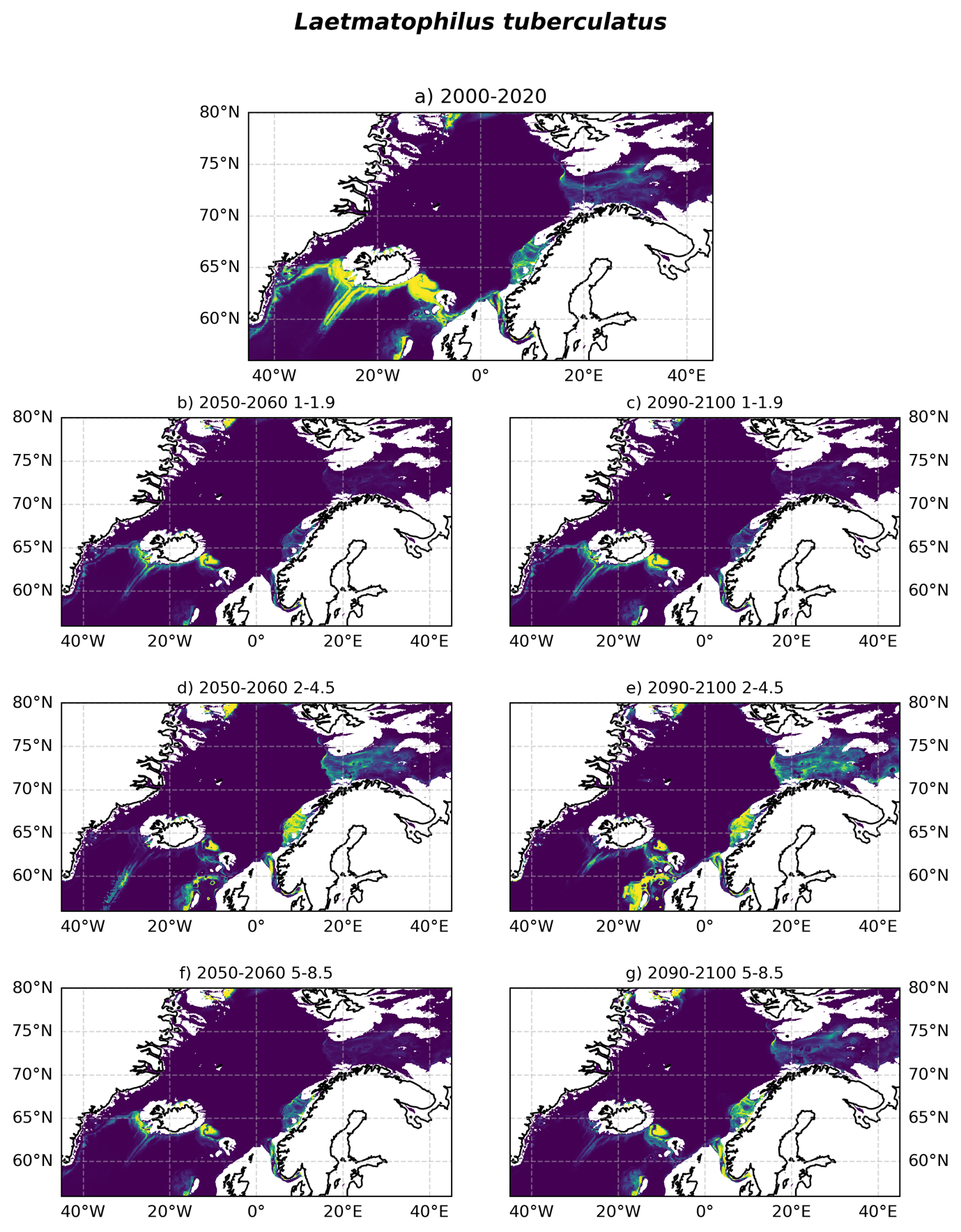


Figure S8: Maps show the habitat suitability of *Laetmatophilus tuberculatus* for a) present day, b) 2050–2060 and 1–1.9 SSP scenario, c) 2090–2100 and 1–1.9 SSP scenario, d) 2050–2060 and 2–4.5 SSP scenario, e) 2090–2100 and 2–4.5 SSP scenario, f) 2050–2060 and 5–8.5 SSP scenario, and g) 2090–2100 and 5–8.5 SSP. Purple indicating unsuitable habitat, yellow indicating highly suitable habitat.


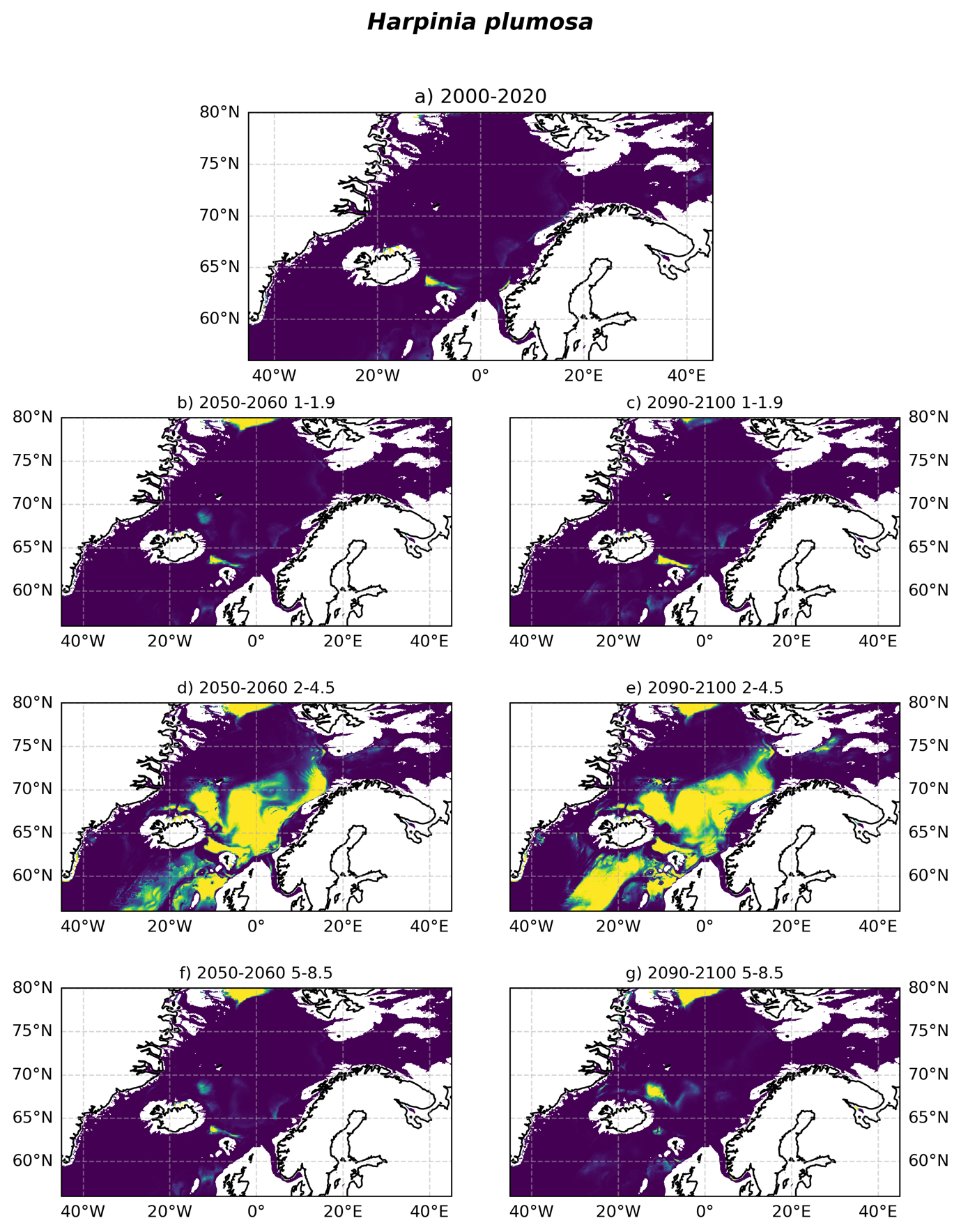


Figure S9: Maps show the habitat suitability of *Harpinia plumosa* for a) present day, b) 2050–2060 and 1–1.9 SSP scenario, c) 2090–2100 and 1–1.9 SSP scenario, d) 2050–2060 and 2–4.5 SSP scenario, e) 2090–2100 and 2–4.5 SSP scenario, f) 2050–2060 and 5–8.5 SSP scenario, and g) 2090–2100 and 5–8.5 SSP. Purple indicating unsuitable habitat, yellow indicating highly suitable habitat.


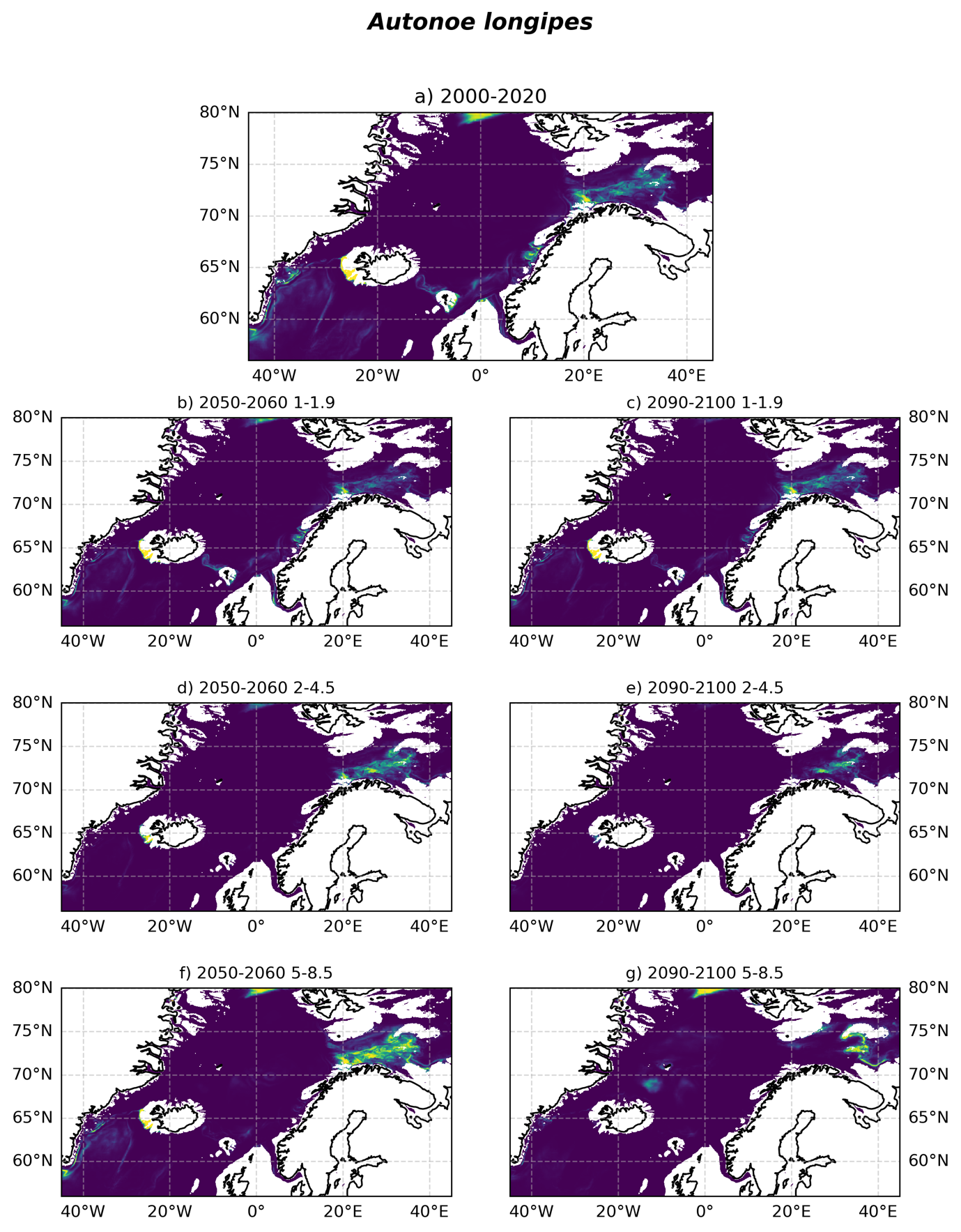


Figure S10: Maps show the habitat suitability of *Autonoe longipes* for a) present day, b) 2050–2060 and 1–1.9 SSP scenario, c) 2090–2100 and 1–1.9 SSP scenario, d) 2050–2060 and 2–4.5 SSP scenario, e) 2090–2100 and 2–4.5 SSP scenario, f) 2050–2060 and 5–8.5 SSP scenario, and g) 2090–2100 and 5–8.5 SSP. Purple indicating unsuitable habitat, yellow indicating highly suitable habitat.


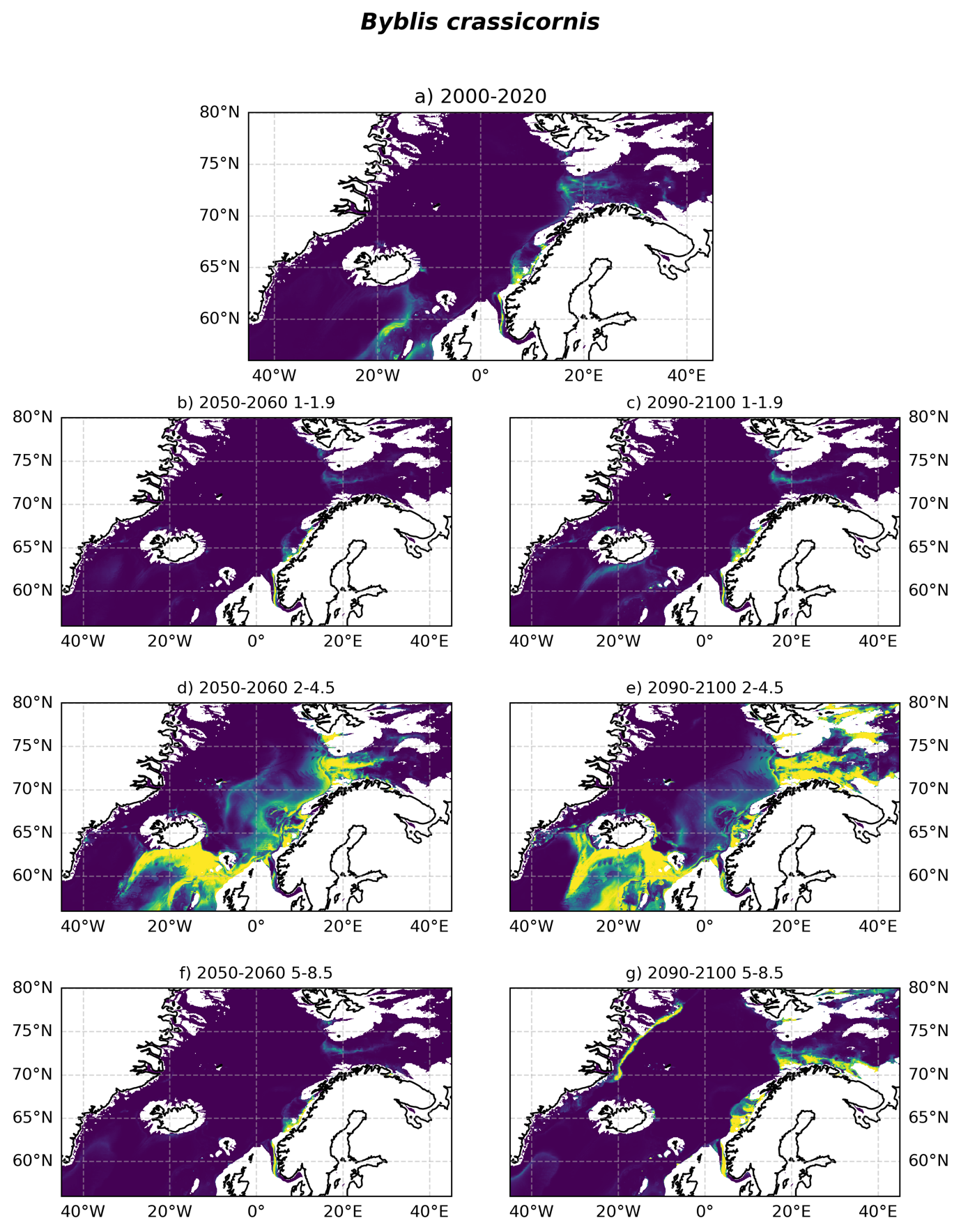


Figure S11: Maps show the habitat suitability of *Byblis crassicornis* for a) present day, b) 2050–2060 and 1–1.9 SSP scenario, c) 2090–2100 and 1–1.9 SSP scenario, d) 2050–2060 and 2–4.5 SSP scenario, e) 2090–2100 and 2–4.5 SSP scenario, f) 2050–2060 and 5–8.5 SSP scenario, and g) 2090–2100 and 5–8.5 SSP. Purple indicating unsuitable habitat, yellow indicating highly suitable habitat.


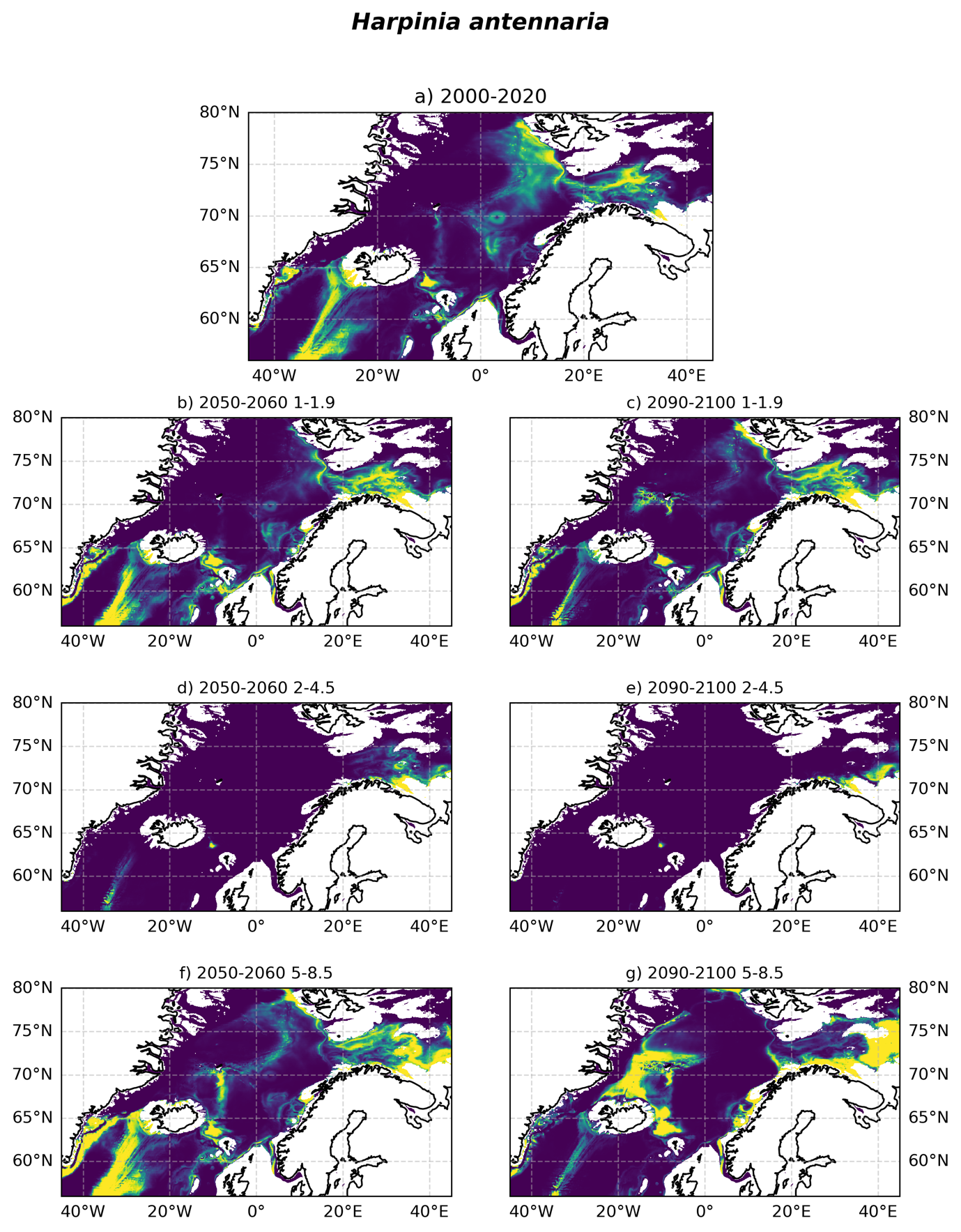


Figure S12: Maps show the habitat suitability of *Harpinia antennaria* for a) present day, b) 2050–2060 and 1–1.9 SSP scenario, c) 2090–2100 and 1–1.9 SSP scenario, d) 2050–2060 and 2–4.5 SSP scenario, e) 2090–2100 and 2–4.5 SSP scenario, f) 2050–2060 and 5–8.5 SSP scenario, and g) 2090–2100 and 5–8.5 SSP. Purple indicating unsuitable habitat, yellow indicating highly suitable habitat.


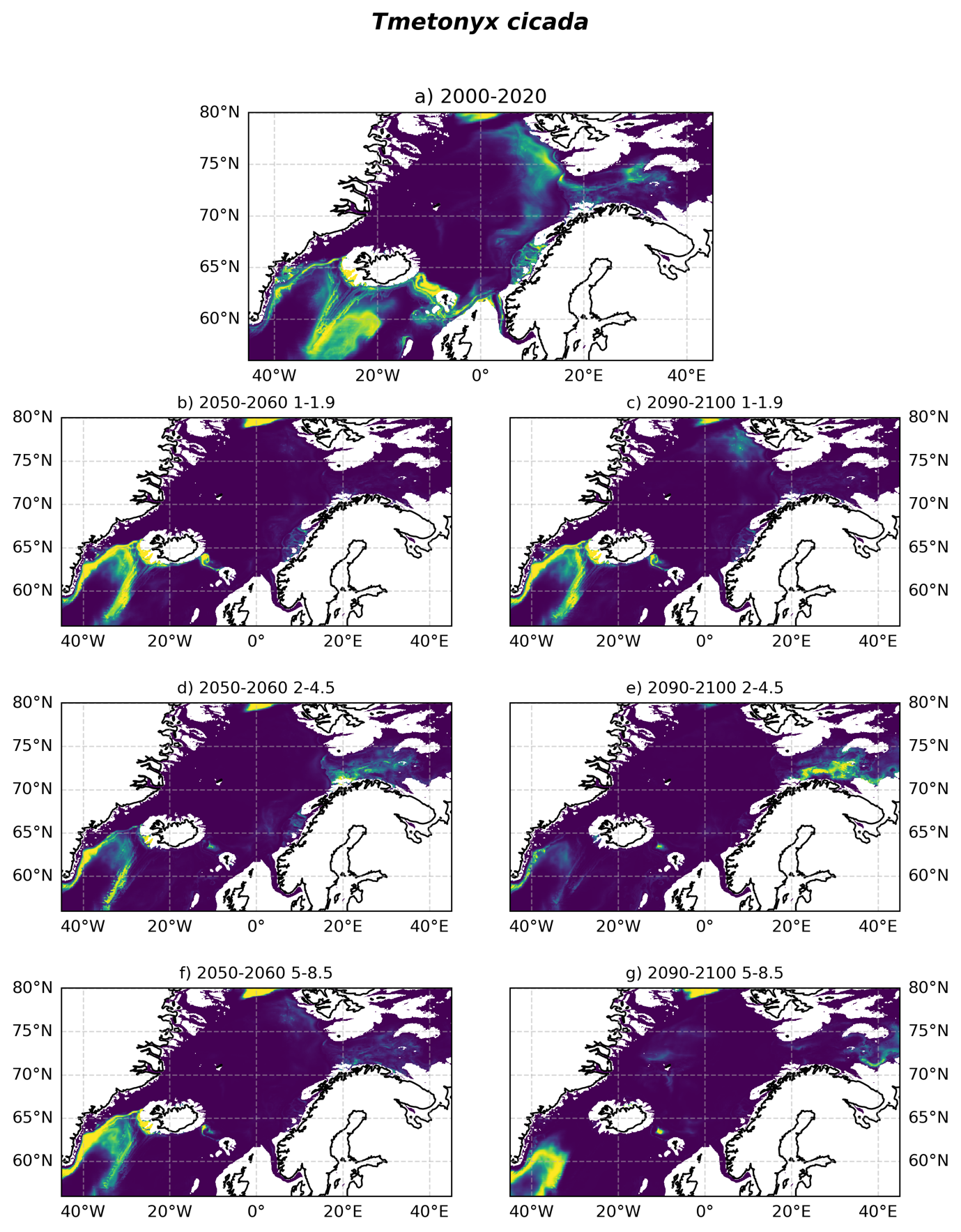


Figure S13: Maps show the habitat suitability of *Tmetonyx cicada* for a) present day, b) 2050–2060 and 1–1.9 SSP scenario, c) 2090–2100 and 1–1.9 SSP scenario, d) 2050–2060 and 2–4.5 SSP scenario, e) 2090–2100 and 2–4.5 SSP scenario, f) 2050–2060 and 5–8.5 SSP scenario, and g) 2090–2100 and 5–8.5 SSP. Purple indicating unsuitable habitat, yellow indicating highly suitable habitat.


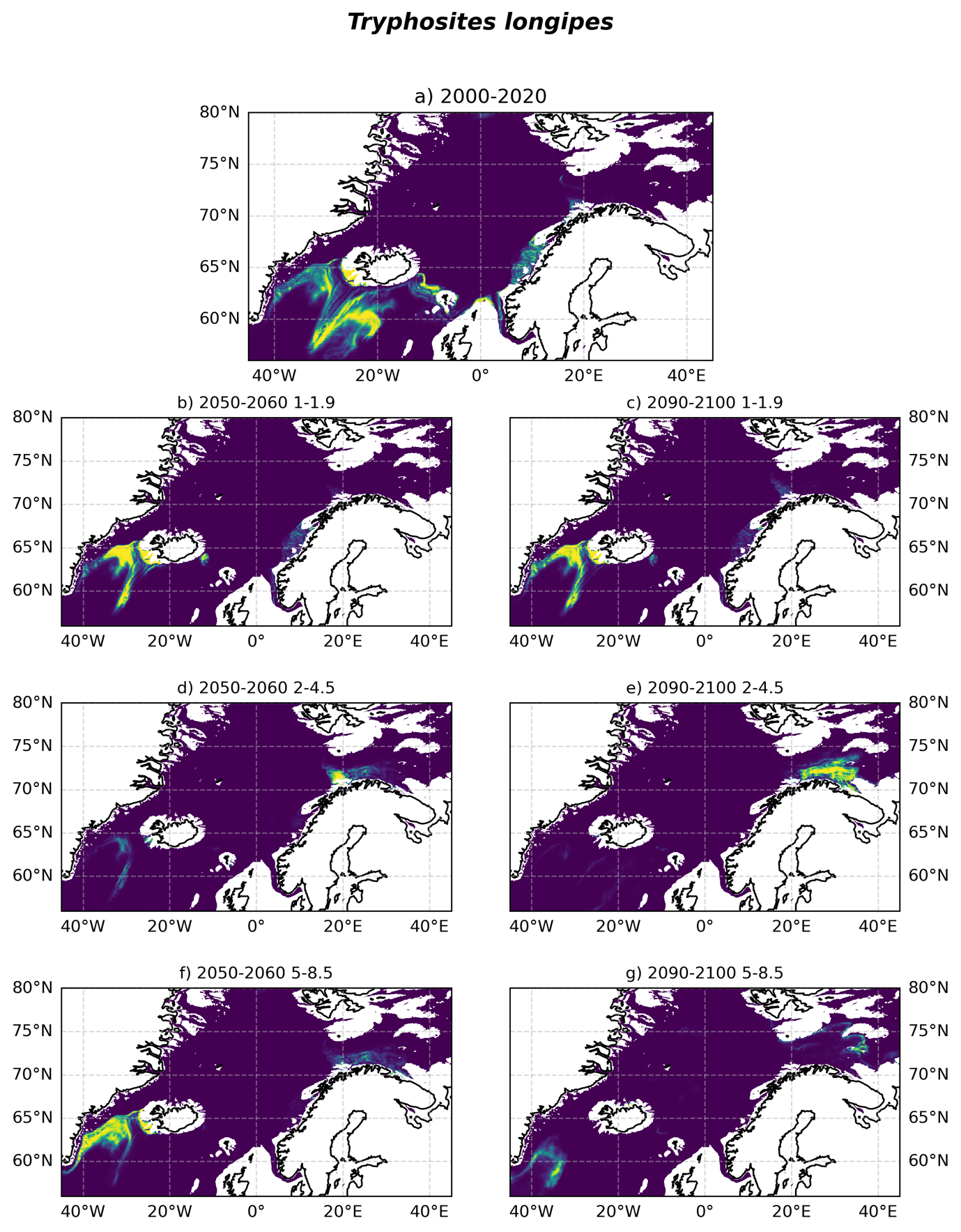


Figure S14: Maps show the habitat suitability of *Tryphosites longipes* for a) present day, b) 2050–2060 and 1–1.9 SSP scenario, c) 2090–2100 and 1–1.9 SSP scenario, d) 2050–2060 and 2–4.5 SSP scenario, e) 2090–2100 and 2–4.5 SSP scenario, f) 2050–2060 and 5–8.5 SSP scenario, and g) 2090–2100 and 5–8.5 SSP. Purple indicating unsuitable habitat, yellow indicating highly suitable habitat.


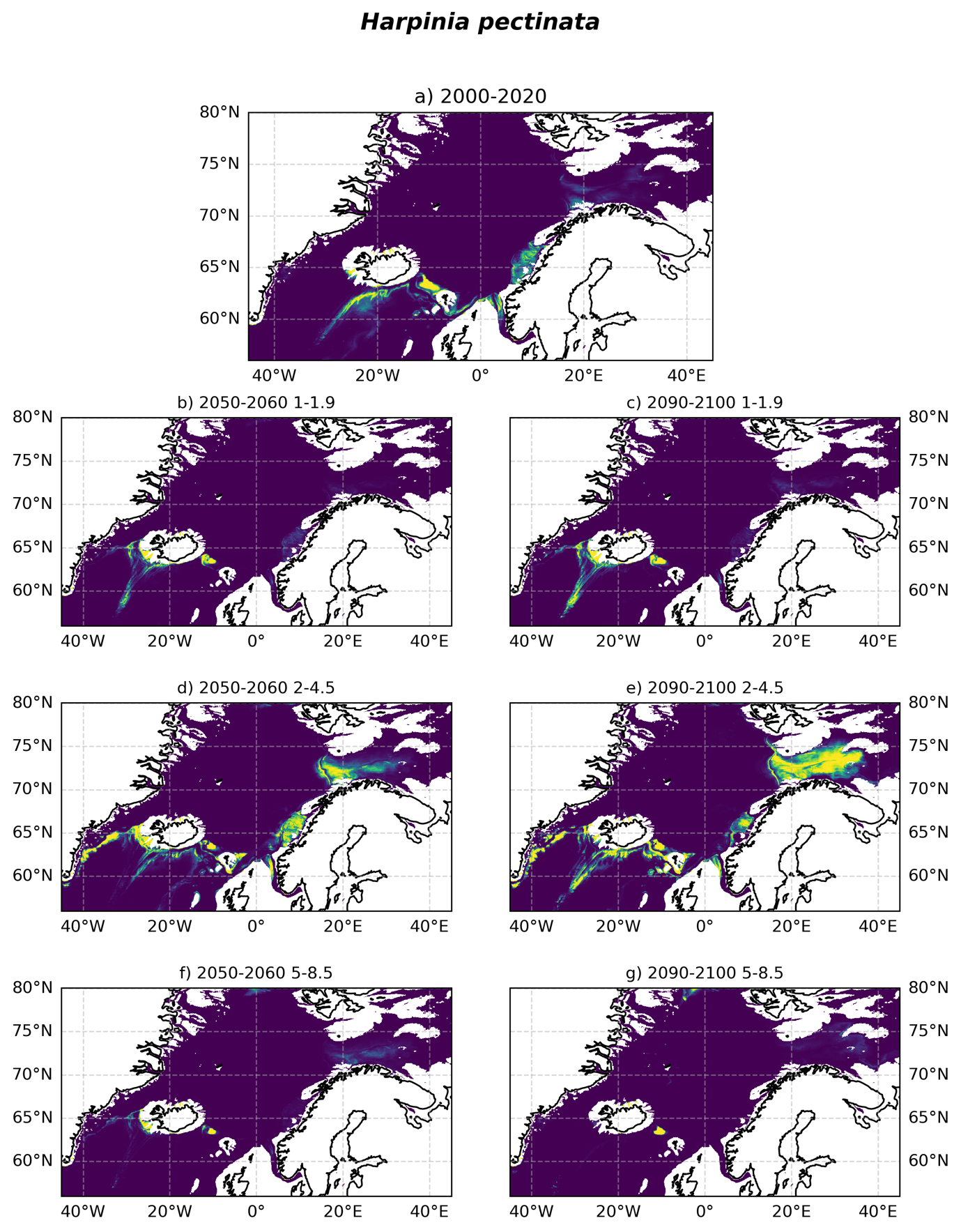


Figure S15: Maps show the habitat suitability of *Harpinia pectinata* for a) present day, b) 2050–2060 and 1–1.9 SSP scenario, c) 2090–2100 and 1–1.9 SSP scenario, d) 2050–2060 and 2–4.5 SSP scenario, e) 2090–2100 and 2–4.5 SSP scenario, f) 2050–2060 and 5–8.5 SSP scenario, and g) 2090–2100 and 5–8.5 SSP. Purple indicating unsuitable habitat, yellow indicating highly suitable habitat.


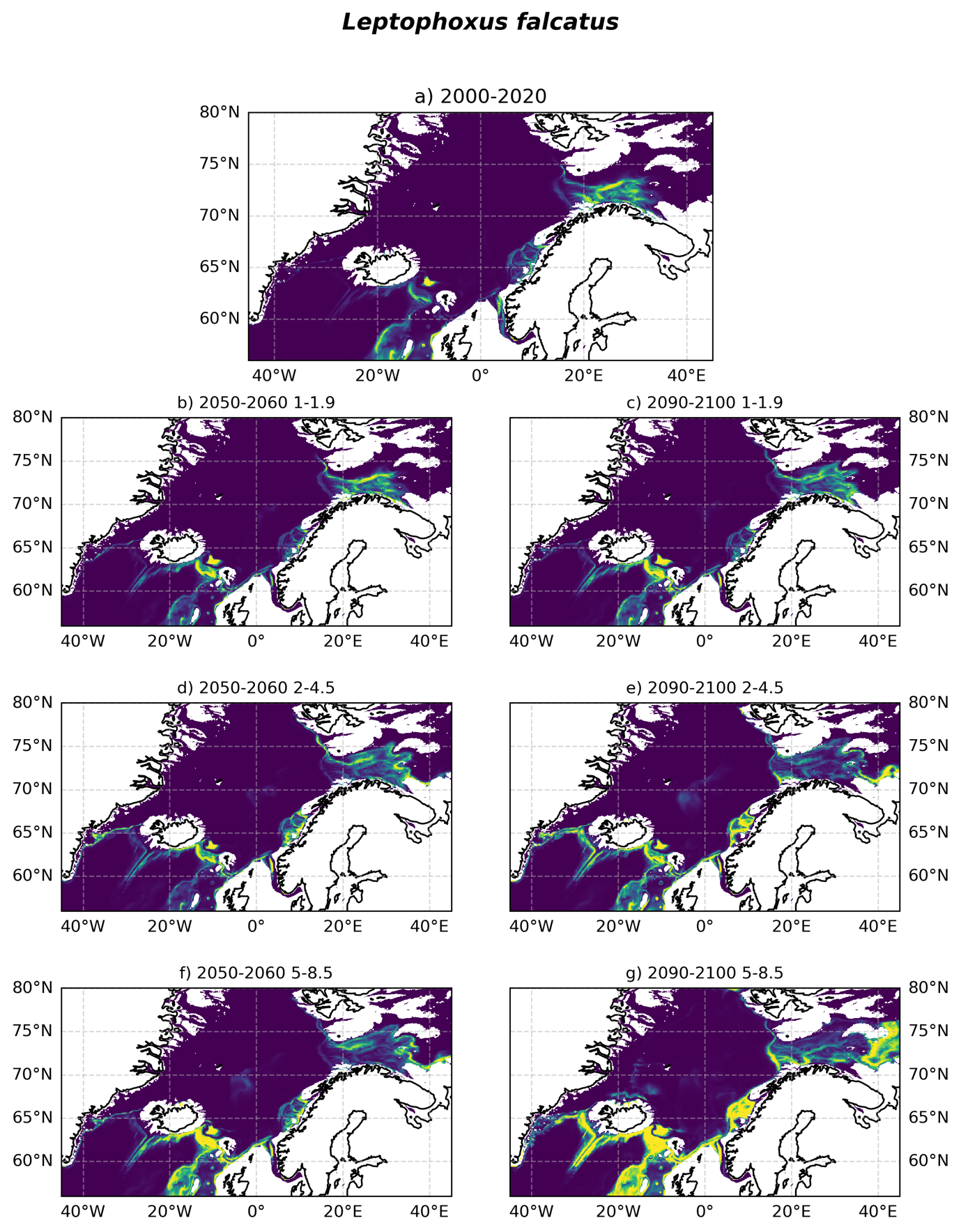


Figure S16: Maps show the habitat suitability of *Leptophoxus falcatus*

for a) present day, b) 2050–2060 and 1–1.9 SSP scenario, c) 2090–2100 and 1–1.9 SSP scenario, d) 2050–2060 and 2–4.5 SSP scenario, e) 2090–2100 and 2–4.5 SSP scenario, f) 2050–2060 and 5–8.5 SSP scenario, and g) 2090–2100 and 5–8.5 SSP. Purple indicating unsuitable habitat, yellow indicating highly suitable habitat.


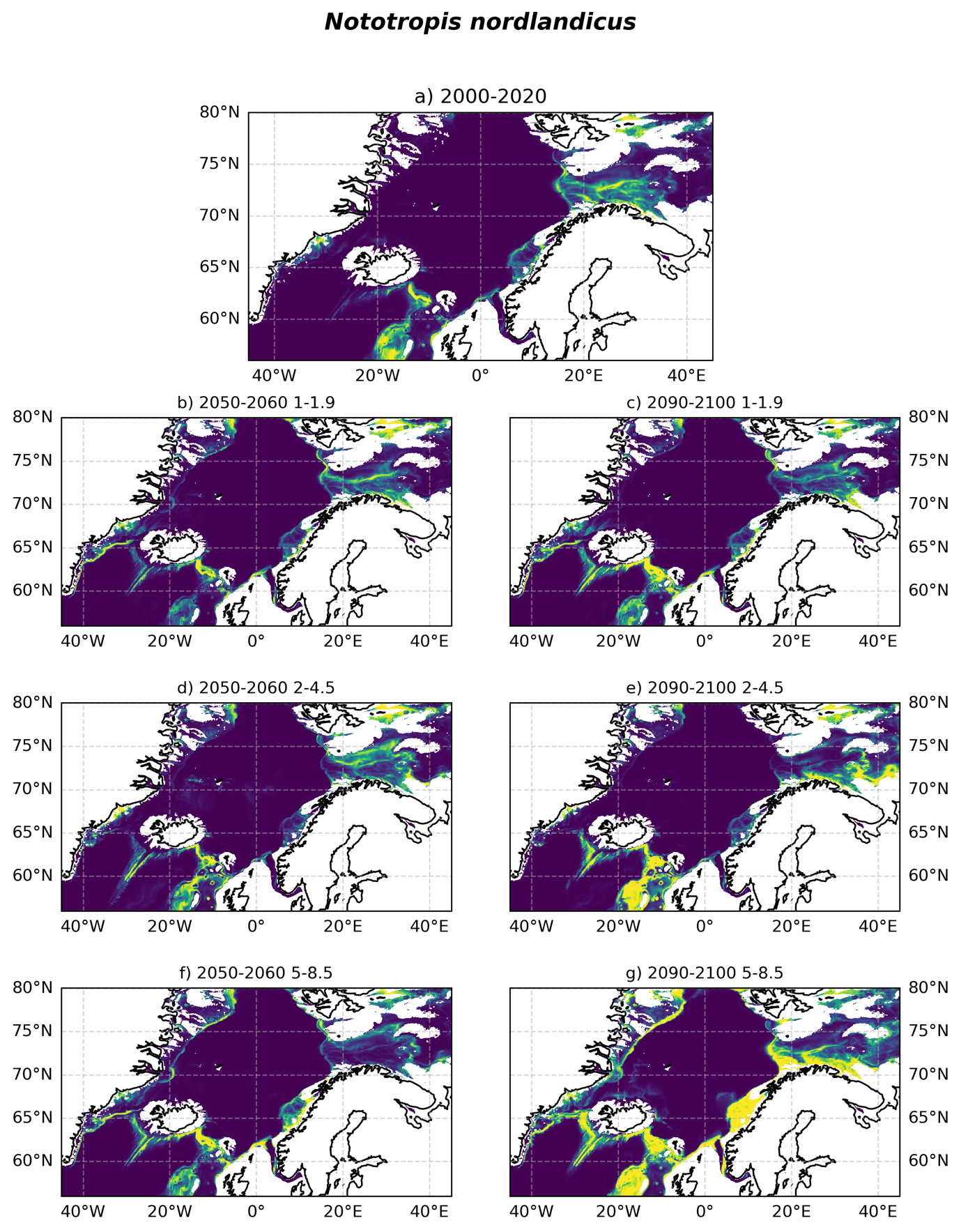


Figure S17: Maps show the habitat suitability of *Nototropis nordlandicus* for a) present day, b) 2050–2060 and 1–1.9 SSP scenario, c) 2090–2100 and 1–1.9 SSP scenario, d) 2050–2060 and 2–4.5 SSP scenario, e) 2090–2100 and 2–4.5 SSP scenario, f) 2050–2060 and 5–8.5 SSP scenario, and g) 2090–2100 and 5–8.5 SSP. Purple indicating unsuitable habitat, yellow indicating highly suitable habitat.


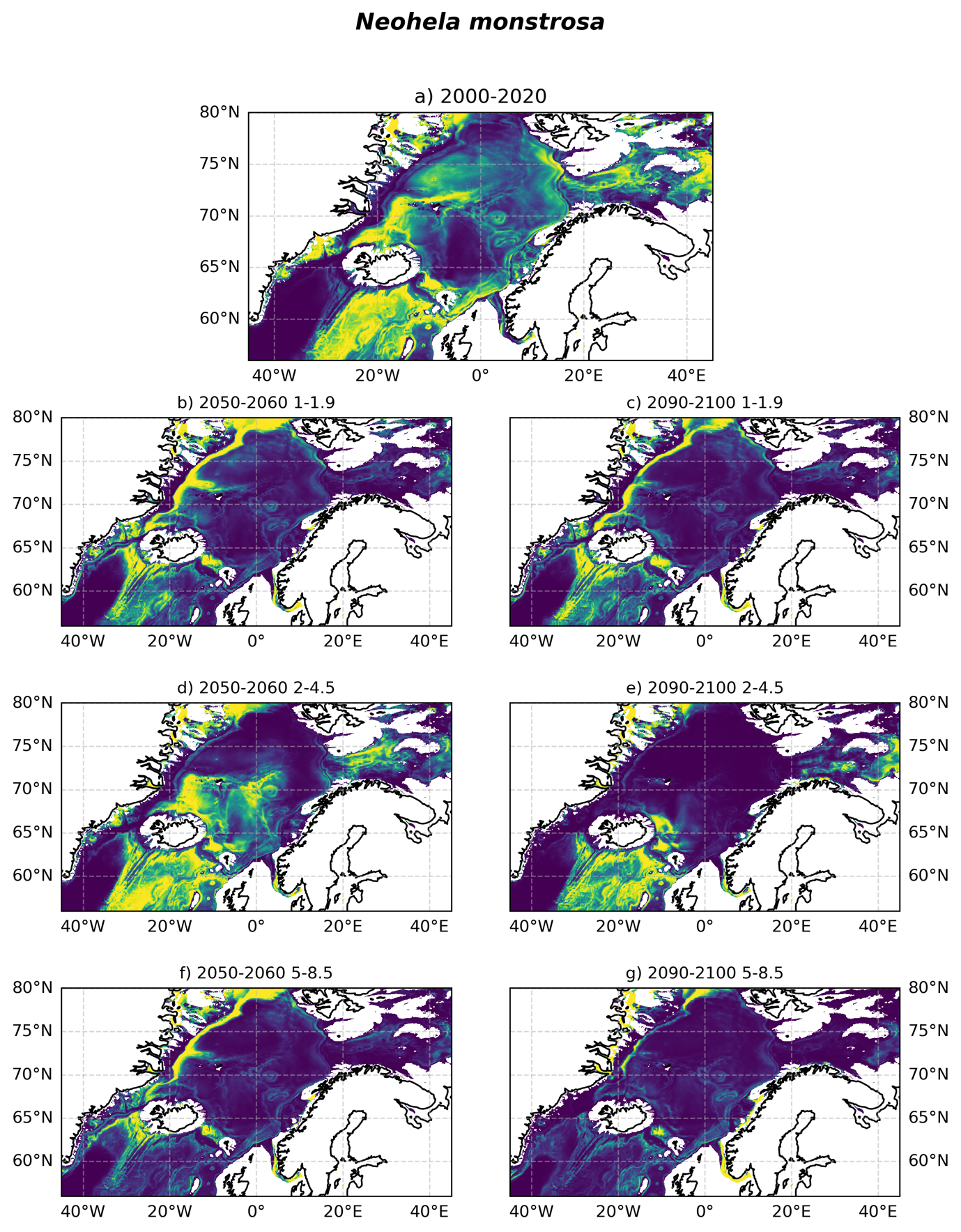


Figure S18: Maps show the habitat suitability of *Neohela monstrosa* for a) present day, b) 2050–2060 and 1–1.9 SSP scenario, c) 2090–2100 and 1–1.9 SSP scenario, d) 2050–2060 and 2–4.5 SSP scenario, e) 2090–2100 and 2–4.5 SSP scenario, f) 2050–2060 and 5–8.5 SSP scenario, and g) 2090–2100 and 5–8.5 SSP. Purple indicating unsuitable habitat, yellow indicating highly suitable habitat.


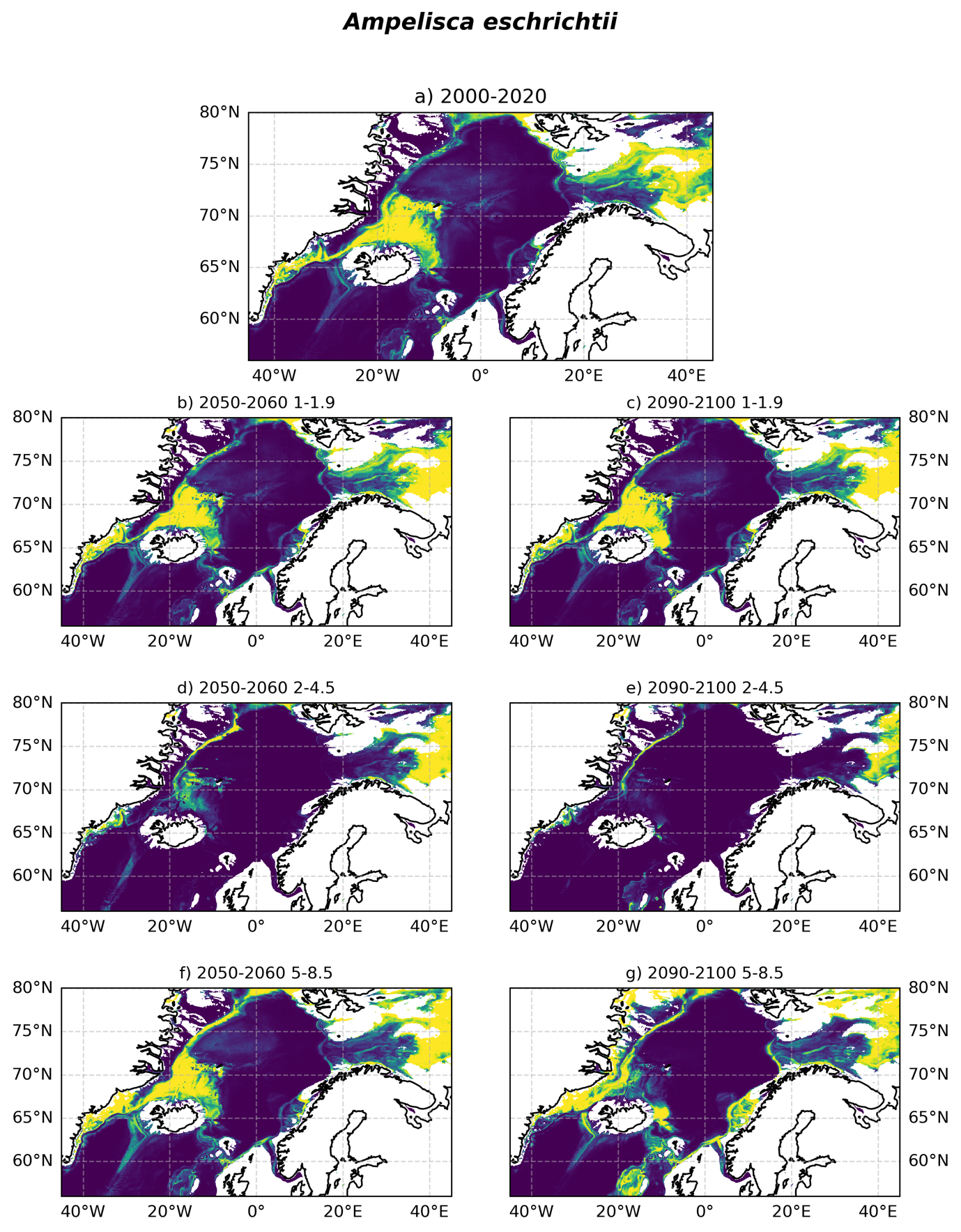


Figure S19: Maps show the habitat suitability of *Ampelisca eschrichtii* for a) present day, b) 2050–2060 and 1–1.9 SSP scenario, c) 2090–2100 and 1–1.9 SSP scenario, d) 2050–2060 and 2–4.5 SSP scenario, e) 2090–2100 and 2–4.5 SSP scenario, f) 2050–2060 and 5–8.5 SSP scenario, and g) 2090–2100 and 5–8.5 SSP. Purple indicating unsuitable habitat, yellow indicating highly suitable habitat.


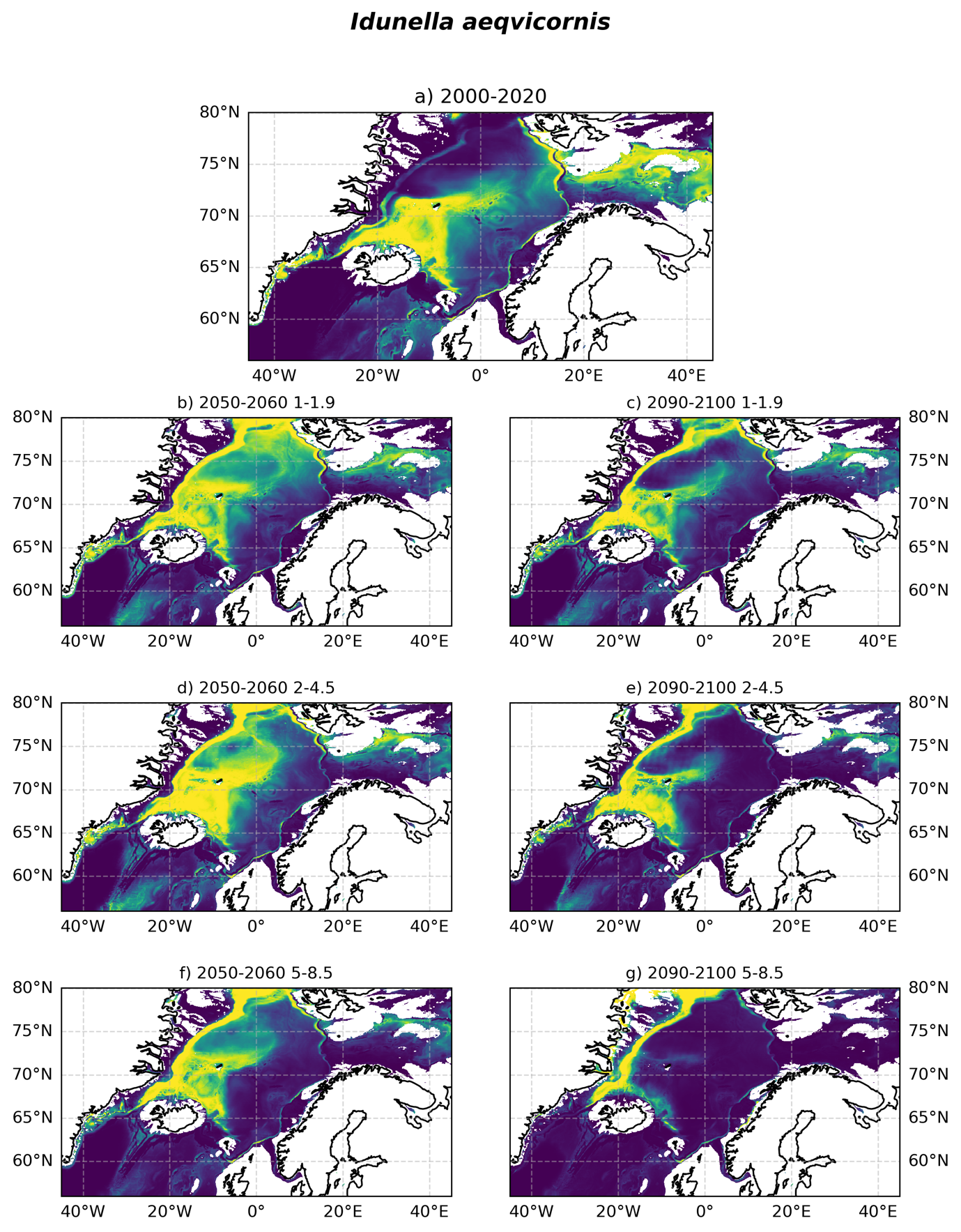


Figure S20: Maps show the habitat suitability of *Idunella aeqvicornis* for a) present day, b) 2050–2060 and 1–1.9 SSP scenario, c) 2090–2100 and 1–1.9 SSP scenario, d) 2050–2060 and 2–4.5 SSP scenario, e) 2090–2100 and 2–4.5 SSP scenario, f) 2050–2060 and 5–8.5 SSP scenario, and g) 2090–2100 and 5–8.5 SSP. Purple indicating unsuitable habitat, yellow indicating highly suitable habitat.


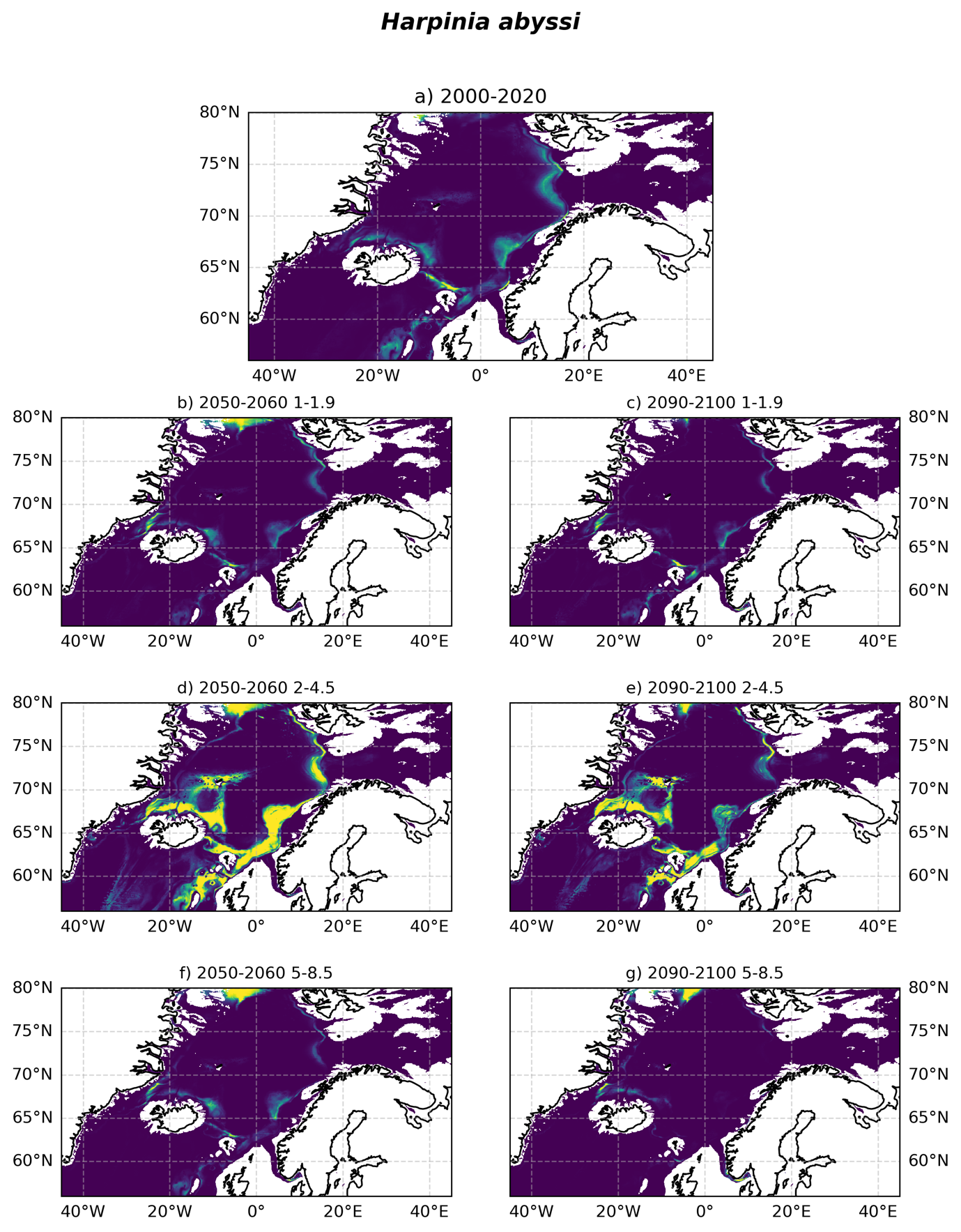


Figure S21: Maps show the habitat suitability of *Harpinia abyssi* for a) present day, b) 2050–2060 and 1–1.9 SSP scenario, c) 2090–2100 and 1–1.9 SSP scenario, d) 2050–2060 and 2–4.5 SSP scenario, e) 2090–2100 and 2–4.5 SSP scenario, f) 2050–2060 and 5–8.5 SSP scenario, and g) 2090–2100 and 5–8.5 SSP. Purple indicating unsuitable habitat, yellow indicating highly suitable habitat.


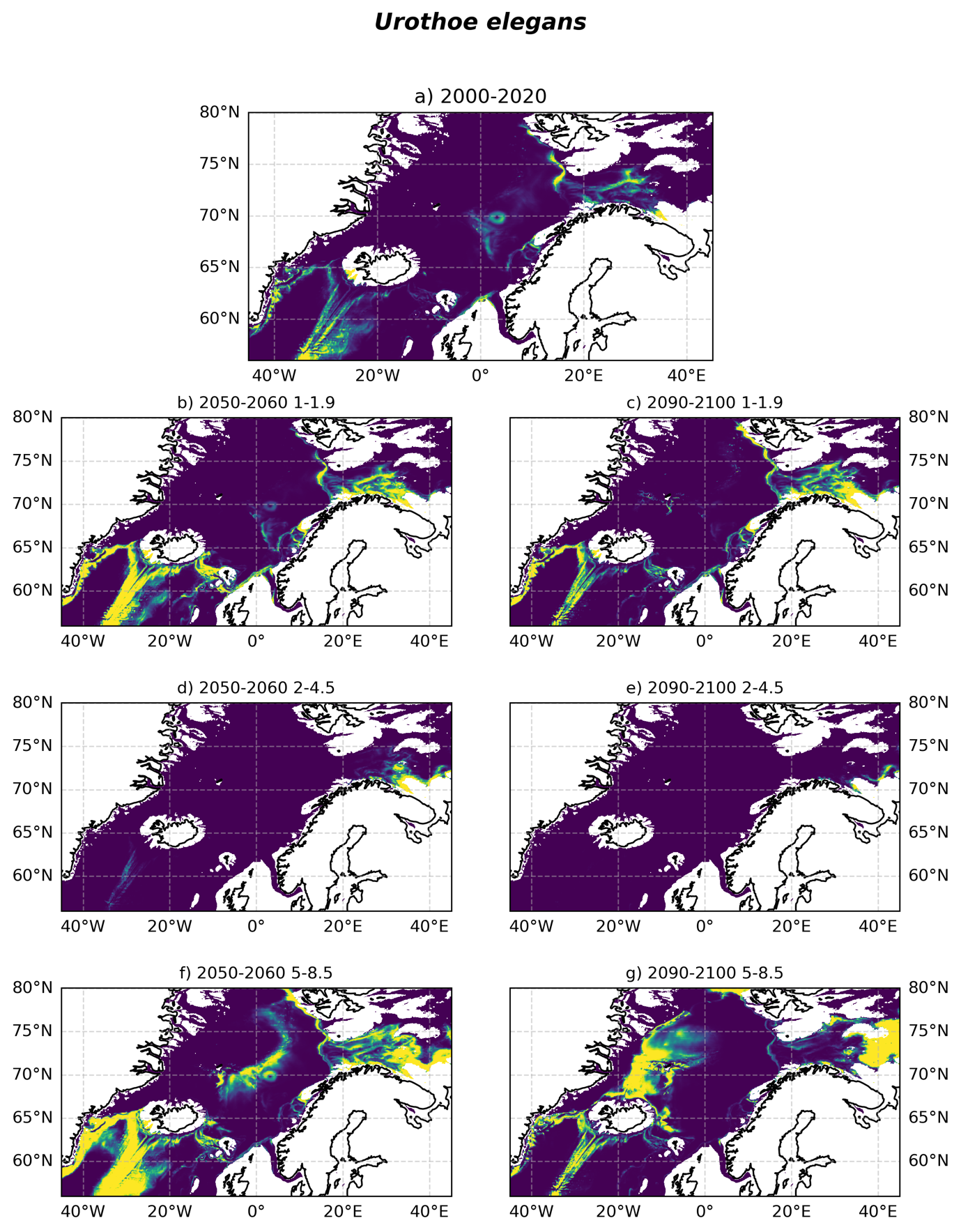


Figure S22: Maps show the habitat suitability of *Urothoe elegans* for a) present day, b) 2050–2060 and 1–1.9 SSP scenario, c) 2090–2100 and 1–1.9 SSP scenario, d) 2050–2060 and 2–4.5 SSP scenario, e) 2090–2100 and 2–4.5 SSP scenario, f) 2050–2060 and 5–8.5 SSP scenario, and g) 2090–2100 and 5–8.5 SSP. Purple indicating unsuitable habitat, yellow indicating highly suitable habitat.


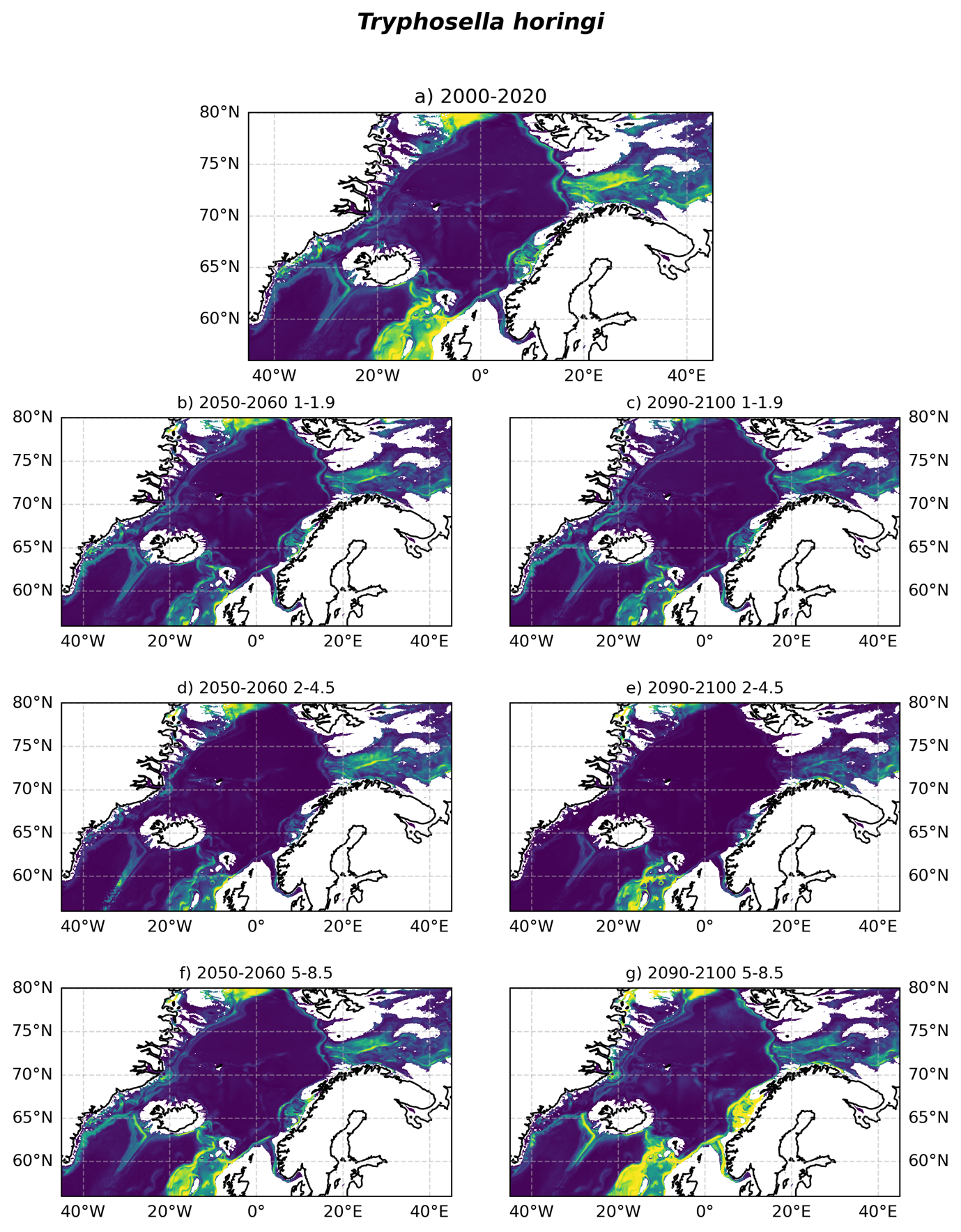


Figure S23: Maps show the habitat suitability of *Tryphosella horingi* for a) present day, b) 2050–2060 and 1–1.9 SSP scenario, c) 2090–2100 and 1–1.9 SSP scenario, d) 2050–2060 and 2–4.5 SSP scenario, e) 2090–2100 and 2–4.5 SSP scenario, f) 2050–2060 and 5–8.5 SSP scenario, and g) 2090–2100 and 5–8.5 SSP. Purple indicating unsuitable habitat, yellow indicating highly suitable habitat.


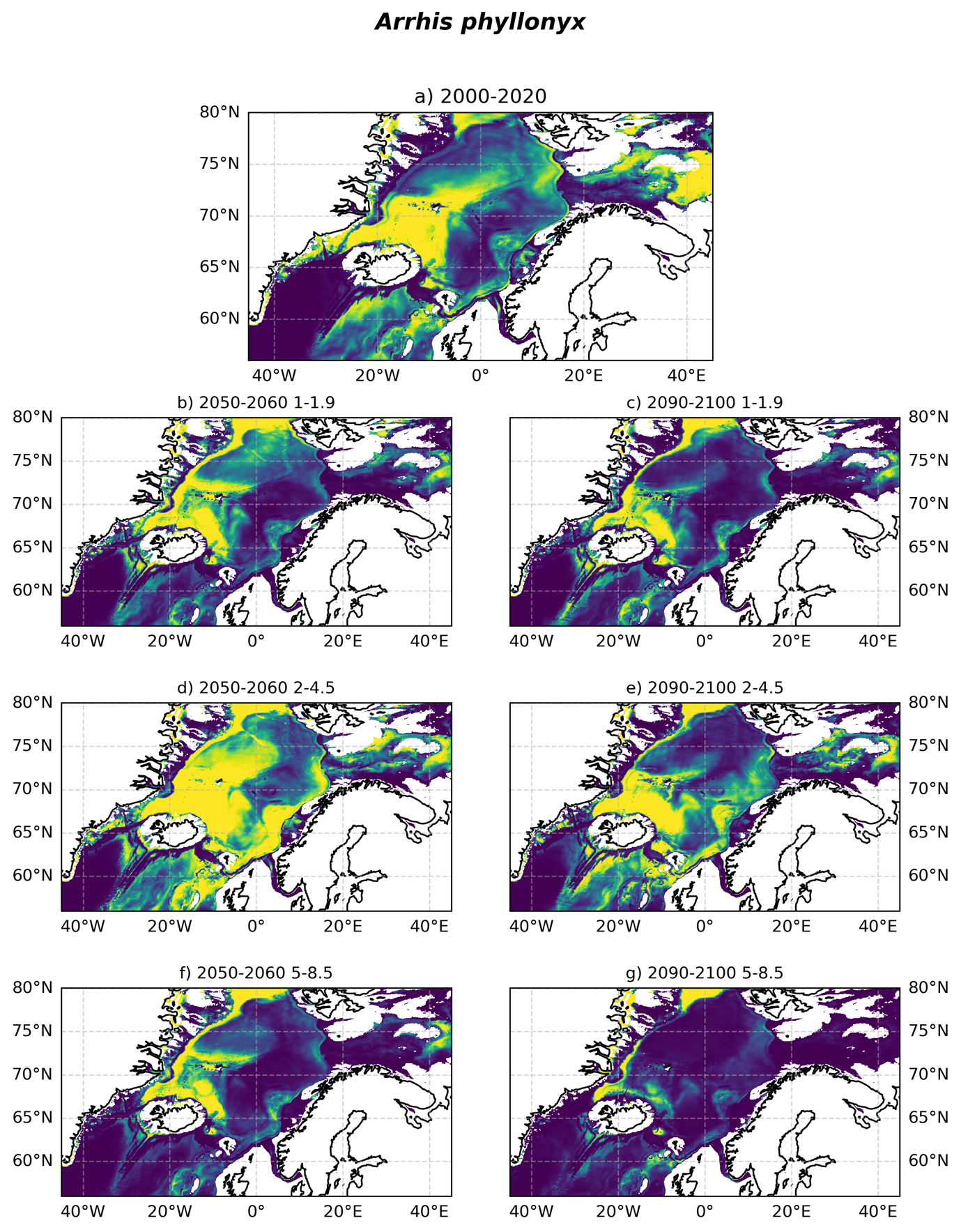


Figure S24: Maps show the habitat suitability of *Arrhis phyllonyx* for a) present day, b) 2050–2060 and 1–1.9 SSP scenario, c) 2090–2100 and 1–1.9 SSP scenario, d) 2050–2060 and 2–4.5 SSP scenario, e) 2090–2100 and 2–4.5 SSP scenario, f) 2050–2060 and 5–8.5 SSP scenario, and g) 2090–2100 and 5–8.5 SSP. Purple indicating unsuitable habitat, yellow indicating highly suitable habitat.


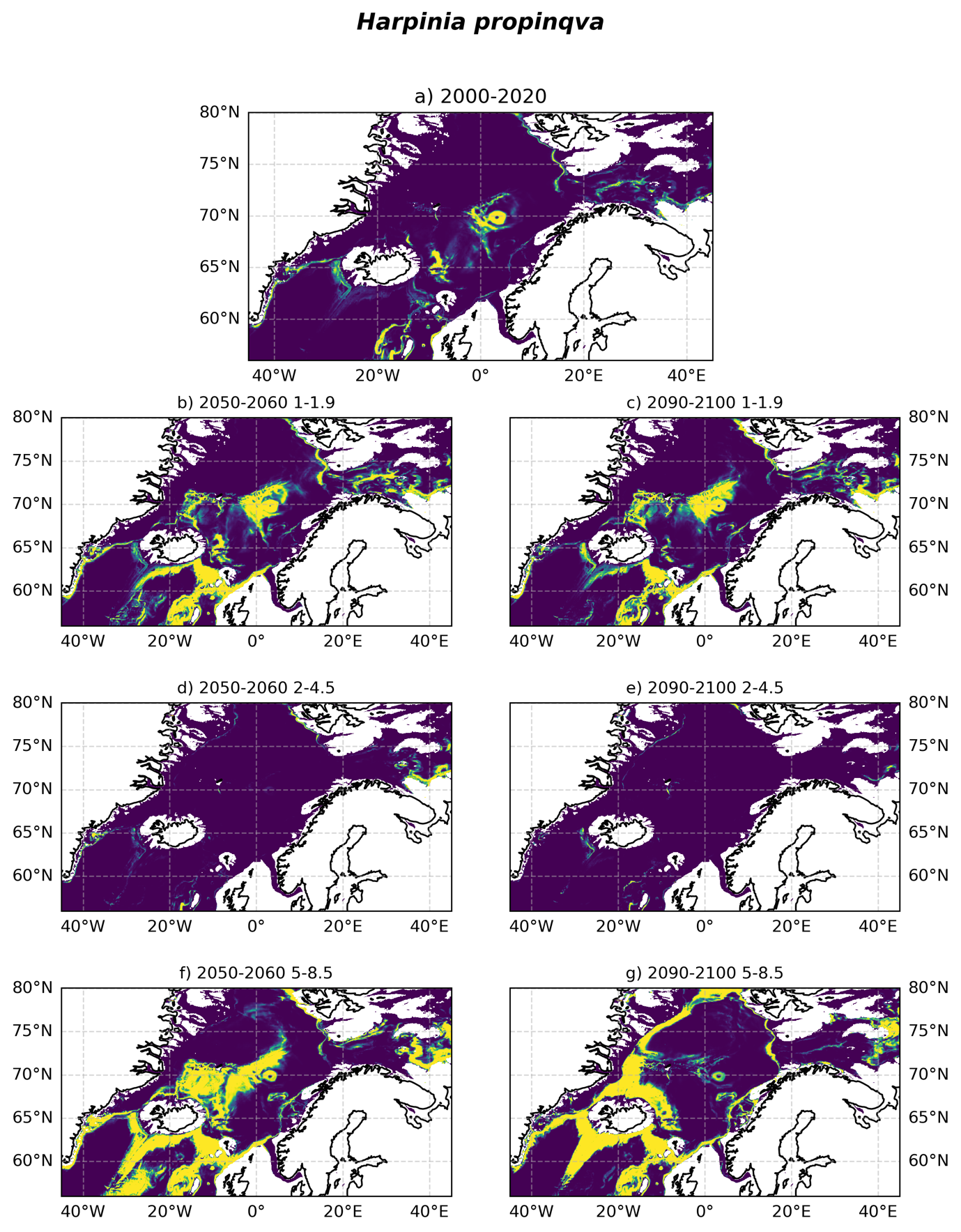


Figure S25: Maps show the habitat suitability of *Harpinia propinqva* for a) present day, b) 2050–2060 and 1–1.9 SSP scenario, c) 2090–2100 and 1–1.9 SSP scenario, d) 2050–2060 and 2–4.5 SSP scenario, e) 2090–2100 and 2–4.5 SSP scenario, f) 2050–2060 and 5–8.5 SSP scenario, and g) 2090–2100 and 5–8.5 SSP. Purple indicating unsuitable habitat, yellow indicating highly suitable habitat.


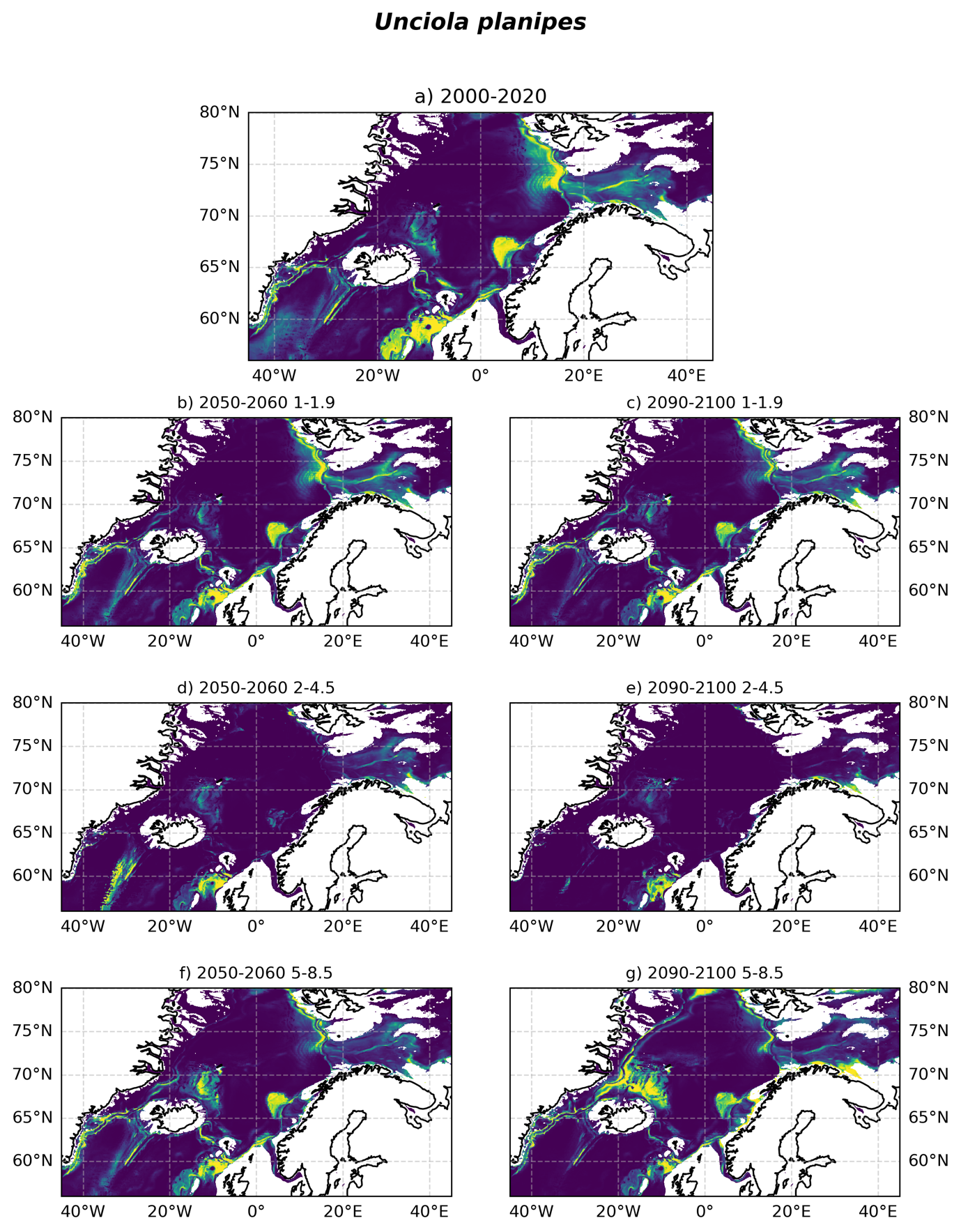


Figure S26: Maps show the habitat suitability of *Unciola planipes* for a) present day, b) 2050–2060 and 1–1.9 SSP scenario, c) 2090–2100 and 1–1.9 SSP scenario, d) 2050–2060 and 2–4.5 SSP scenario, e) 2090–2100 and 2–4.5 SSP scenario, f) 2050–2060 and 5–8.5 SSP scenario, and g) 2090–2100 and 5–8.5 SSP. Purple indicating unsuitable habitat, yellow indicating highly suitable habitat.


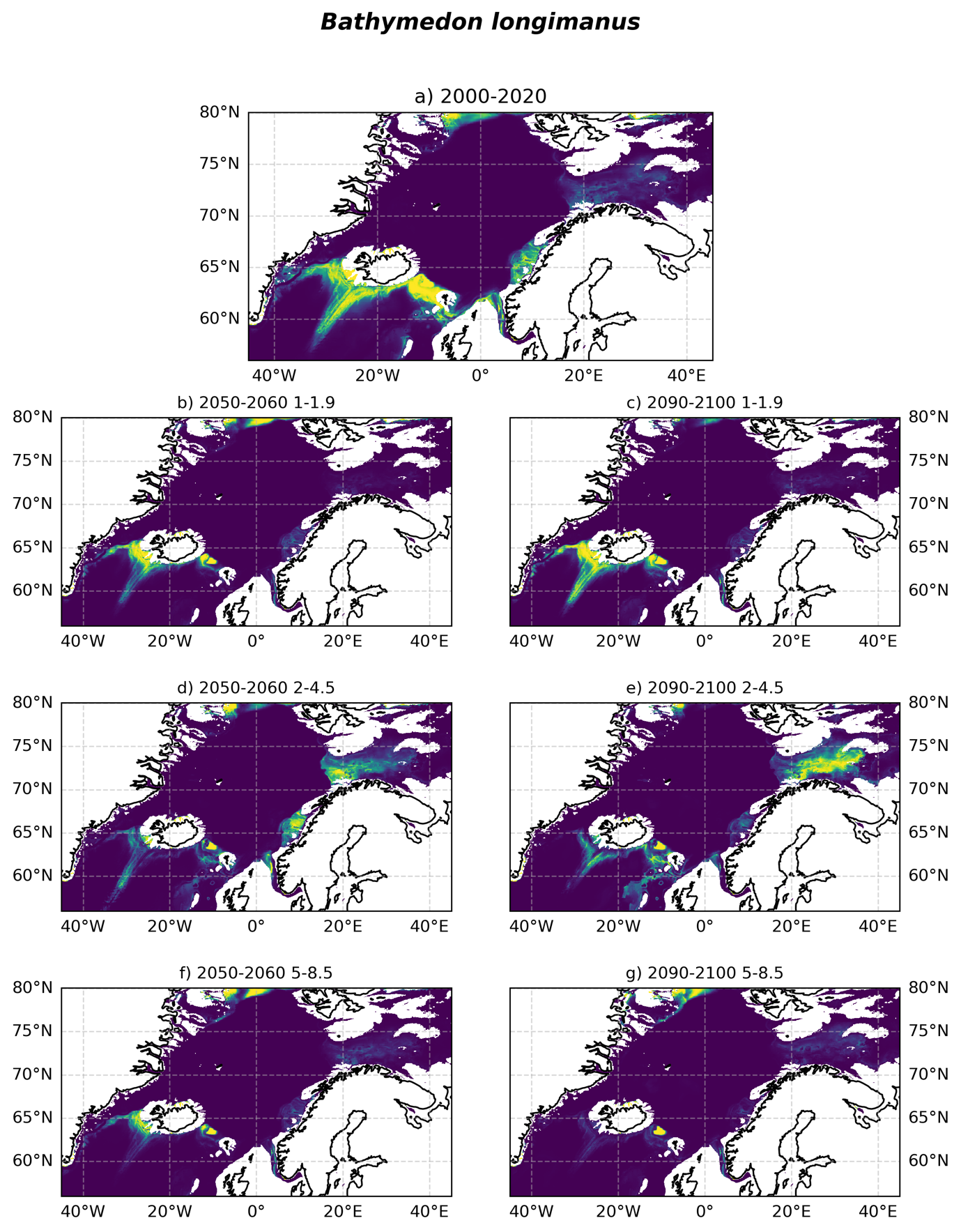


Figure S27: Maps show the habitat suitability of *Bathymedon longimanus* for a) present day, b) 2050–2060 and 1–1.9 SSP scenario, c) 2090–2100 and 1–1.9 SSP scenario, d) 2050–2060 and 2–4.5 SSP scenario, e) 2090–2100 and 2–4.5 SSP scenario, f) 2050–2060 and 5–8.5 SSP scenario, and g) 2090–2100 and 5–8.5 SSP. Purple indicating unsuitable habitat, yellow indicating highly suitable habitat.


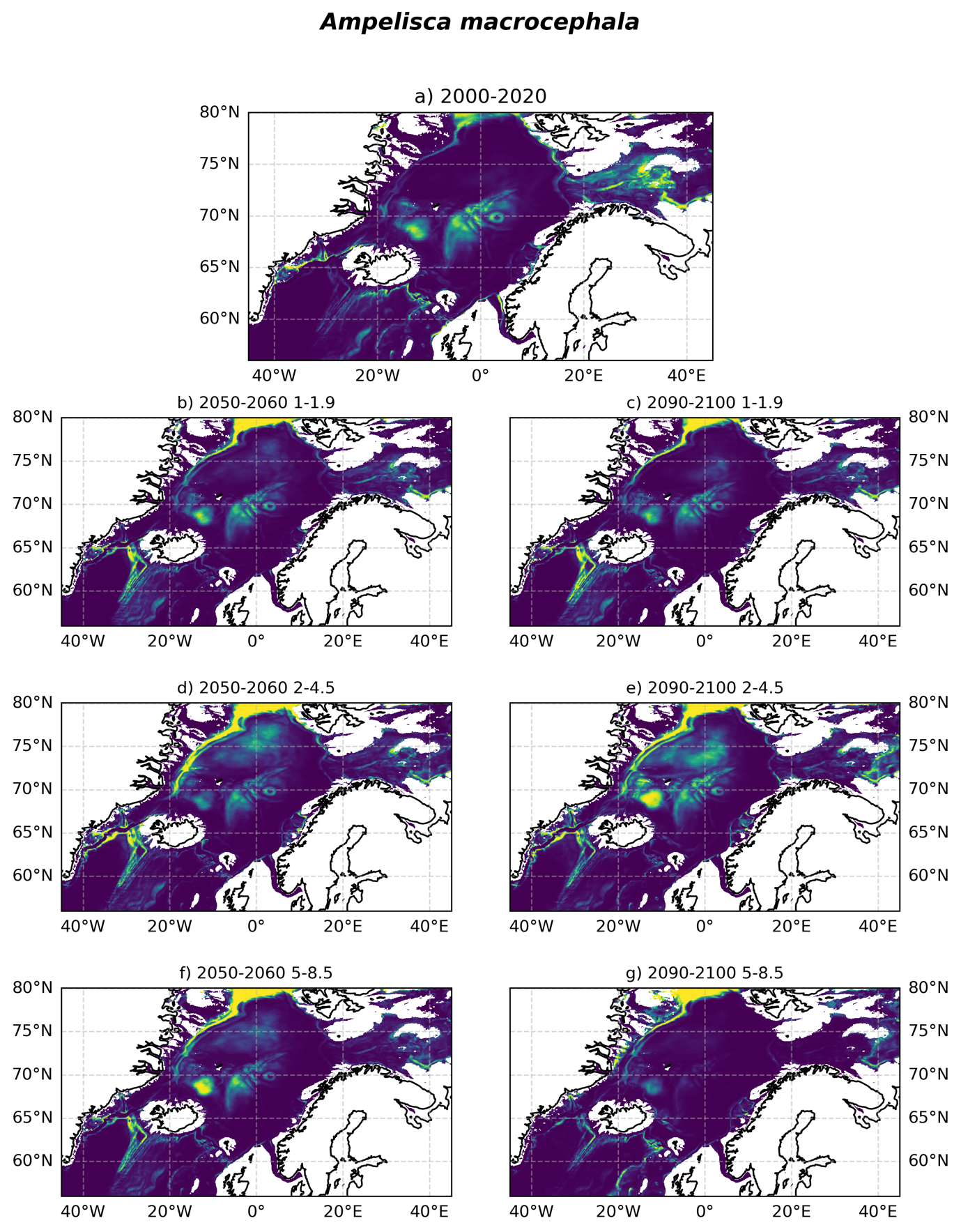


Figure S28: Maps show the habitat suitability of *Ampelisca macrocephala* for a) present day, b) 2050–2060 and 1–1.9 SSP scenario, c) 2090–2100 and 1–1.9 SSP scenario, d) 2050–2060 and 2–4.5 SSP scenario, e) 2090–2100 and 2–4.5 SSP scenario, f) 2050–2060 and 5–8.5 SSP scenario, and g) 2090–2100 and 5–8.5 SSP. Purple indicating unsuitable habitat, yellow indicating highly suitable habitat.


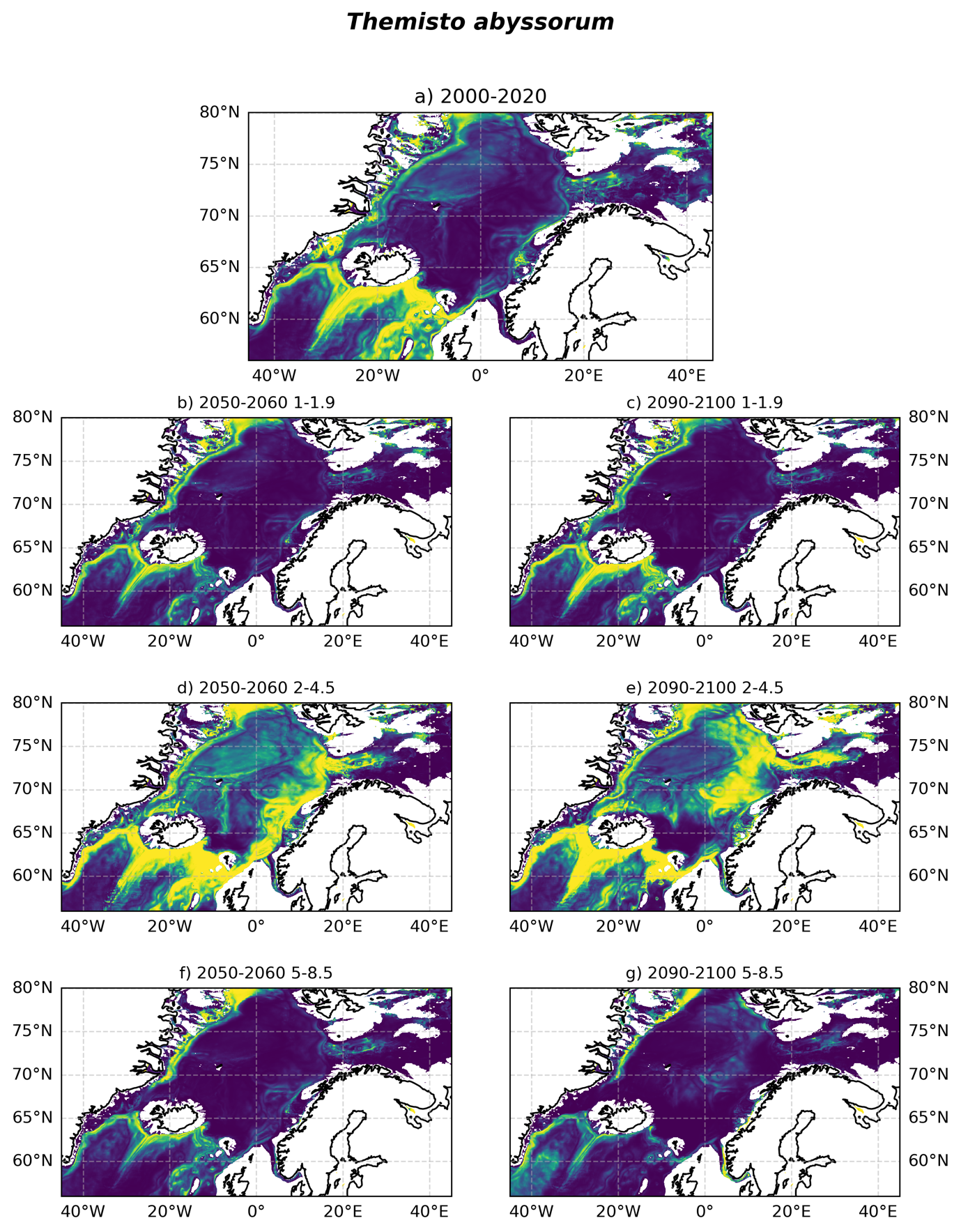


Figure S29: Maps show the habitat suitability of *Themisto abyssorum* for a) present day, b) 2050–2060 and 1–1.9 SSP scenario, c) 2090–2100 and 1–1.9 SSP scenario, d) 2050–2060 and 2–4.5 SSP scenario, e) 2090–2100 and 2–4.5 SSP scenario, f) 2050–2060 and 5–8.5 SSP scenario, and g) 2090–2100 and 5–8.5 SSP. Purple indicating unsuitable habitat, yellow indicating highly suitable habitat.


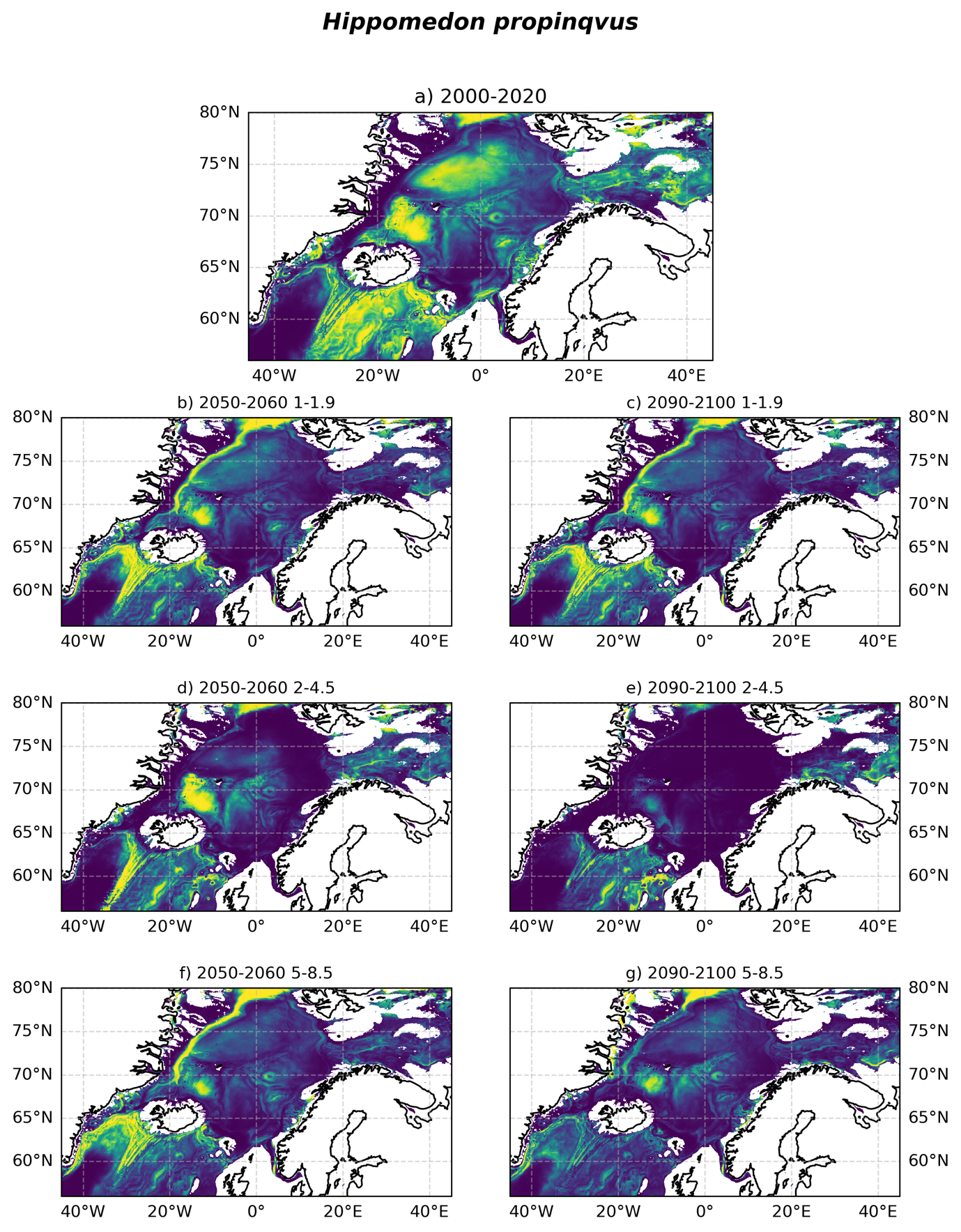


Figure S30: Maps show the habitat suitability of *Hippomedon propinqvus* for a) present day, b) 2050–2060 and 1–1.9 SSP scenario, c) 2090–2100 and 1–1.9 SSP scenario, d) 2050–2060 and 2–4.5 SSP scenario, e) 2090–2100 and 2–4.5 SSP scenario, f) 2050–2060 and 5–8.5 SSP scenario, and g) 2090–2100 and 5–8.5 SSP. Purple indicating unsuitable habitat, yellow indicating highly suitable habitat.


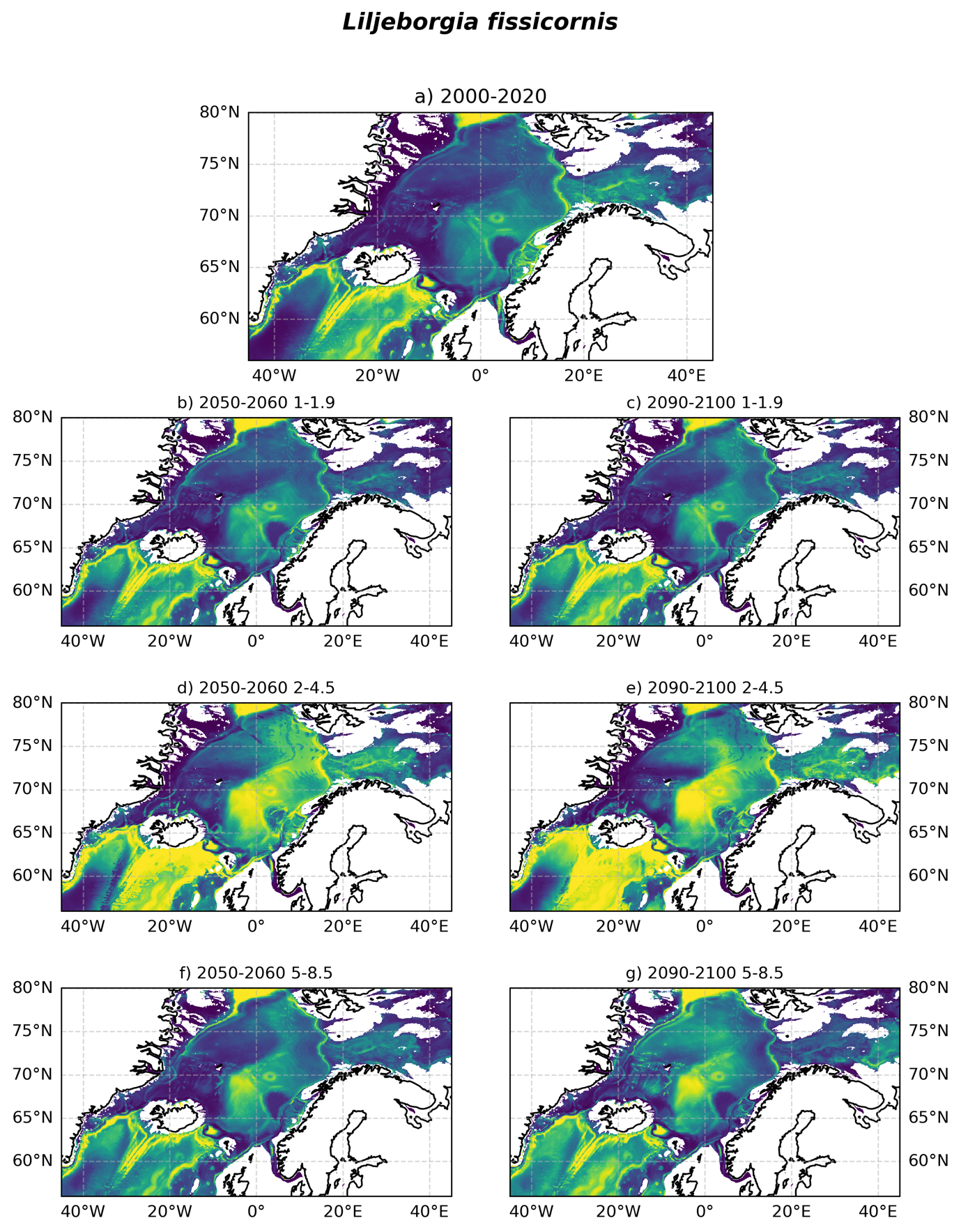


Figure S31: Maps show the habitat suitability of *Liljeborgia fissicornis* for a) present day, b) 2050–2060 and 1–1.9 SSP scenario, c) 2090–2100 and 1–1.9 SSP scenario, d) 2050–2060 and 2–4.5 SSP scenario, e) 2090–2100 and 2–4.5 SSP scenario, f) 2050–2060 and 5–8.5 SSP scenario, and g) 2090–2100 and 5–8.5 SSP. Purple indicating unsuitable habitat, yellow indicating highly suitable habitat.


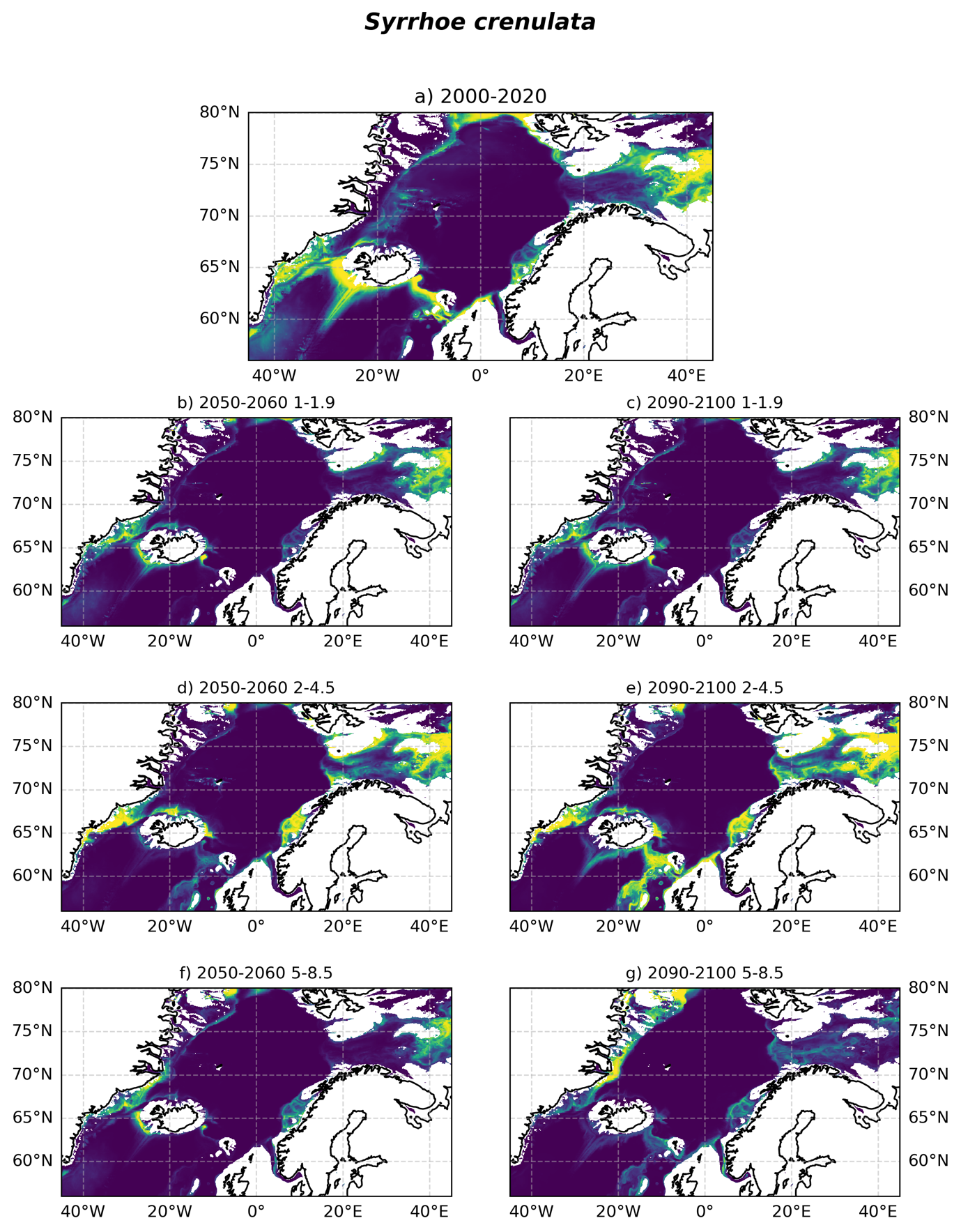


Figure S32: Maps show the habitat suitability of *Syrrhoe crenulata* for a) present day, b) 2050–2060 and 1–1.9 SSP scenario, c) 2090–2100 and 1–1.9 SSP scenario, d) 2050–2060 and 2–4.5 SSP scenario, e) 2090–2100 and 2–4.5 SSP scenario, f) 2050–2060 and 5–8.5 SSP scenario, and g) 2090–2100 and 5–8.5 SSP. Purple indicating unsuitable habitat, yellow indicating highly suitable habitat.


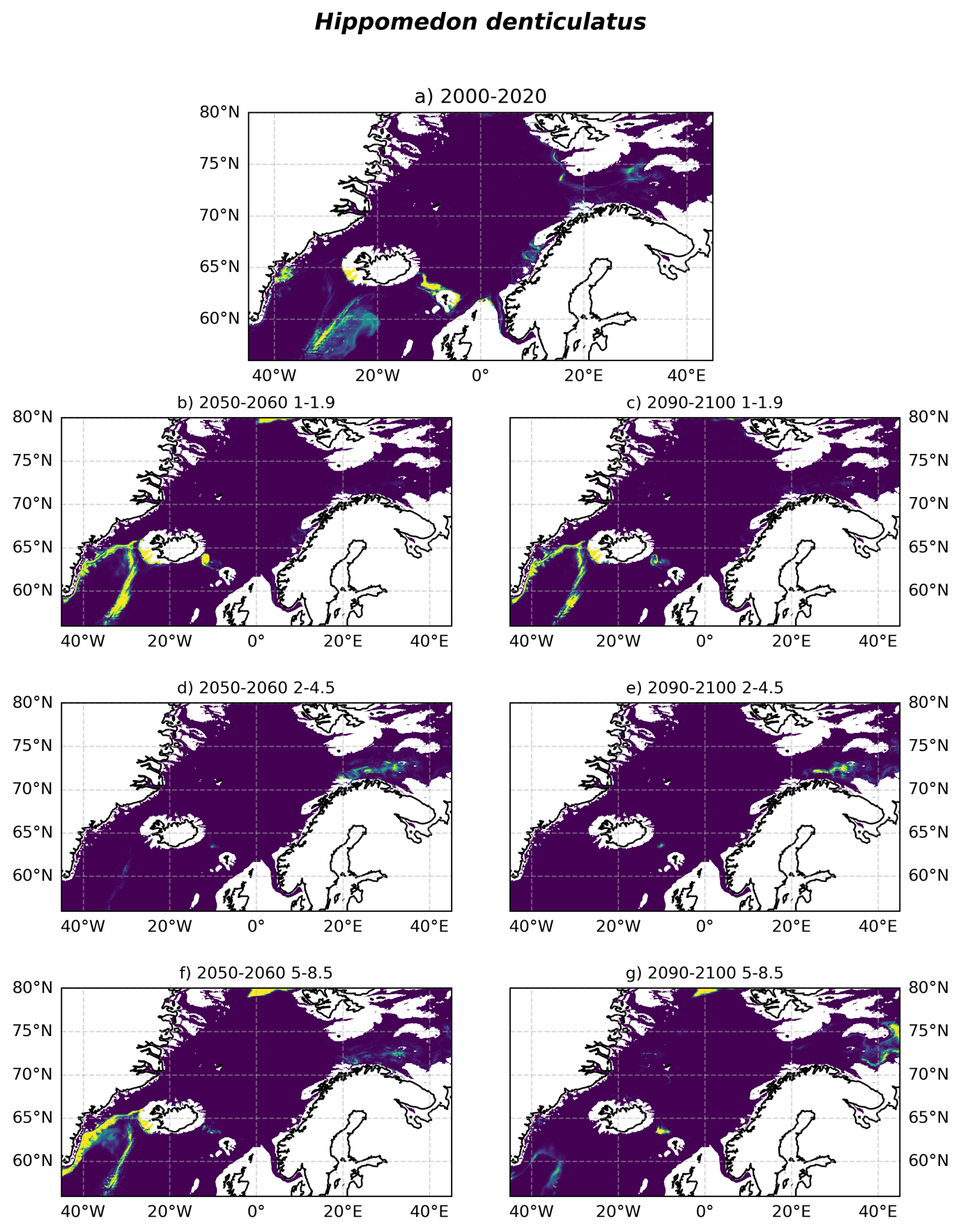


Figure S33: Maps show the habitat suitability of *Hippomedon denticulatus* for a) present day, b) 2050–2060 and 1–1.9 SSP scenario, c) 2090–2100 and 1–1.9 SSP scenario, d) 2050–2060 and 2–4.5 SSP scenario, e) 2090–2100 and 2–4.5 SSP scenario, f) 2050–2060 and 5–8.5 SSP scenario, and g) 2090–2100 and 5–8.5 SSP. Purple indicating unsuitable habitat, yellow indicating highly suitable habitat.


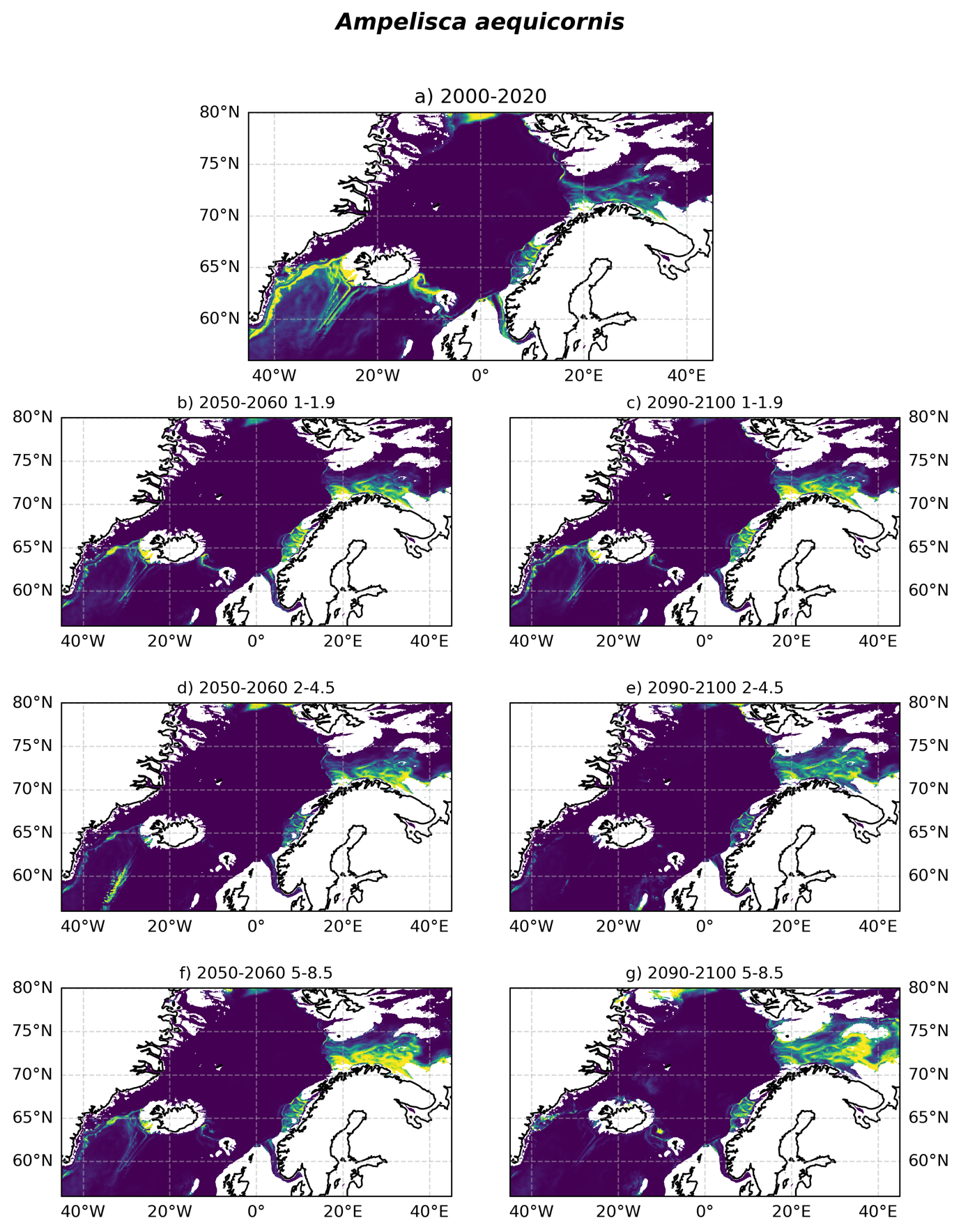


Figure S34: Maps show the habitat suitability of *Ampelisca aequicornis* for a) present day, b) 2050–2060 and 1–1.9 SSP scenario, c) 2090–2100 and 1–1.9 SSP scenario, d) 2050–2060 and 2–4.5 SSP scenario, e) 2090–2100 and 2–4.5 SSP scenario, f) 2050–2060 and 5–8.5 SSP scenario, and g) 2090–2100 and 5–8.5 SSP. Purple indicating unsuitable habitat, yellow indicating highly suitable habitat.


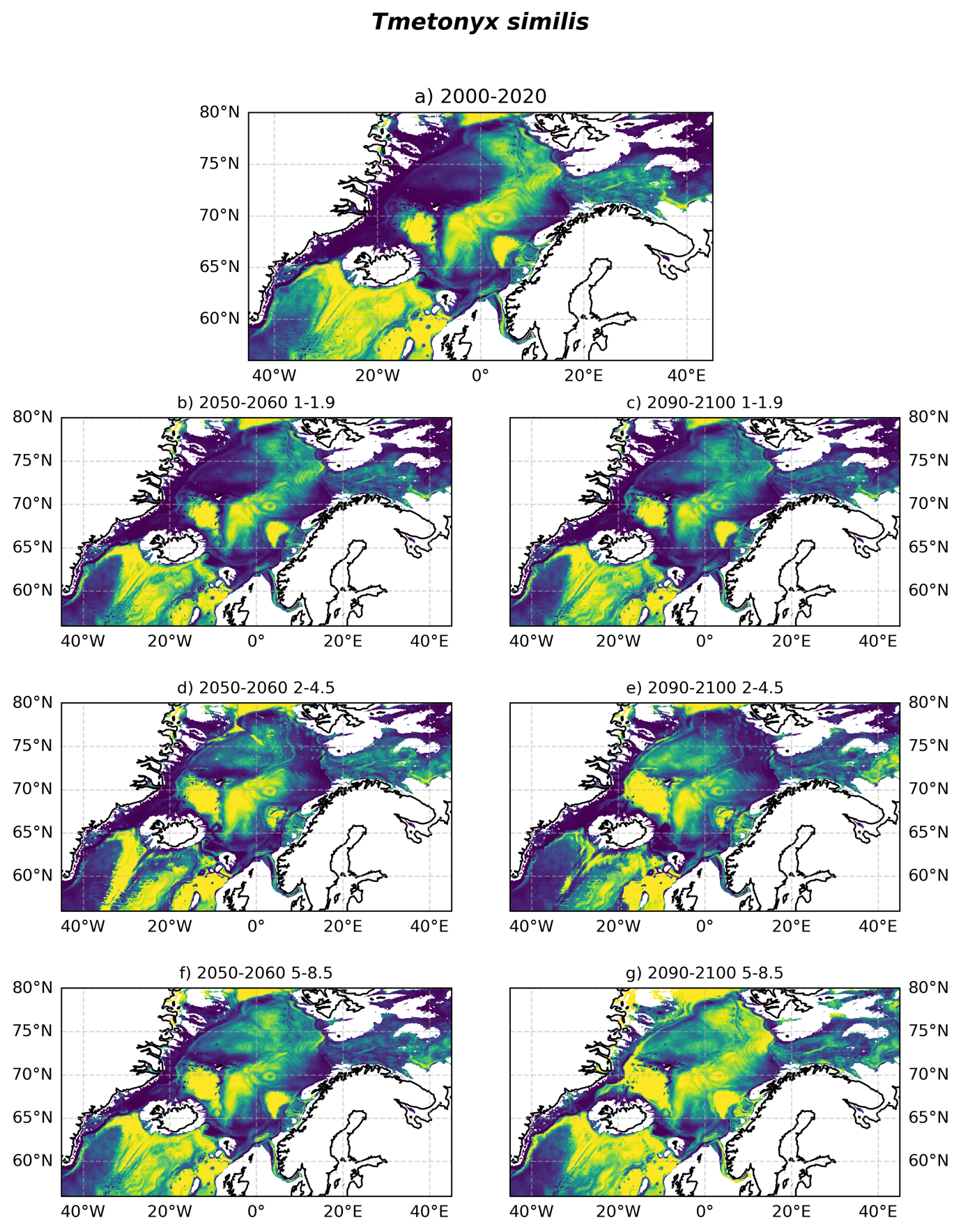


Figure S35: Maps show the habitat suitability of *Tmetonyx similis* for a) present day, b) 2050–2060 and 1–1.9 SSP scenario, c) 2090–2100 and 1–1.9 SSP scenario, d) 2050–2060 and 2–4.5 SSP scenario, e) 2090–2100 and 2–4.5 SSP scenario, f) 2050–2060 and 5–8.5 SSP scenario, and g) 2090–2100 and 5–8.5 SSP. Purple indicating unsuitable habitat, yellow indicating highly suitable habitat.


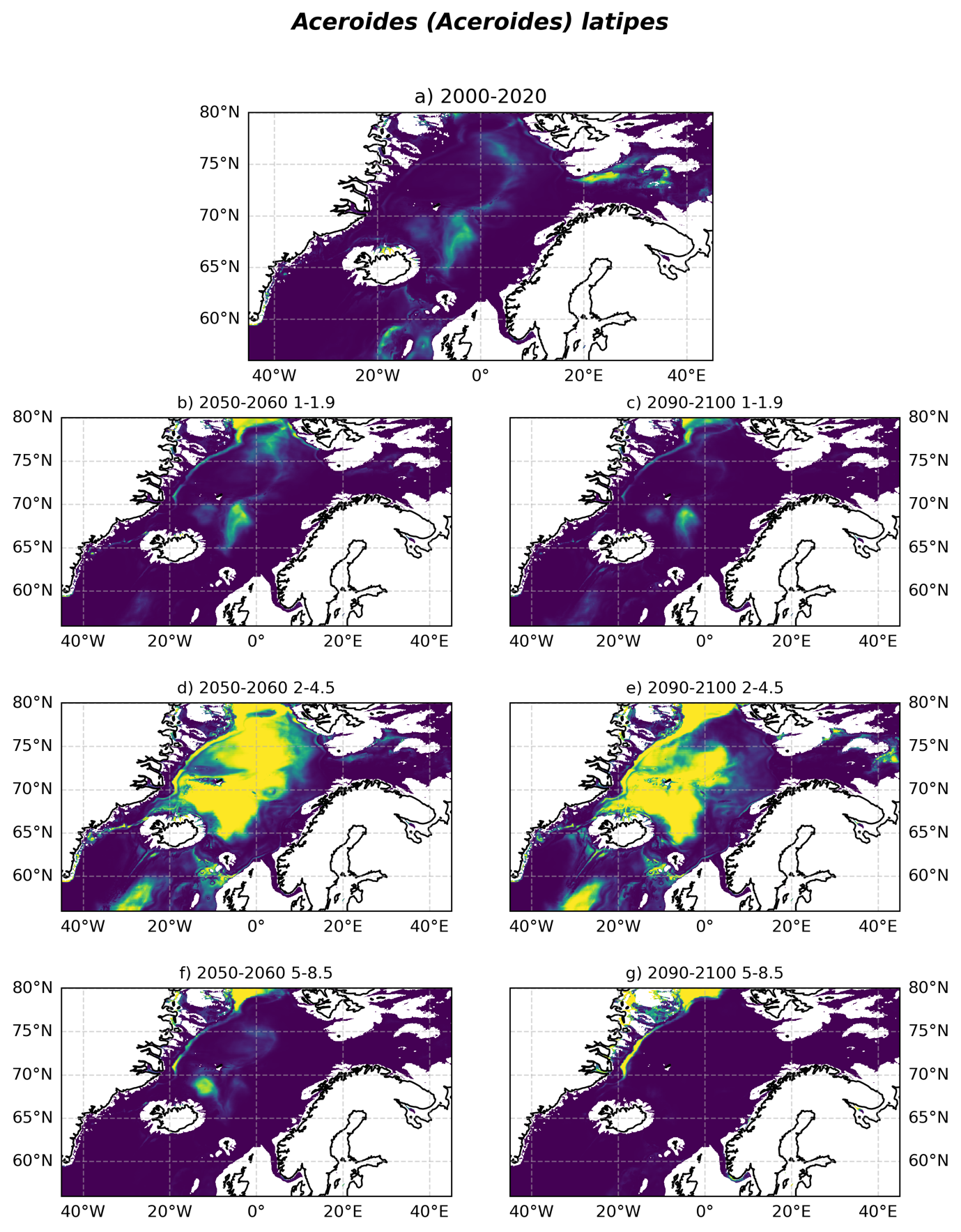


Figure S36: Maps show the habitat suitability of *Aceroides (Aceroides) latipes* for a) present day, b) 2050–2060 and 1–1.9 SSP scenario, c) 2090–2100 and 1–1.9 SSP scenario, d) 2050–2060 and 2–4.5 SSP scenario, e) 2090–2100 and 2–4.5 SSP scenario, f) 2050–2060 and 5–8.5 SSP scenario, and g) 2090–2100 and 5–8.5 SSP. Purple indicating unsuitable habitat, yellow indicating highly suitable habitat.


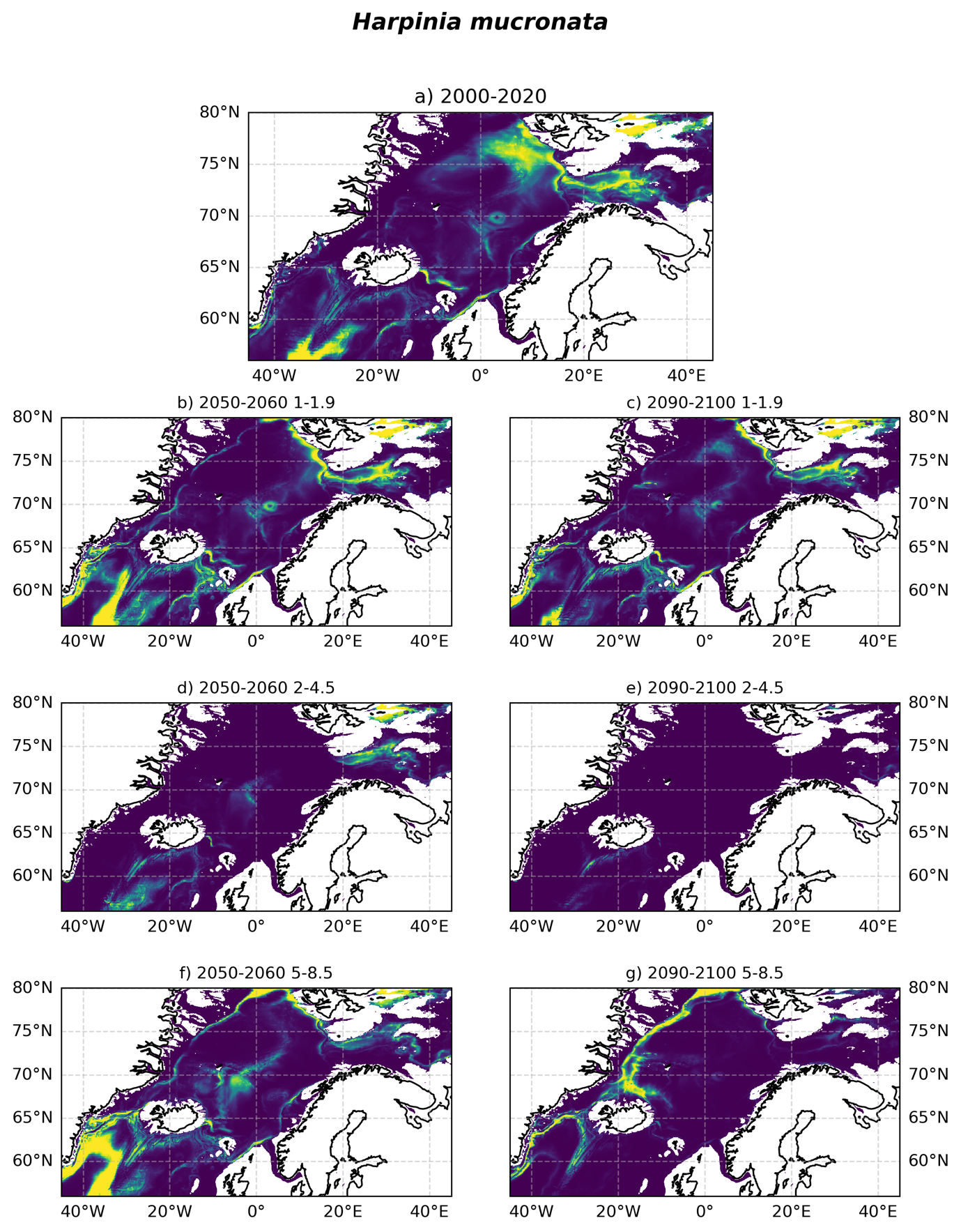


Figure S37: Maps show the habitat suitability of *Harpinia mucronata* for a) present day, b) 2050–2060 and 1–1.9 SSP scenario, c) 2090–2100 and 1–1.9 SSP scenario, d) 2050–2060 and 2–4.5 SSP scenario, e) 2090–2100 and 2–4.5 SSP scenario, f) 2050–2060 and 5–8.5 SSP scenario, and g) 2090–2100 and 5–8.5 SSP. Purple indicating unsuitable habitat, yellow indicating highly suitable habitat.


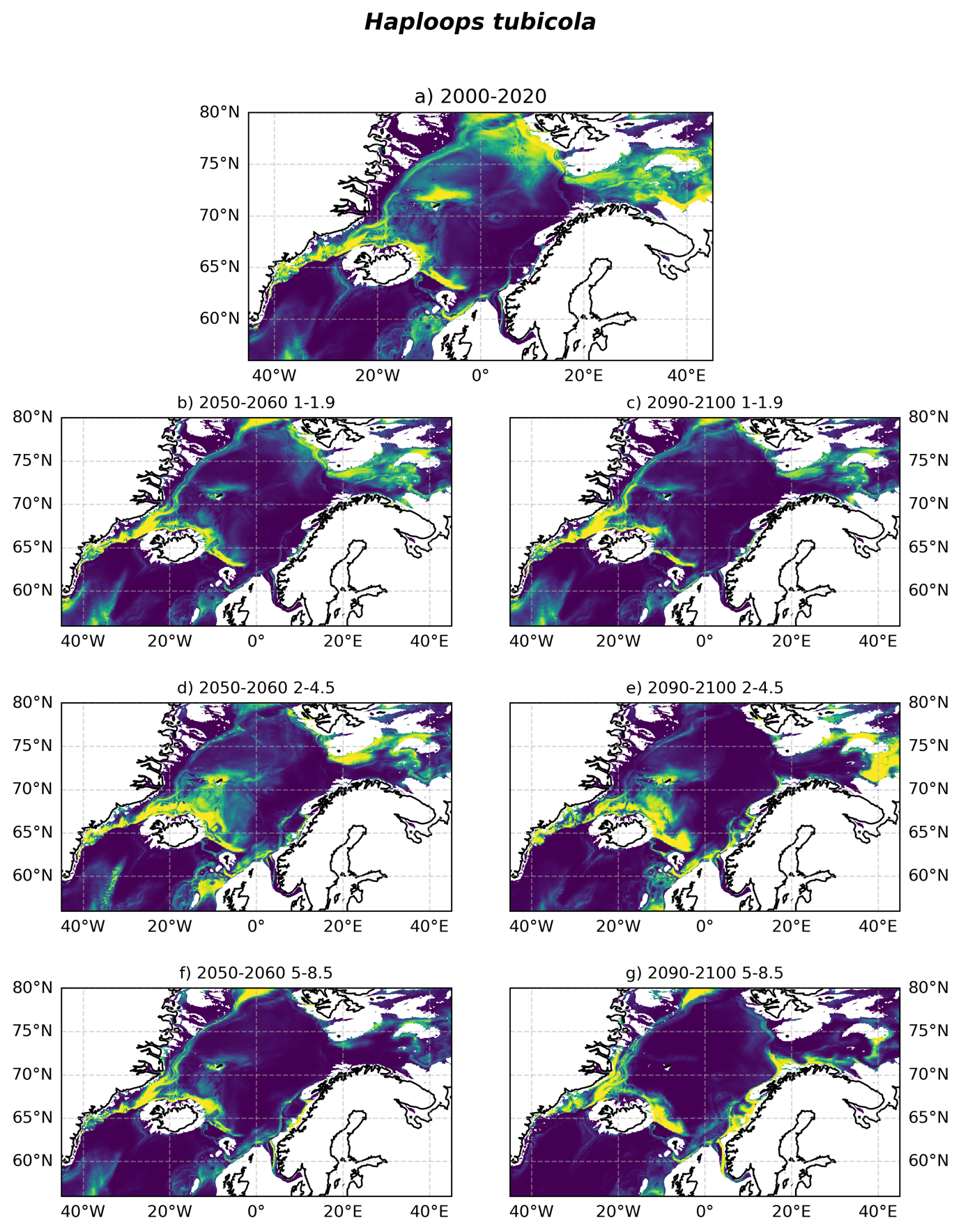


Figure S38: Maps show the habitat suitability of *Haploops tubicola* for a) present day, b) 2050–2060 and 1–1.9 SSP scenario, c) 2090–2100 and 1–1.9 SSP scenario, d) 2050–2060 and 2–4.5 SSP scenario, e) 2090–2100 and 2–4.5 SSP scenario, f) 2050–2060 and 5–8.5 SSP scenario, and g) 2090–2100 and 5–8.5 SSP. Purple indicating unsuitable habitat, yellow indicating highly suitable habitat.


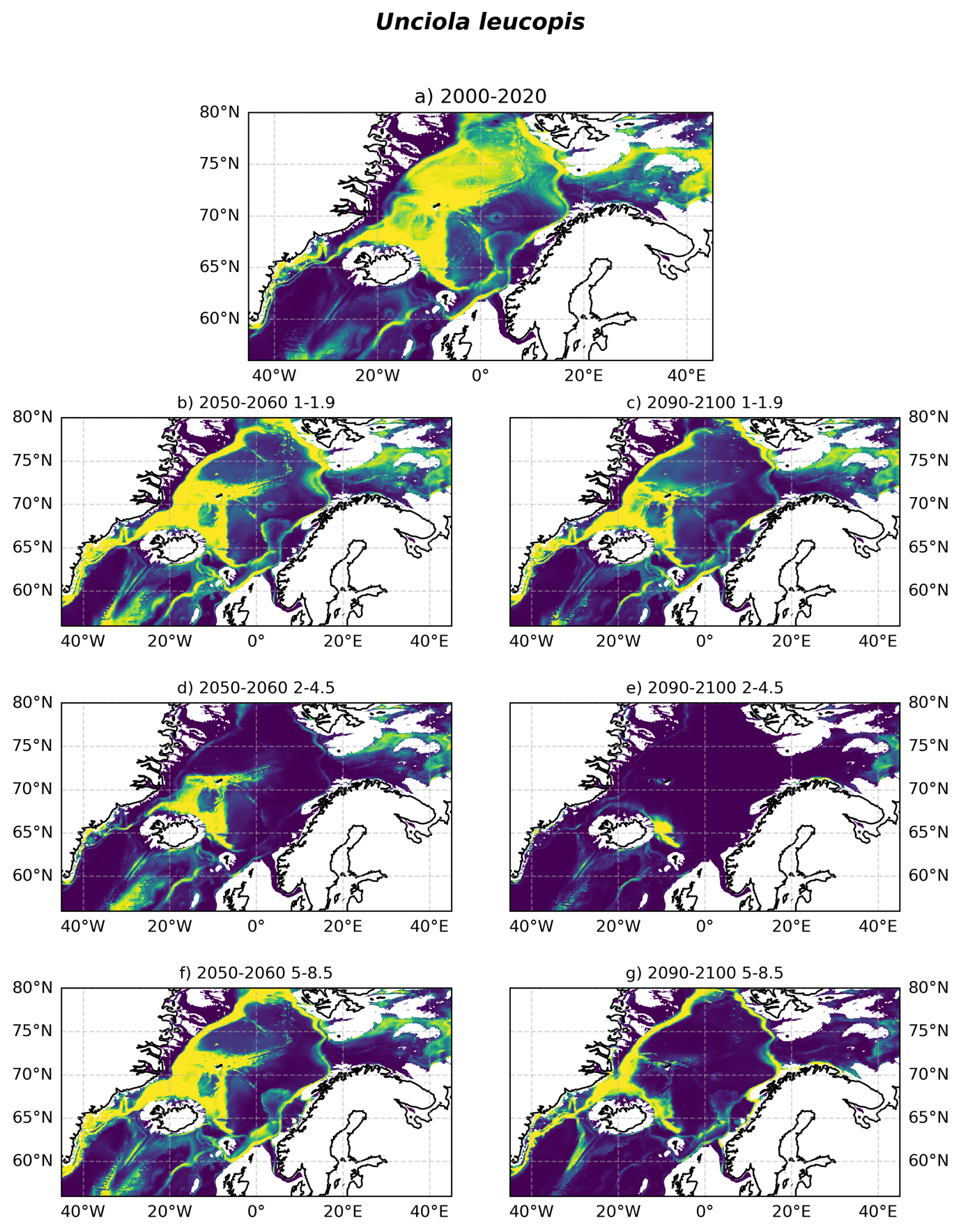


Figure S39: Maps show the habitat suitability of *Unciola leucopis* for a) present day, b) 2050–2060 and 1–1.9 SSP scenario, c) 2090–2100 and 1–1.9 SSP scenario, d) 2050–2060 and 2–4.5 SSP scenario, e) 2090–2100 and 2–4.5 SSP scenario, f) 2050–2060 and 5–8.5 SSP scenario, and g) 2090–2100 and 5–8.5 SSP. Purple indicating unsuitable habitat, yellow indicating highly suitable habitat.


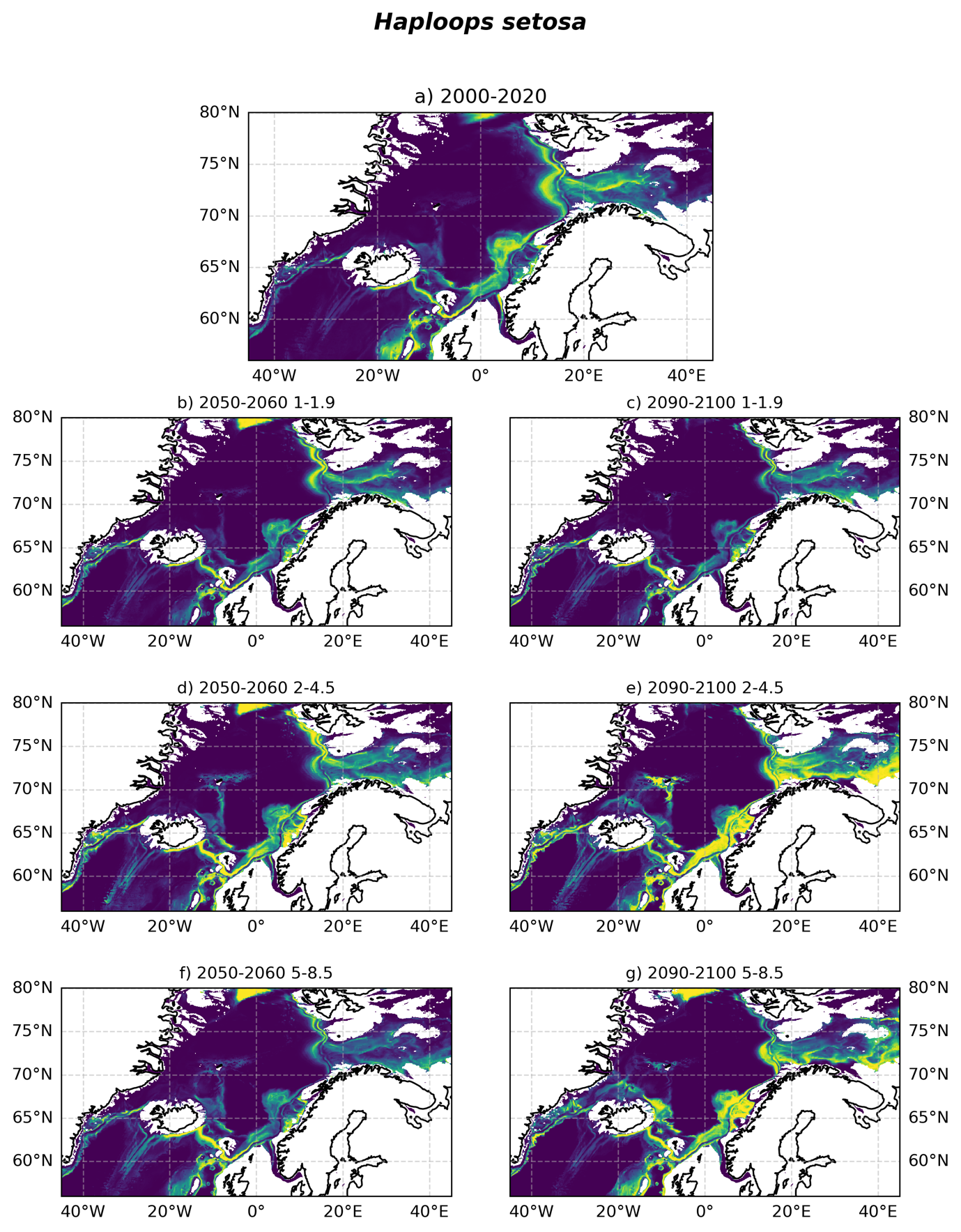


Figure S40: Maps show the habitat suitability of *Haploops setosa* for a) present day, b) 2050–2060 and 1–1.9 SSP scenario, c) 2090–2100 and 1–1.9 SSP scenario, d) 2050–2060 and 2–4.5 SSP scenario, e) 2090–2100 and 2–4.5 SSP scenario, f) 2050–2060 and 5–8.5 SSP scenario, and g) 2090–2100 and 5–8.5 SSP. Purple indicating unsuitable habitat, yellow indicating highly suitable habitat.


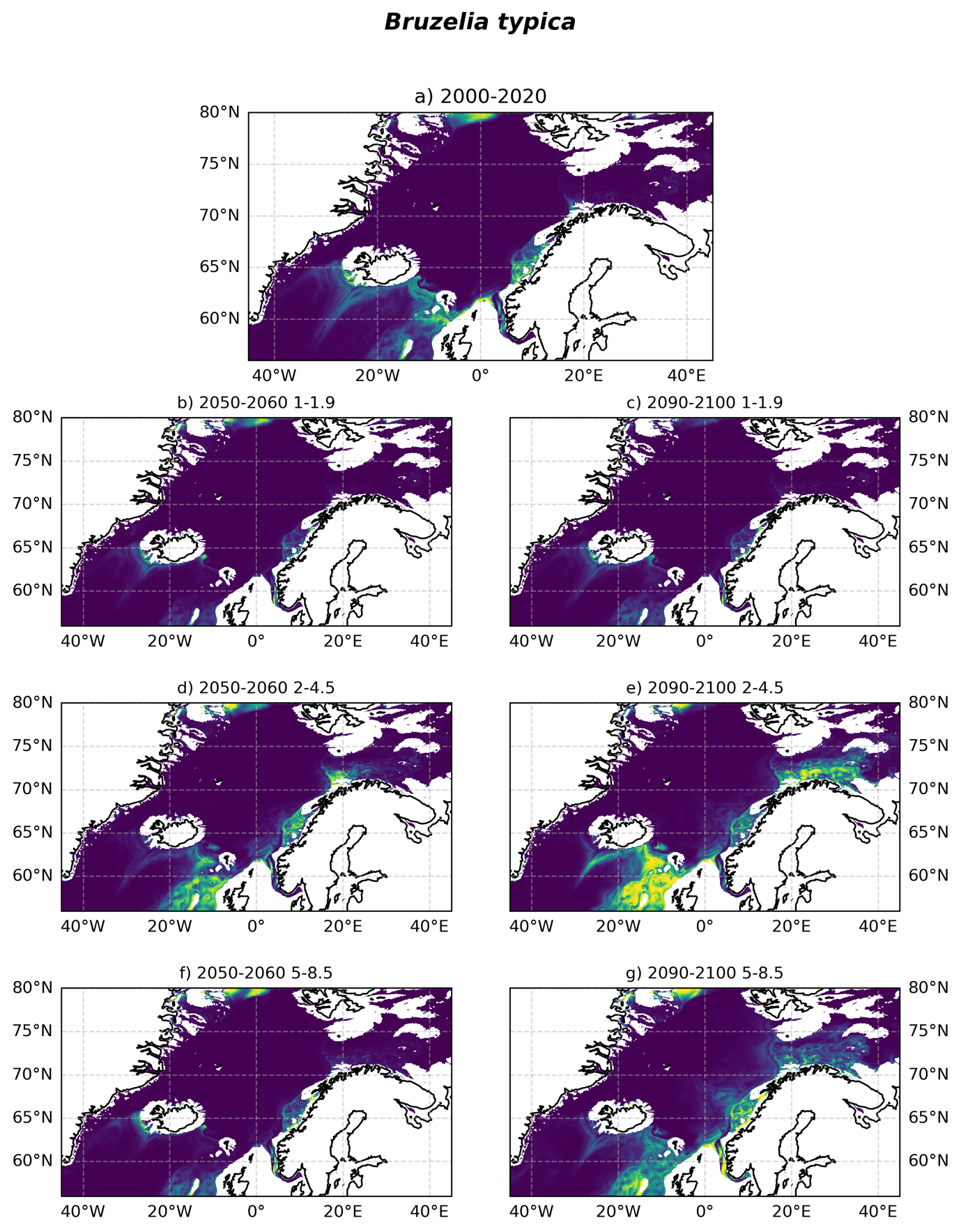


Figure S41: Maps show the habitat suitability of *Bruzelia typica* for a) present day, b) 2050–2060 and 1–1.9 SSP scenario, c) 2090–2100 and 1–1.9 SSP scenario, d) 2050–2060 and 2–4.5 SSP scenario, e) 2090–2100 and 2–4.5 SSP scenario, f) 2050–2060 and 5–8.5 SSP scenario, and g) 2090–2100 and 5–8.5 SSP. Purple indicating unsuitable habitat, yellow indicating highly suitable habitat.


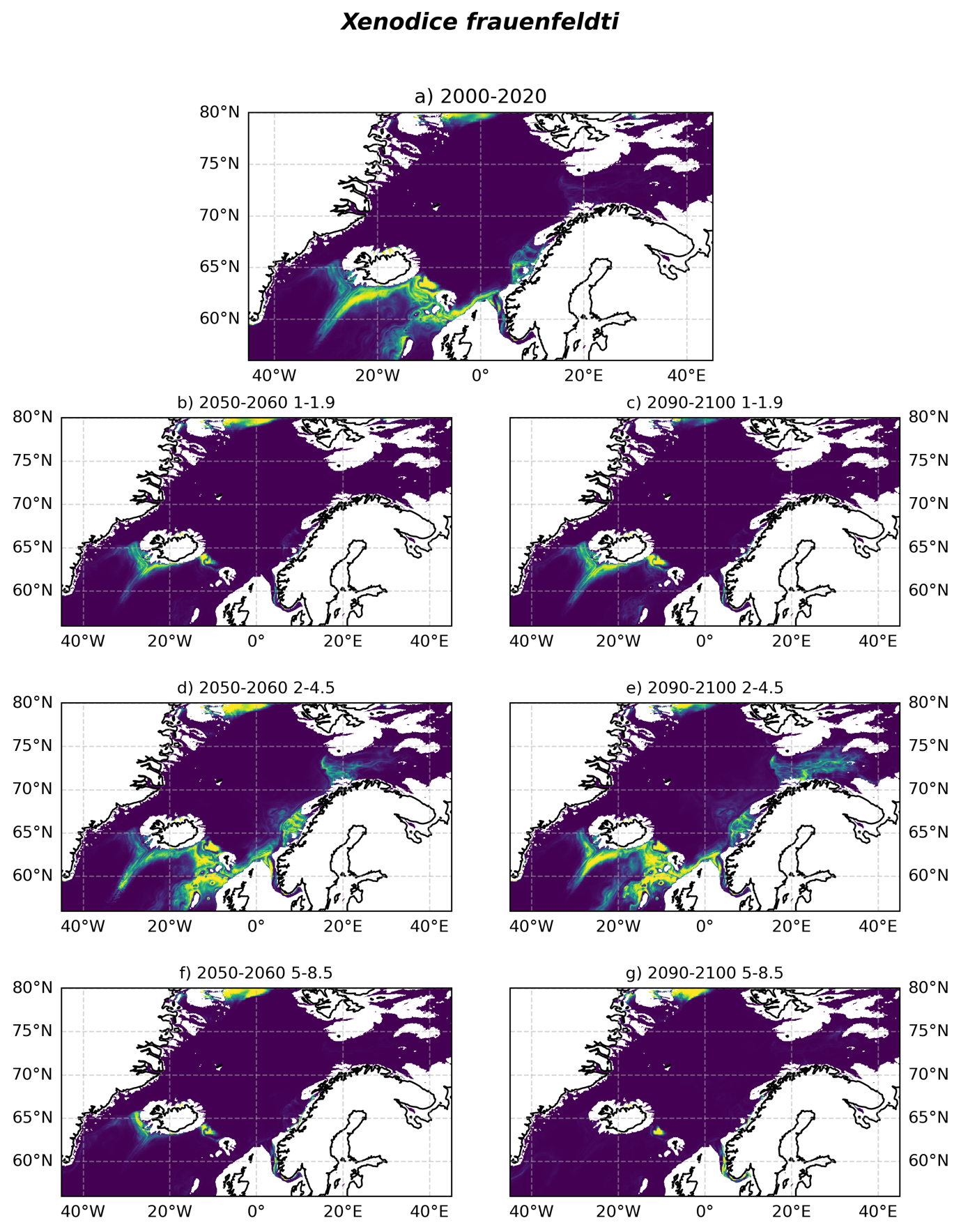


Figure S42: Maps show the habitat suitability of *Xenodice frauenfeldti* for a) present day, b) 2050–2060 and 1–1.9 SSP scenario, c) 2090–2100 and 1–1.9 SSP scenario, d) 2050–2060 and 2–4.5 SSP scenario, e) 2090–2100 and 2–4.5 SSP scenario, f) 2050–2060 and 5–8.5 SSP scenario, and g) 2090–2100 and 5–8.5 SSP. Purple indicating unsuitable habitat, yellow indicating highly suitable habitat.


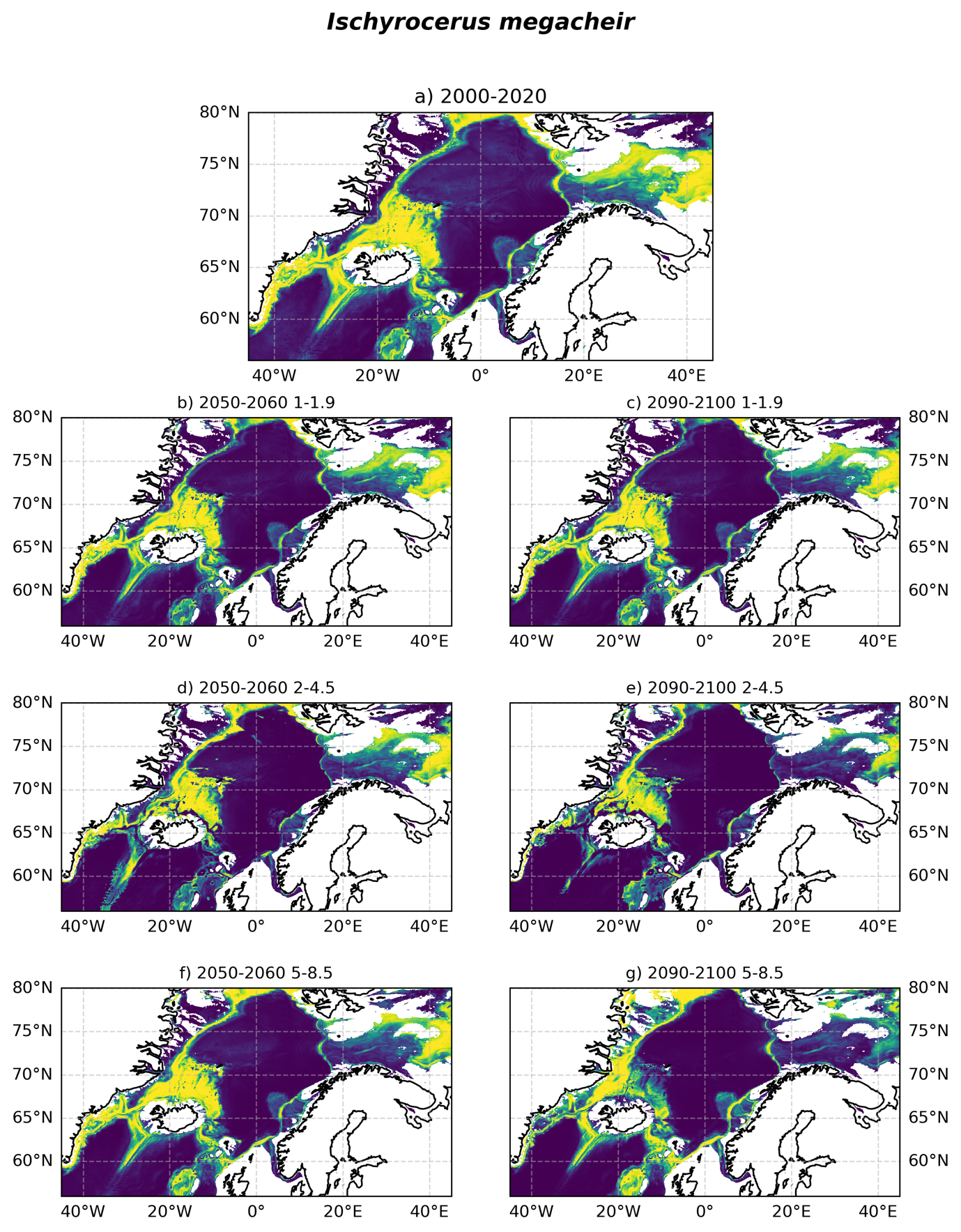


Figure S43: Maps show the habitat suitability of *Ischyrocerus megacheir* for a) present day, b) 2050–2060 and 1–1.9 SSP scenario, c) 2090–2100 and 1–1.9 SSP scenario, d) 2050–2060 and 2–4.5 SSP scenario, e) 2090–2100 and 2–4.5 SSP scenario, f) 2050–2060 and 5–8.5 SSP scenario, and g) 2090–2100 and 5–8.5 SSP. Purple indicating unsuitable habitat, yellow indicating highly suitable habitat.


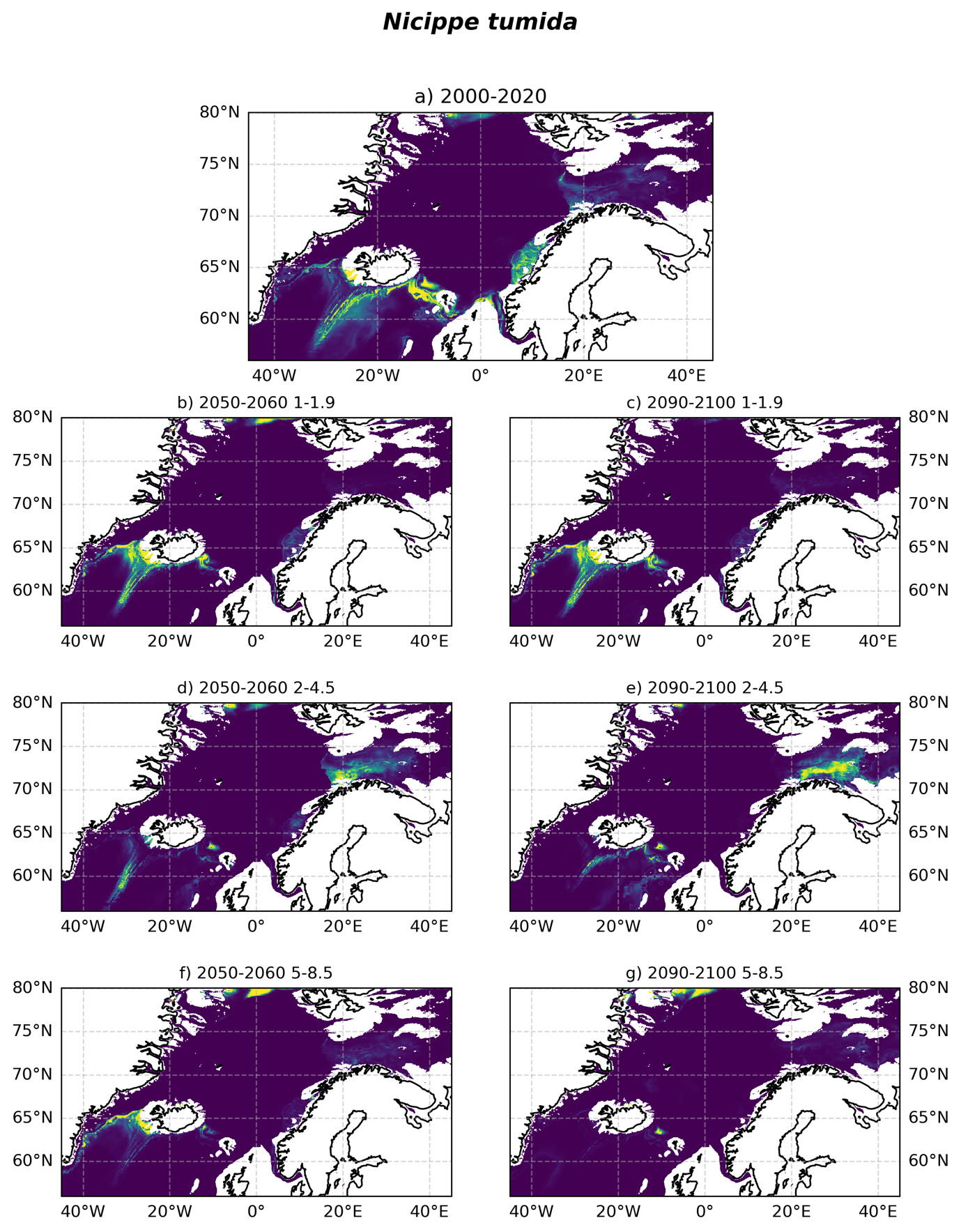


Figure S44: Maps show the habitat suitability of *Nicippe tumida* for a) present day, b) 2050–2060 and 1–1.9 SSP scenario, c) 2090–2100 and 1–1.9 SSP scenario, d) 2050–2060 and 2–4.5 SSP scenario, e) 2090–2100 and 2–4.5 SSP scenario, f) 2050–2060 and 5–8.5 SSP scenario, and g) 2090–2100 and 5–8.5 SSP. Purple indicating unsuitable habitat, yellow indicating highly suitable habitat.


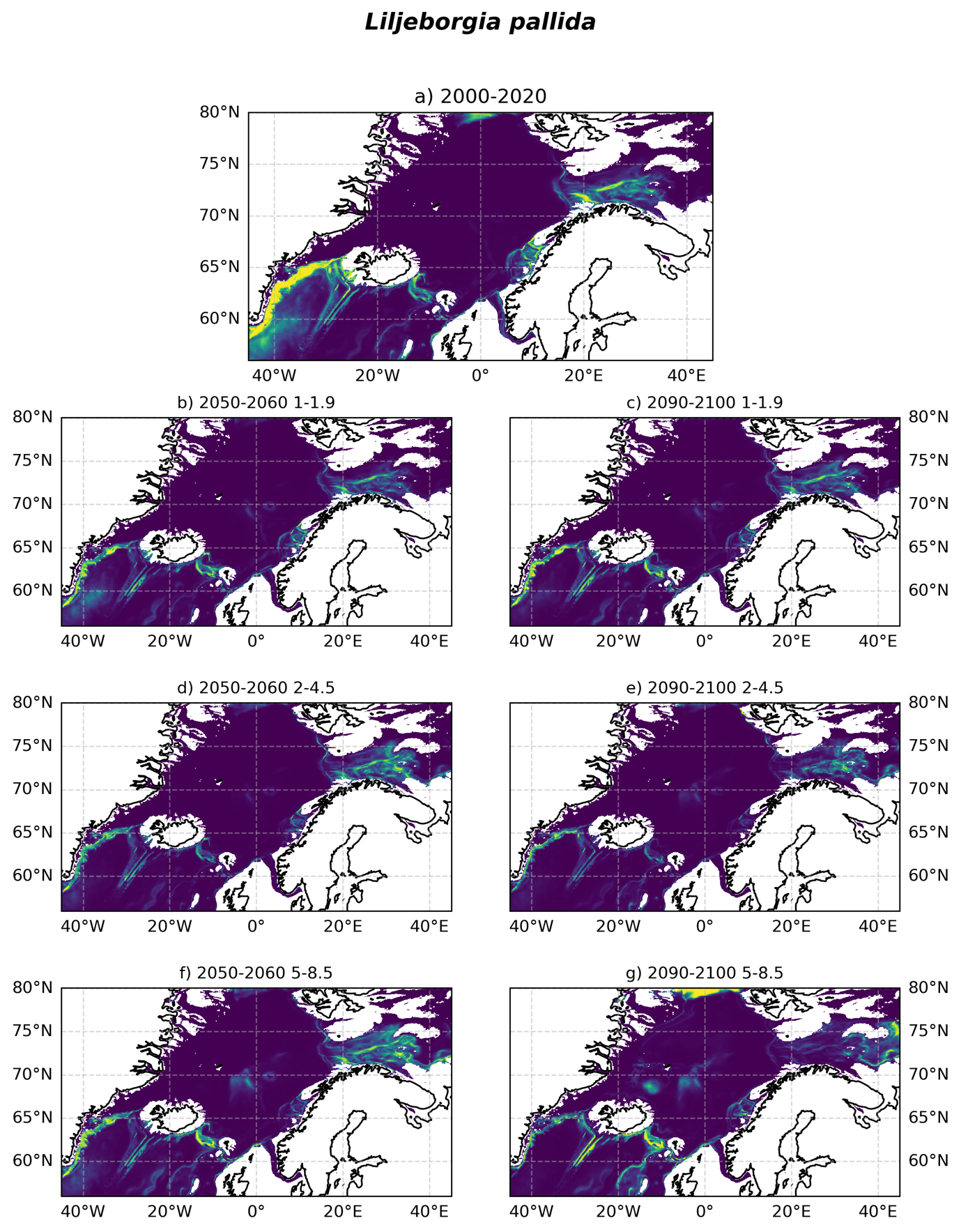


Figure S45: Maps show the habitat suitability of *Liljeborgia pallida* for a) present day, b) 2050–2060 and 1–1.9 SSP scenario, c) 2090–2100 and 1–1.9 SSP scenario, d) 2050–2060 and 2–4.5 SSP scenario, e) 2090–2100 and 2–4.5 SSP scenario, f) 2050–2060 and 5–8.5 SSP scenario, and g) 2090–2100 and 5–8.5 SSP. Purple indicating unsuitable habitat, yellow indicating highly suitable habitat.


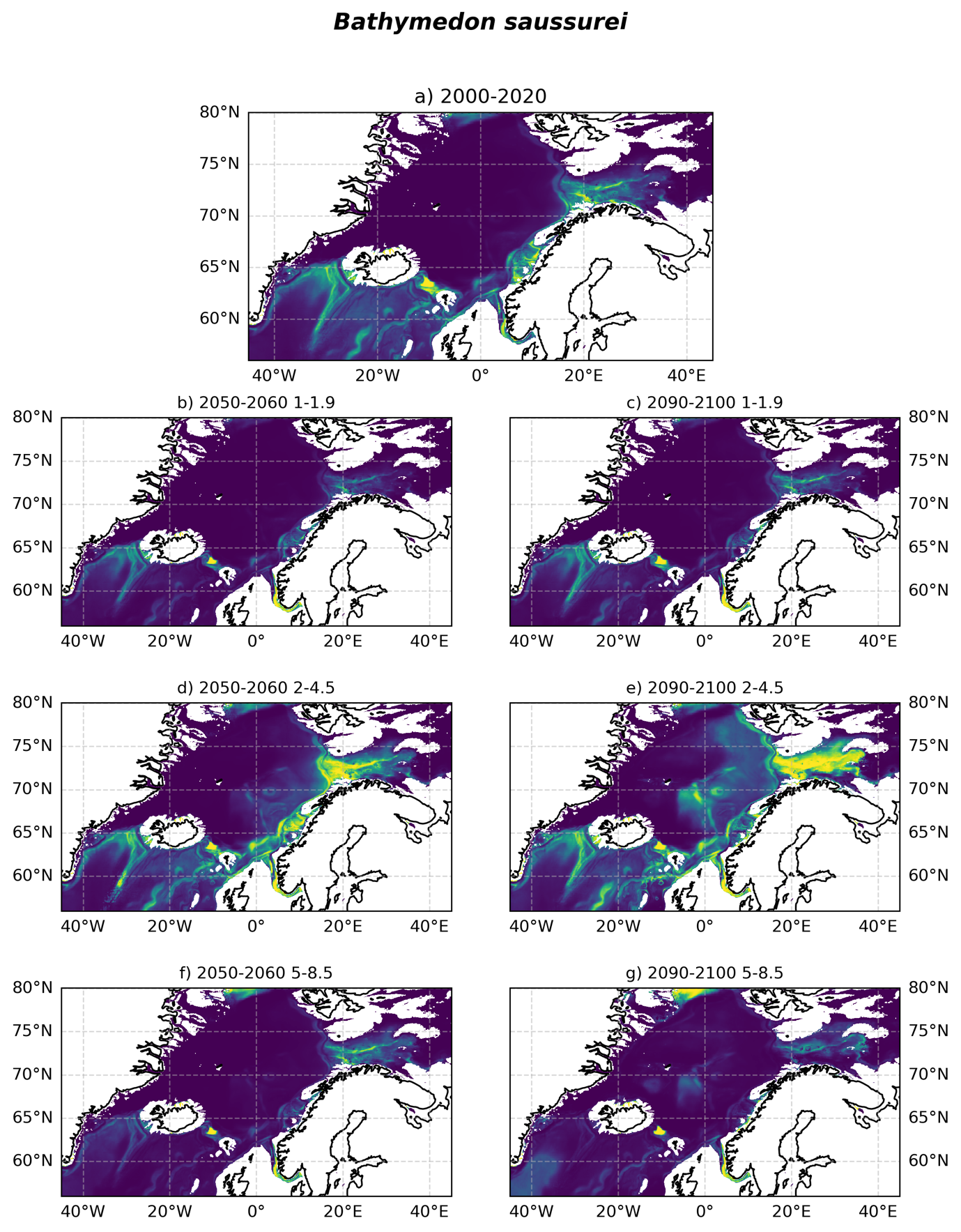


Figure S46: Maps show the habitat suitability of *Bathymedon saussurei* for a) present day, b) 2050–2060 and 1–1.9 SSP scenario, c) 2090–2100 and 1–1.9 SSP scenario, d) 2050–2060 and 2–4.5 SSP scenario, e) 2090–2100 and 2–4.5 SSP scenario, f) 2050–2060 and 5–8.5 SSP scenario, and g) 2090–2100 and 5–8.5 SSP. Purple indicating unsuitable habitat, yellow indicating highly suitable habitat.


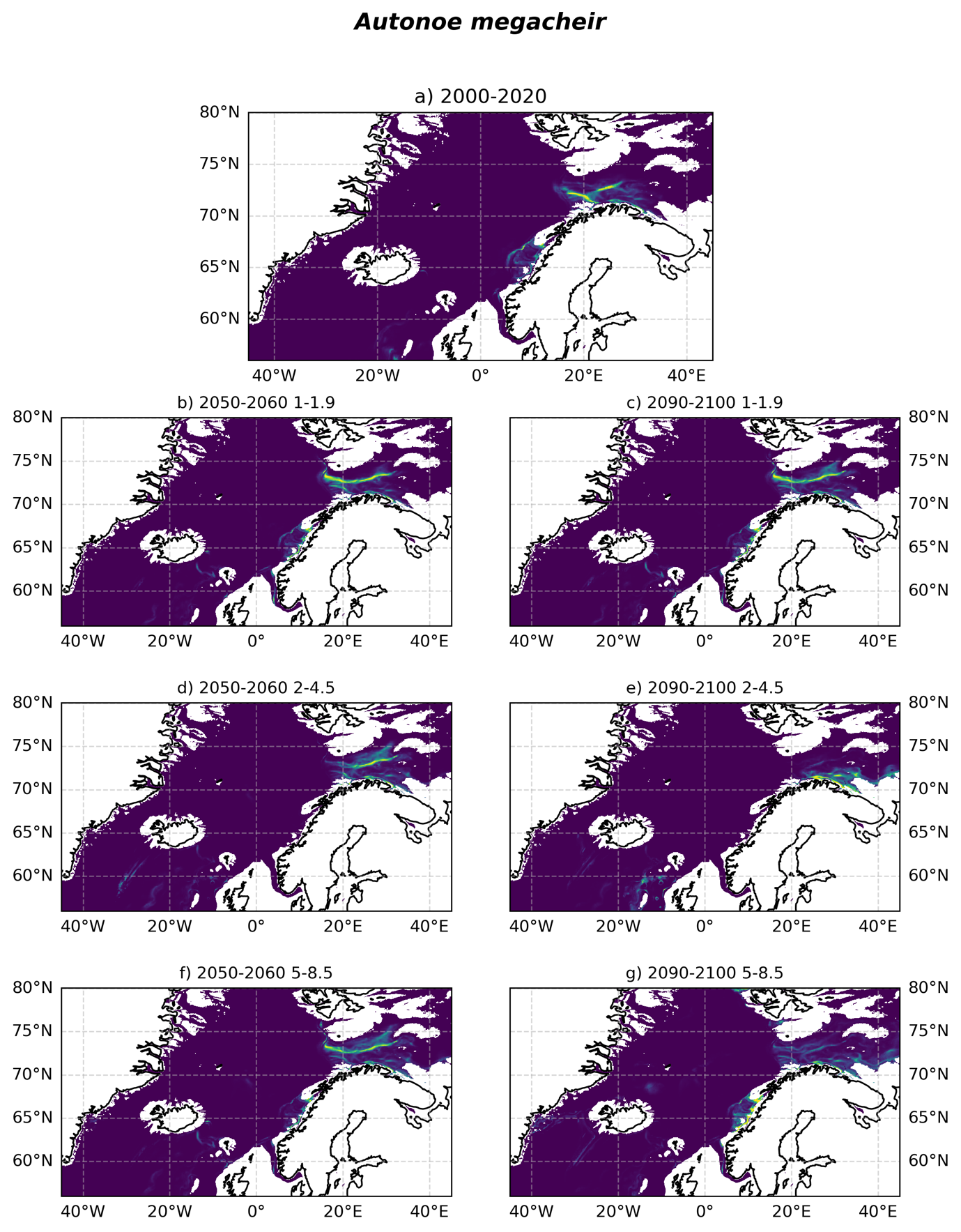


Figure S47: Maps show the habitat suitability of *Autonoe megacheir* for a) present day, b) 2050–2060 and 1–1.9 SSP scenario, c) 2090–2100 and 1–1.9 SSP scenario, d) 2050–2060 and 2–4.5 SSP scenario, e) 2090–2100 and 2–4.5 SSP scenario, f) 2050–2060 and 5–8.5 SSP scenario, and g) 2090–2100 and 5–8.5 SSP. Purple indicating unsuitable habitat, yellow indicating highly suitable habitat.


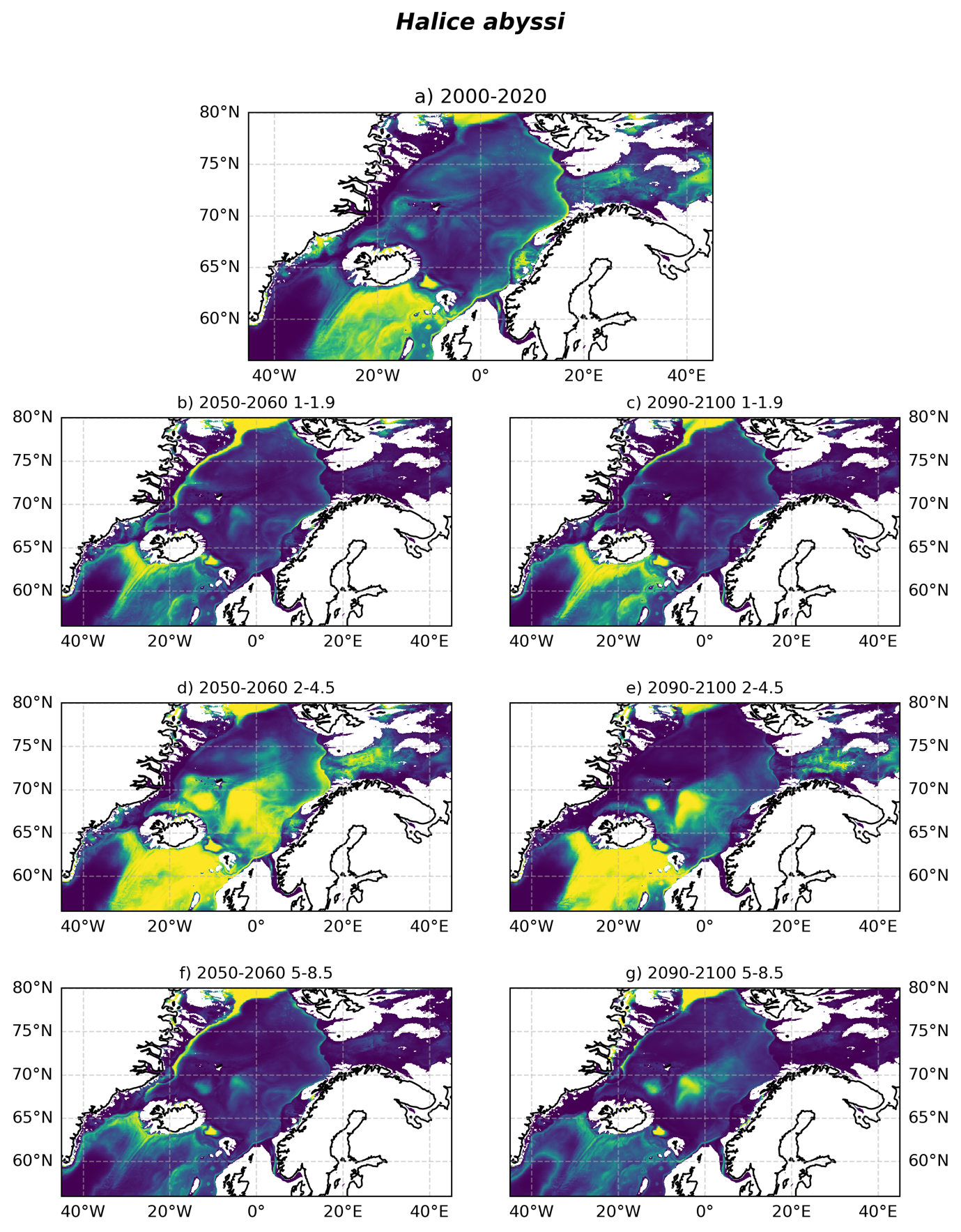


Figure S48: Maps show the habitat suitability of *Halice abyssi* for a) present day, b) 2050–2060 and 1–1.9 SSP scenario, c) 2090–2100 and 1–1.9 SSP scenario, d) 2050–2060 and 2–4.5 SSP scenario, e) 2090–2100 and 2–4.5 SSP scenario, f) 2050–2060 and 5–8.5 SSP scenario, and g) 2090–2100 and 5–8.5 SSP. Purple indicating unsuitable habitat, yellow indicating highly suitable habitat.


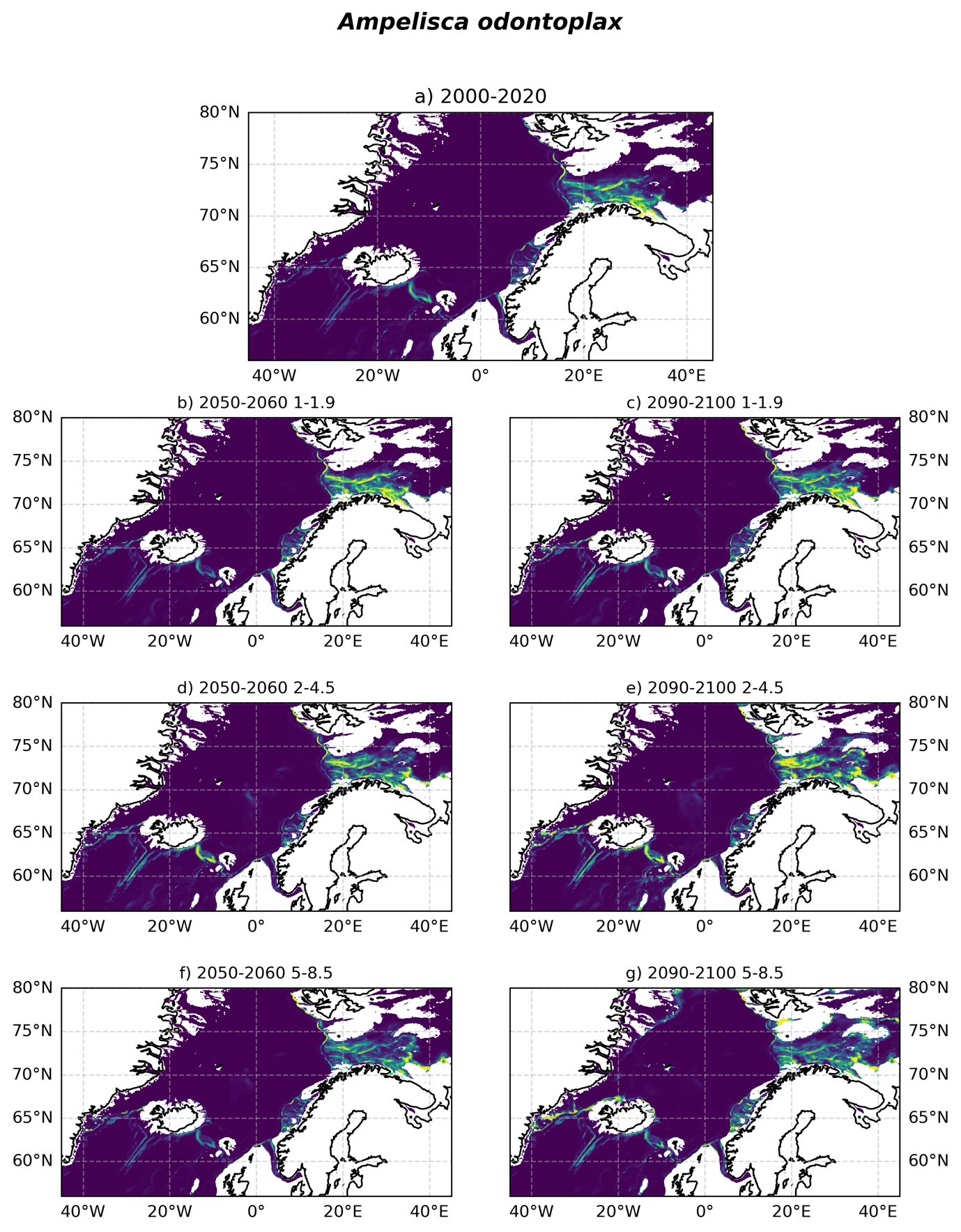


Figure S49: Maps show the habitat suitability of *Ampelisca odontoplax* for a) present day, b) 2050–2060 and 1–1.9 SSP scenario, c) 2090–2100 and 1–1.9 SSP scenario, d) 2050–2060 and 2–4.5 SSP scenario, e) 2090–2100 and 2–4.5 SSP scenario, f) 2050–2060 and 5–8.5 SSP scenario, and g) 2090–2100 and 5–8.5 SSP. Purple indicating unsuitable habitat, yellow indicating highly suitable habitat.


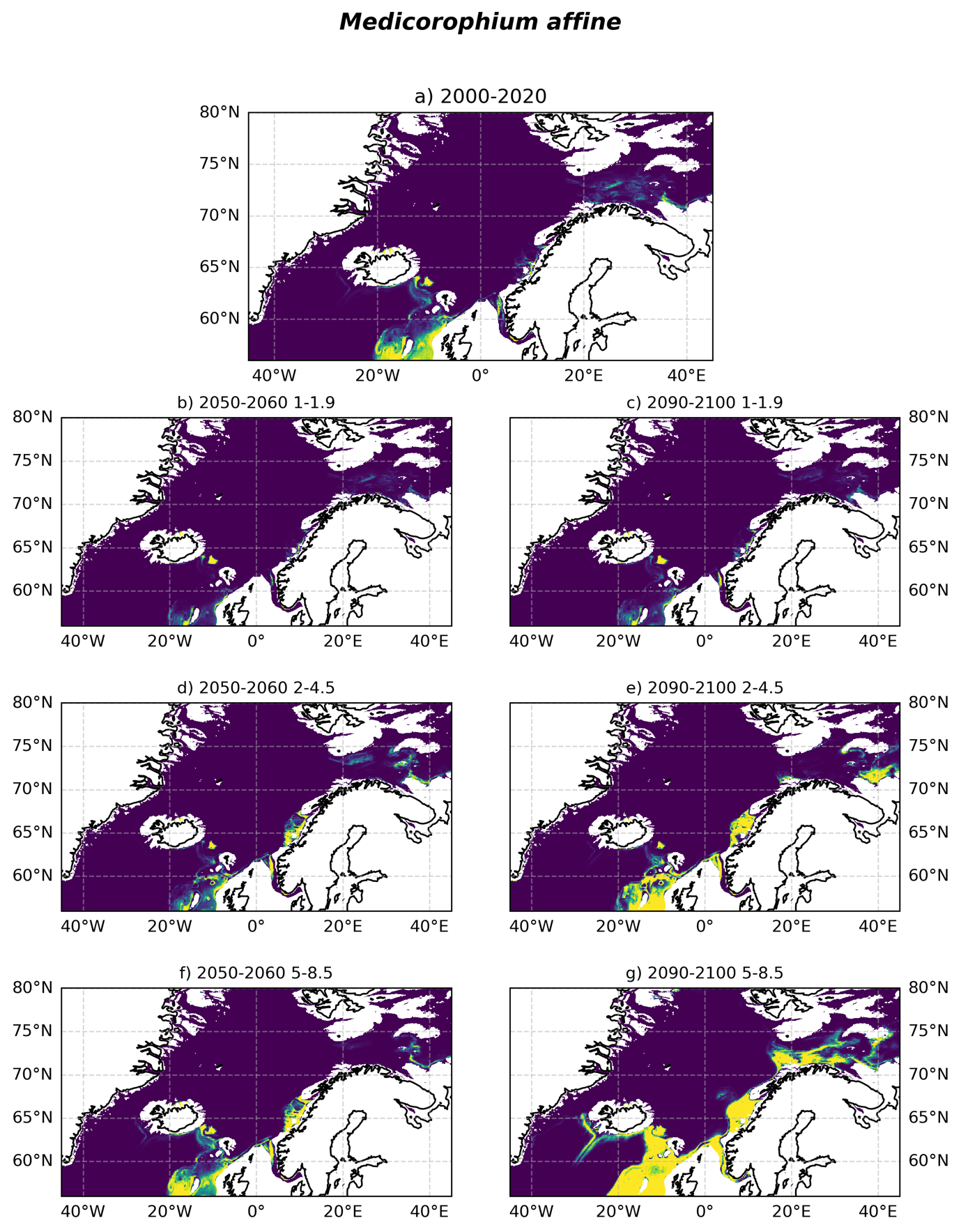


Figure S50: Maps show the habitat suitability of *Medicorophium affine* for a) present day, b) 2050–2060 and 1–1.9 SSP scenario, c) 2090–2100 and 1–1.9 SSP scenario, d) 2050–2060 and 2–4.5 SSP scenario, e) 2090–2100 and 2–4.5 SSP scenario, f) 2050–2060 and 5–8.5 SSP scenario, and g) 2090–2100 and 5–8.5 SSP. Purple indicating unsuitable habitat, yellow indicating highly suitable habitat.


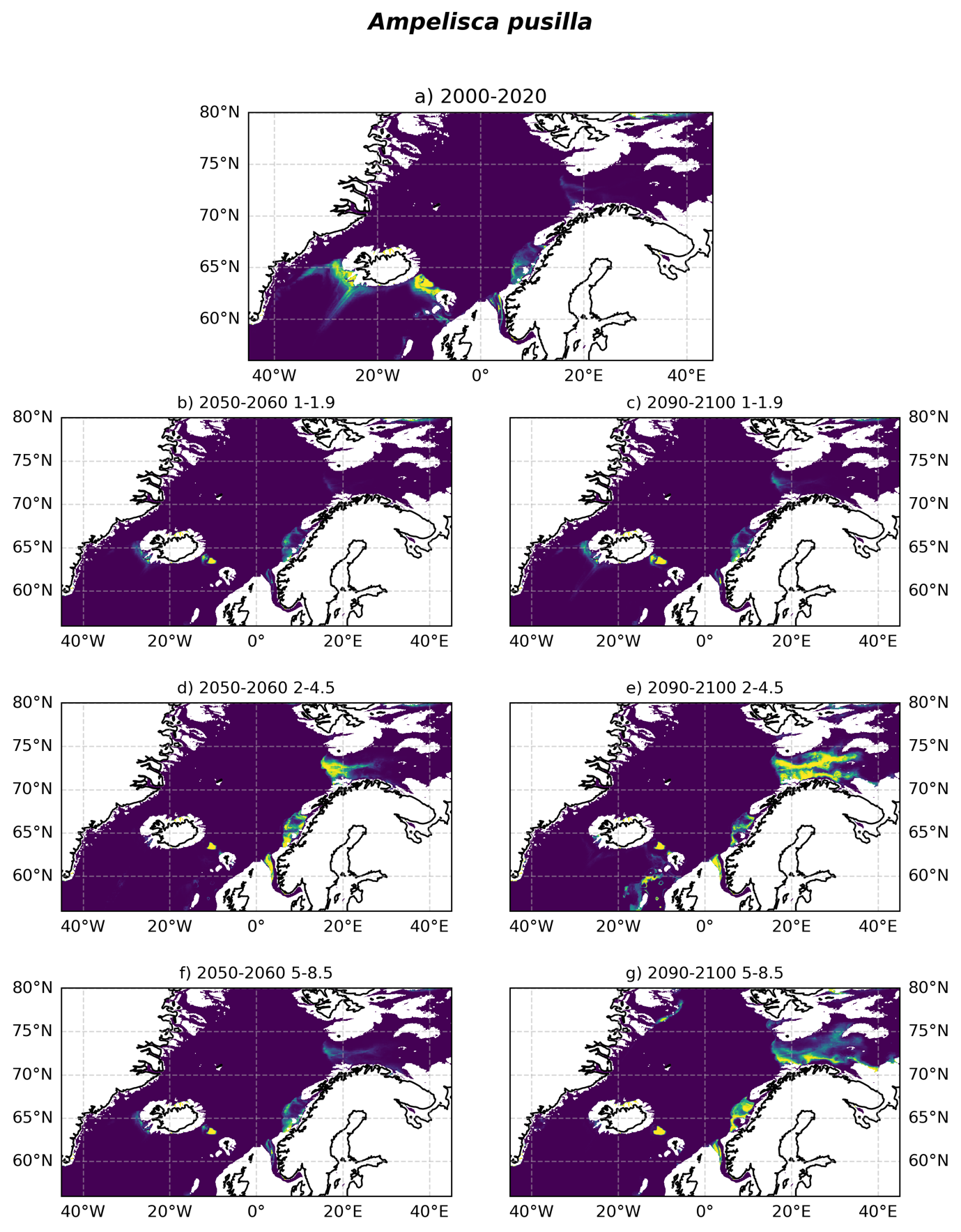


Figure S51: Maps show the habitat suitability of *Ampelisca pusilla* for a) present day, b) 2050–2060 and 1–1.9 SSP scenario, c) 2090–2100 and 1–1.9 SSP scenario, d) 2050–2060 and 2–4.5 SSP scenario, e) 2090–2100 and 2–4.5 SSP scenario, f) 2050–2060 and 5–8.5 SSP scenario, and g) 2090–2100 and 5–8.5 SSP. Purple indicating unsuitable habitat, yellow indicating highly suitable habitat.


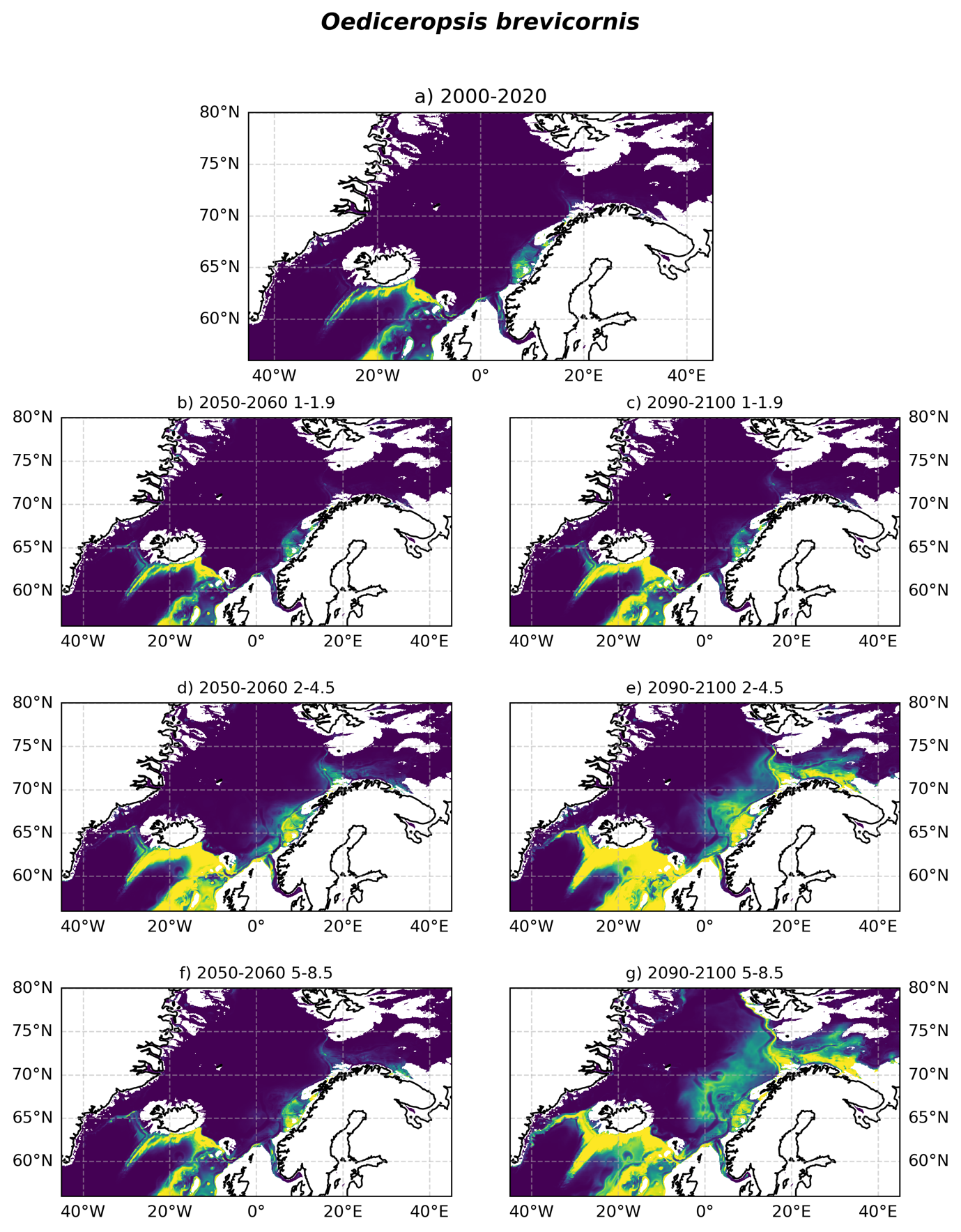


Figure S52: Maps show the habitat suitability of *Oediceropsis brevicornis* for a) present day, b) 2050–2060 and 1–1.9 SSP scenario, c) 2090–2100 and 1–1.9 SSP scenario, d) 2050–2060 and 2–4.5 SSP scenario, e) 2090–2100 and 2–4.5 SSP scenario, f) 2050–2060 and 5–8.5 SSP scenario, and g) 2090–2100 and 5–8.5 SSP. Purple indicating unsuitable habitat, yellow indicating highly suitable habitat.


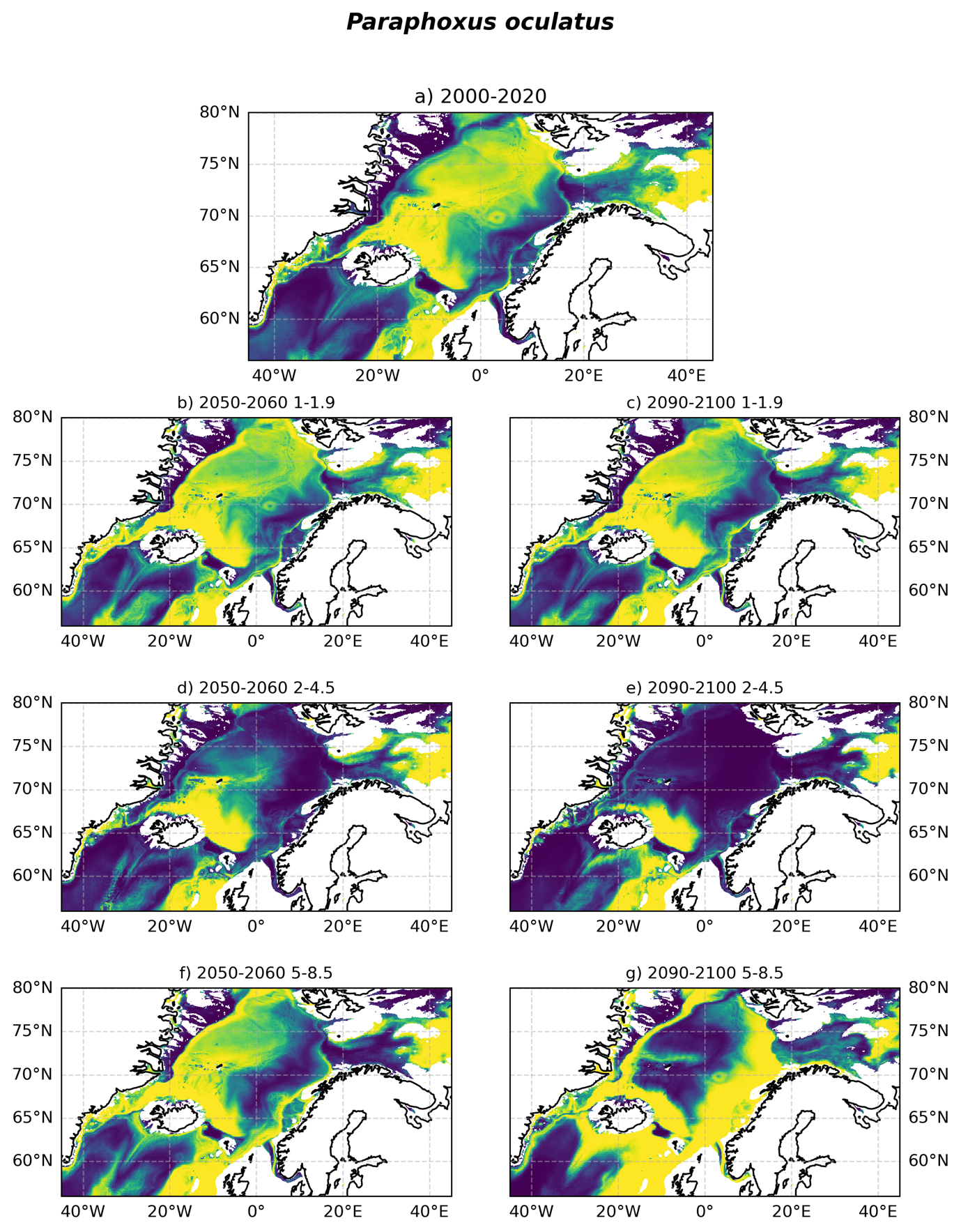


Figure S53: Maps show the habitat suitability of *Paraphoxus oculatus* for a) present day, b) 2050–2060 and 1–1.9 SSP scenario, c) 2090–2100 and 1–1.9 SSP scenario, d) 2050–2060 and 2–4.5 SSP scenario, e) 2090–2100 and 2–4.5 SSP scenario, f) 2050–2060 and 5–8.5 SSP scenario, and g) 2090–2100 and 5–8.5 SSP. Purple indicating unsuitable habitat, yellow indicating highly suitable habitat.


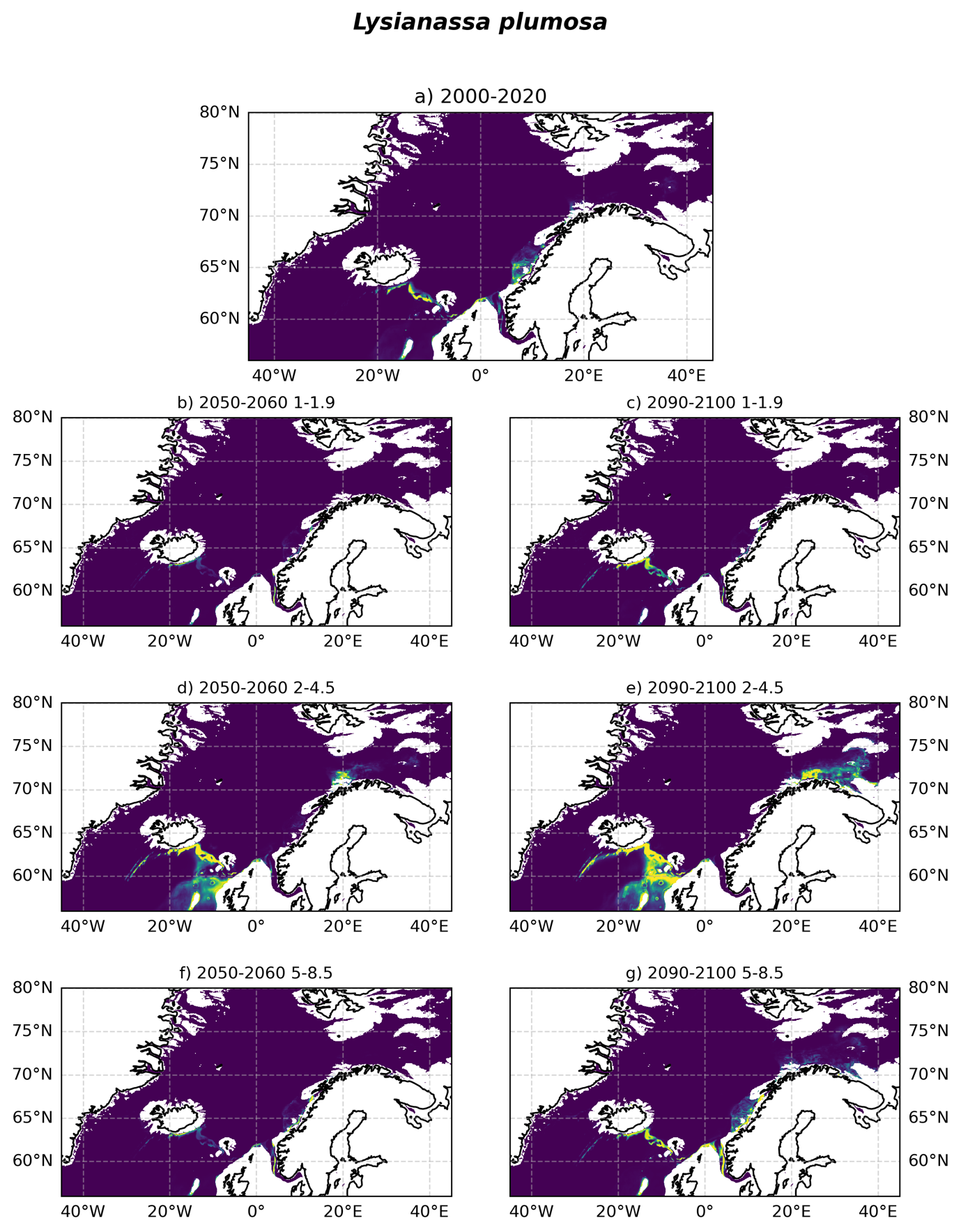


Figure S54: Maps show the habitat suitability of *Lysianassa plumosa* for a) present day, b) 2050–2060 and 1–1.9 SSP scenario, c) 2090–2100 and 1–1.9 SSP scenario, d) 2050–2060 and 2–4.5 SSP scenario, e) 2090–2100 and 2–4.5 SSP scenario, f) 2050–2060 and 5–8.5 SSP scenario, and g) 2090–2100 and 5–8.5 SSP. Purple indicating unsuitable habitat, yellow indicating highly suitable habitat.


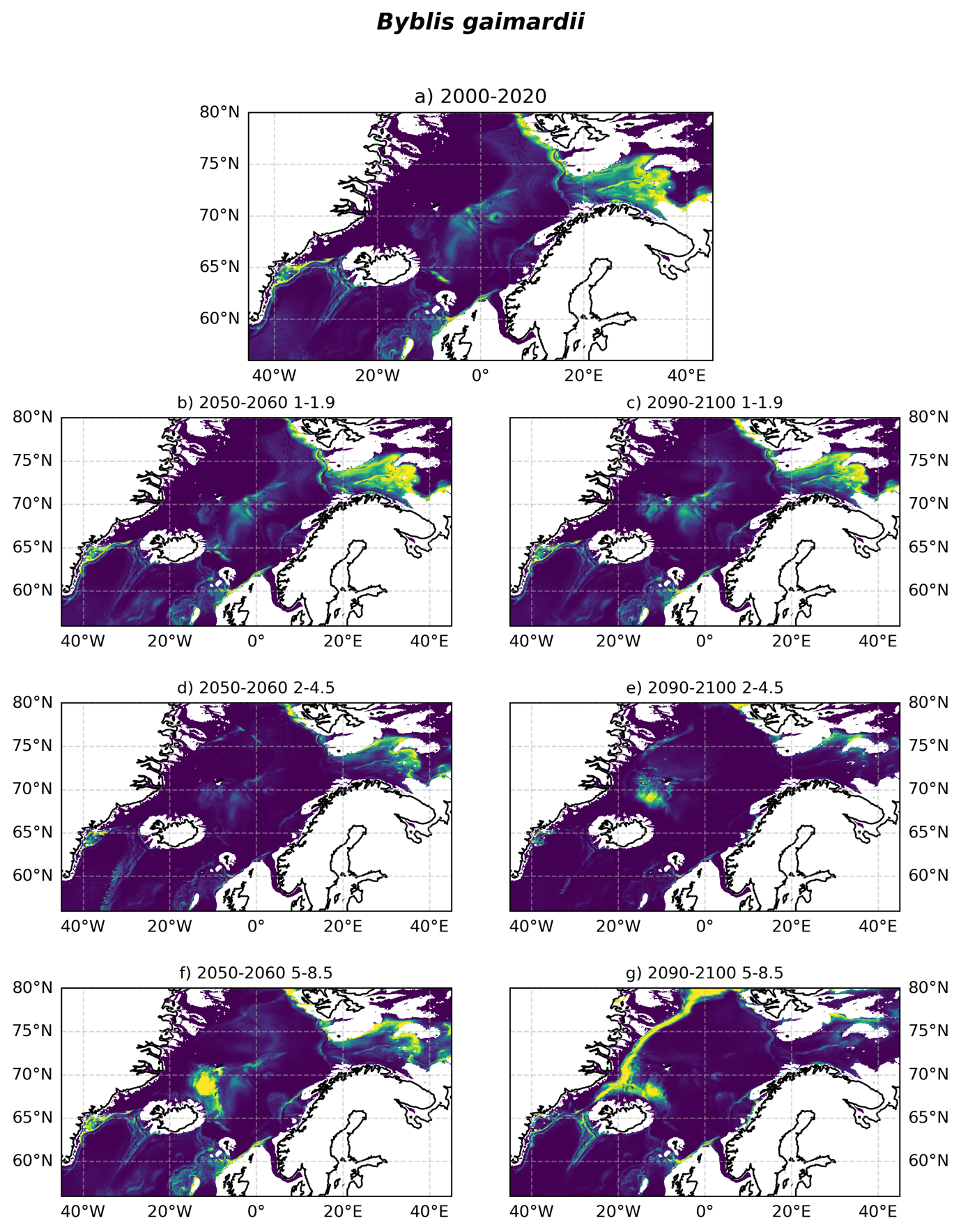


Figure S55: Maps show the habitat suitability of *Byblis gaimardii* for a) present day, b) 2050–2060 and 1–1.9 SSP scenario, c) 2090–2100 and 1–1.9 SSP scenario, d) 2050–2060 and 2–4.5 SSP scenario, e) 2090–2100 and 2–4.5 SSP scenario, f) 2050–2060 and 5–8.5 SSP scenario, and g) 2090–2100 and 5–8.5 SSP. Purple indicating unsuitable habitat, yellow indicating highly suitable habitat.

**References**

GEBCO Compilation Group. (2024). *GEBCO 2024 Grid*. doi:10.5285/1c44ce99-0a0d-5f4f-e063-7086abc0ea0f.
